# The IκB Kinase Inhibitor ACHP Targets the STAT3 Signaling Pathway in Human Non-Small Cell Lung Carcinoma Cells

**DOI:** 10.3390/biom9120875

**Published:** 2019-12-13

**Authors:** Jong Hyun Lee, Chakrabhavi Dhananjaya Mohan, Salundi Basappa, Shobith Rangappa, Arunachalam Chinnathambi, Tahani Awad Alahmadi, Sulaiman Ali Alharbi, Alan Prem Kumar, Gautam Sethi, Kwang Seok Ahn, Kanchugarakoppal S Rangappa

**Affiliations:** 1 College of Korean Medicine, Kyung Hee University, #47, Kyungheedae-gil, Dongdaemoon-gu, Seoul 130-701, Korea; 88mirue@gmail.com; 2 Department of Studies in Molecular Biology, University of Mysore, Manasagangotri, Mysore 570006, India; cd.mohan@yahoo.com; 3 Laboratory of Chemical Biology, Department of Studies in Organic Chemistry, University of Mysore, Manasagangotri, Mysore 570006, India; salundibasappa@gmail.com; 4 Adichunchanagiri Institute for Molecular Medicine, BG Nagara-571448, Nagamangala Taluk, Mandya District 571448, India; shobithrangappa@gmail.com; 5 Department of Botany and Microbiology, College of Science, King Saud University, Riyadh 11451, Saudi Arabia; talahmadi@ksu.edu.sa (T.A.A.); sharbi@ksu.edu.sa (S.A.A.); 6 Department of Pediatrics, College of Medicine and King Khalid University Hospital, King Saud University Medical City, Riyadh 11461, Saudi Arabia; 7 Cancer Science Institute of Singapore, National University of Singapore, Singapore 117599, Singapore; csiapk@nus.edu.sg; 8 Department of Pharmacology, Yong Loo Lin School of Medicine, National University of Singapore, Singapore 117600, Singapore; phcgs@nus.edu.sg; 9 Institution of Excellence, Vijnana Bhavan, University of Mysore, Manasagangotri, Mysore 570006, India

**Keywords:** ACHP, STAT3 signaling inhibitor, NSCLC, cytotoxicity

## Abstract

STAT3 is an oncogenic transcription factor that regulates the expression of genes which are involved in malignant transformation. Aberrant activation of STAT3 has been observed in a wide range of human malignancies and its role in negative prognosis is well-documented. In this report, we performed high-throughput virtual screening in search of STAT3 signaling inhibitors using a cheminformatics platform and identified 2-Amino-6-[2-(Cyclopropylmethoxy)-6-Hydroxyphenyl]-4-Piperidin-4-yl Nicotinonitrile (ACHP) as the inhibitor of the STAT3 signaling pathway. The predicted hit was evaluated in non-small cell lung cancer (NSCLC) cell lines for its STAT3 inhibitory activity. In vitro experiments suggested that ACHP decreased the cell viability and inhibited the phosphorylation of STAT3 on Tyr705 of NSCLC cells. In addition, ACHP imparted inhibitory activity on the constitutive activation of upstream protein tyrosine kinases, including JAK1, JAK2, and Src. ACHP decreased the nuclear translocation of STAT3 and downregulated its DNA binding ability. Apoptosis was evidenced by cleavage of caspase-3 and PARP with the subsequent decline in antiapoptotic proteins, including Bcl-2, Bcl-xl, and survivin. Overall, we report that ACHP can act as a potent STAT3 signaling inhibitor in NSCLC cell lines.

## 1. Introduction

Lung cancer is the second most common type of cancer in both sexes and a leading cause of cancer-related deaths [1,2,3,4]. Non-small cell lung cancer (NSCLC) and small cell lung carcinoma are the two major subtypes, which account for about 80–85% and 10–15% of all lung cancer, respectively [5,6,7,8,9,10]. The development and progression of NSCLC are tightly associated with smoking, exposure to asbestos and radon, drinking of arsenic-contaminated water, family history, and inhalation of carcinogens, such as beryllium, mustard gas, cadmium, nickel, etc. [11]. Surgical approaches, such as segmentectomy, sleeve resection, lobectomy, pneumonectomy, and non-surgical approaches, including radiation therapy, chemotherapy, and immunotherapy, have been implemented as the treatment strategies in NSCLC [12,13]. The early diagnosis and treatment of NSCLC can contribute to better survival rates and prognosis.

Signal transducer and activator of transcription (STAT) is a family of cytoplasmic transcription factors comprising of seven variants (STAT1, STAT2, STAT3, STAT4, STAT5a, STAT5b, and STAT6). STAT3 is a latent oncogenic protein transiently activated in various types of normal cells [14,15,16,17,18]. The stimulation of transmembrane receptors by cytokines (IL-6 family members) or growth factors (EGF and HGF) lead to the activation of a non-receptor tyrosine kinase, such as Src and Janus kinase (JAK). The activated upstream kinases phosphorylate STAT3 at Tyr705 to undergo dimerization, and translocation into nucleus to transcribe the genes that are involved in proliferation (cyclin D1/E1), inflammation (COX2, IL-1/6 and M-CSF), antiapoptosis (survivin and Bcl-xL), angiogenesis (VEGF, bFGF, and HIF1α), metastasis (MMP2/9), and tumor evasion (IP-10 and RANTES) [19,20,21,22]. Overactivation of STAT3 has been associated with chronic inflammation, which drives the transformation of healthy to cancerous cells [23,24,25,26,27,28,29,30]. Of note, persistent activation of STAT3 has been observed in various types of solid (lung, liver, prostate, breast, head and neck, and gastric) and hematological (leukemia, lymphoma, multiple myeloma) malignancies [31,32,33,34,35]. Given the relevance of STAT3 signaling in oncogenesis, abrogation of the STAT3 signaling cascade may be useful to counteract diverse malignancies.

In an attempt to identify new STAT3 signaling inhibitors, we performed high-throughput virtual screening (HTVS) of a library of small molecules using a cheminformatics platform and identified 2-Amino-6-[2-(Cyclopropylmethoxy)-6-Hydroxyphenyl]-4-Piperidin-4-yl Nicotinonitrile (ACHP) as the lead inhibitor of STAT3. We further tested a predicted lead compound against lung cancer cells for possible STAT3 signaling inhibitory activity and it was found to have pronounced inhibition of the signaling cascade.

## 2. Materials and Methods

### 2.1. Reagents

**Figure 1:**
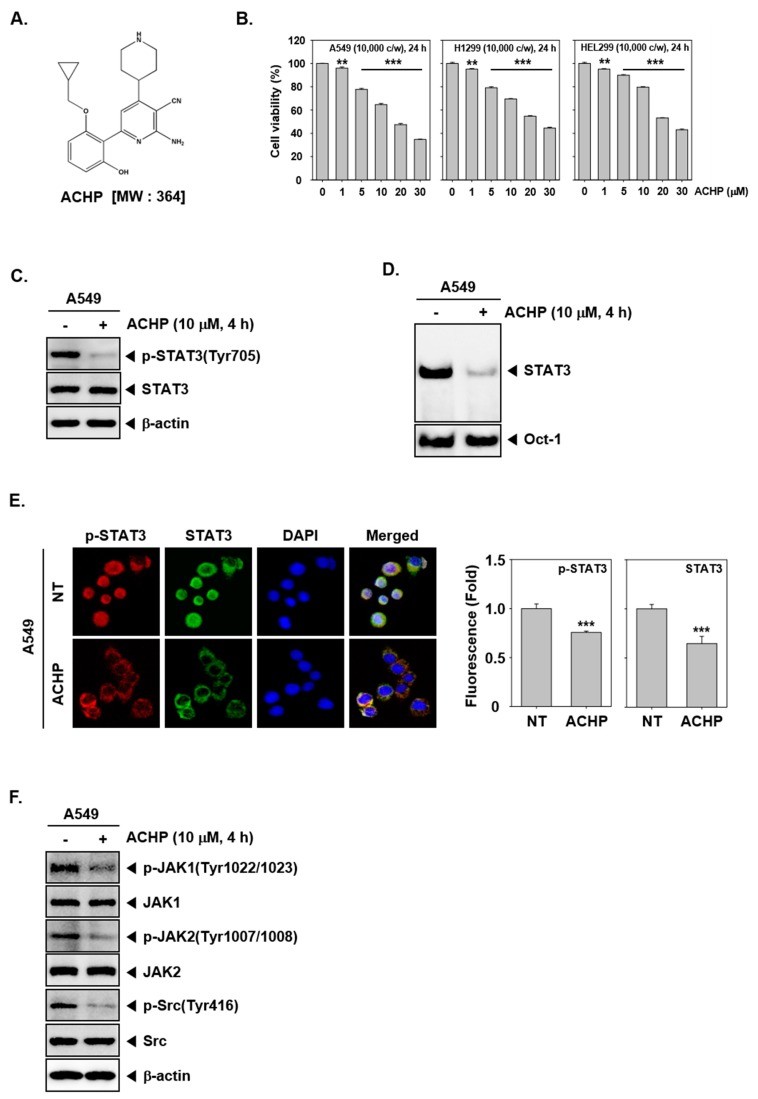
ACHP suppresses cell viability and blocks the STAT3 signaling pathway. (**A**) The chemical structure of ACHP. (**B**) A549, H1299, and HEL 299 cells were treated with various concentrations of ACHP for 24 h, and cell viability was determined by MTT assay. The results shown are representative of three independent experiments. ** *p* < 0.01, *** *p* < 0.001. (**C**) A549 cells were treated with 10 µM of ACHP for 4 h. Thereafter, equal amounts of lysates were analyzed by western blot analysis using antibodies against p-STAT3(Tyr705) and STAT3. The same blots were stripped and reprobed with β-actin antibody to verify equal protein loading. −: Non-treatment, +: ACHP treatment. (**D**) A549 cells were treated with 10 µM of ACHP for 4 h and then tested for DNA binding to STAT3 by electrophoretic mobility shift assay (EMSA). (**E**) A549 cells were treated as described above in panel C and then analyzed for intracellular distribution by immunocytochemistry. The results shown are representative of three independent experiments. *** *p* < 0.001. Quantitative analysis of the fluorescence intensity of p-STAT3 and STAT3 were performed. The merged image indicates the overlapping of p-STAT3/STAT3/DAPI images. The results shown are representative of three independent experiments. *** *p* < 0.001. (**F**) A549 cells were treated as described above in panel C, and western blot was performed using various antibodies. −: Non-treatment, +: ACHP treatment.

ACHP, as shown in Figure 1A, was purchased from TOCRIS Bioscience (Ellisville, MO, USA). The stock solution of ACHP (1 mM) was prepared in dimethyl sulfoxide, stored at −80 °C, and diluted in cell culture medium for use. RPMI1640, DMEM (Dulbecco Modified Eagle Medium)/low, MEM media, fetal bovine serum (FBS), and antibiotic-antimycotic mixture were obtained from Thermo Scientific HyClone (Waltham, MA, USA). A LightShift^®^ Chemiluminescent EMSA kit was obtained from Thermo Fisher Scientific Inc. (Waltham, MA, USA). 5′-biotinylated STAT3 was obtained from Bioneer Corporation (Daejeon, Korea). IL-6 was purchased from R&D Systems (Minneapolis, MN, USA). A FITC Annexin V Apoptosis Detection Kit I was purchased from BD Biosciences (San Diego, CA, USA). TUNEL enzyme and TUNEL label were purchased from Roche (Basel, Switzerland). Green-fluorescent Alexa Fluor 488 anti-Goat IgG1 and red-fluorescent Alexa Fluor 594 anti-Rabbit IgG1 antibodies were purchased from Invitrogen (Carlsbad, CA, USA). Antibodies against p-STAT3(Tyr705), STAT3, β-actin, p-Src (Tyr416), PARP, caspase-3, survivin, Bcl-2, Bcl-xl, HRP-conjugated goat anti-rabbit, and anti-mouse antibodies were purchased from Santa Cruz Biotechnology (Santa Cruz, CA, USA). Antibodies against p-JAK1 (Tyr1022/1023), JAK1, p-JAK2 (Tyr1007/1008), JAK2, cyclin D1, and cleaved caspase-3 were obtained from Cell Signaling Technology (Beverly, MA). The nitrocellulose membrane was obtained from Pall Corporation (Ann Arbor, MI, USA). Chemiluminescent substrate (ECL) was purchased from GE Healthcare (Waukesha, WI, USA). The oligonucleotide sequences used for EMSA are 5′-biotinylated STAT3 (5′-GATCCTTCTGGGAATTCCTAGATC-3′ and 5′-GATCTAGGAATTCCCAGAAGGATC-3′) in complex with nuclear protein and Oct-1 (5′-TTCTAGTGATTTGCATTCGACA-3′ and 5′-TGTCGAATGCAAATCACTAGAA-3′).

### 2.2. Cell Lines and Culture Conditions

Human lung cancer cell lines A549, H1299, and human embryo lung cell lines HEL 299 were purchased from the American Type Culture Collection (Manassas, VA, USA). A549 cells were cultured in DMEM/low medium, H1299 cells in RPMI1640 medium, and HEL 299 cells in MEM medium. All cells were cultured in medium containing 10% fetal bovine serum (FBS) and 1% penicillin-streptomycin (P/S) maintained at 37 °C in a 5% CO_2_ atmosphere. At ~70–90% confluence, the cells were subcultured using 0.05% trypsin/EDTA.

### 2.3. High-Throughput Virtual Screening (HTVS) of Small Molecules Targeting STAT3

The MOLPRINT-2D based cheminformatics tool was used to identify the STAT3 targeting of small molecules as reported earlier [36]. In brief, the bioactivity data of ChEMBL was used, where the cut-off values (IC_50_/EC_50_/Ki/Kd) less than or equal to 10 µM were considered as active and the greater than 10 mM as inactive compounds. MOLPRINT 2D descriptors were obtained for all the datasets using reported protocols [37,38]. Using the Naïve Bayes classifier, the trained datasets were queried with the ZINC database molecules, comprising about 7300 compounds, to obtain the ranked compounds.

### 2.4. Cell Viability Assay

A cell viability assay was performed to evaluate the effect of ACHP on the NSCLC cells as described earlier [39,40,41]. Cells were seeded at a density of 5 × 10^3^ cells per well in 96-well plates and were incubated at 37 °C in 5% CO_2_ overnight to induce cell adherence. Cells were treated with different concentrations of ACHP for 24 h. For the MTT assay, thiazolyl blue tetrazolium bromide solution (2 mg/mL) was added and this mixture was incubated for 2 h. After this, lysis buffer (20% SDS and 50% dimethylformamide) was added to the cells. The cells were incubated overnight at 37 °C, and the absorbance was then measured at 570 nm using a Varioskan LUX Multimode Microplate Reader (Thermo Fisher Scientific).

### 2.5. Preparation of Whole Cell Lysates

For the detection of expression of proteins, ACHP-treated whole-cell lysates were prepared as reported previously [42,43] using a lysis buffer [Tris (20 mM, pH 7.4), NaCl (250 mM), EDTA (2 mM, pH 8.0), Triton X-100 (0.1%), aprotinin (0.01 mg/mL), leupeptin (0.005 mg/mL), phenylmethane sulfonyl fluoride (0.4 mM), and NaVO_4_ (4 mM)]. The lysates were centrifuged at 13,000 rpm for 15 min to remove insoluble material.

### 2.6. Western Blot Analysis

The protein concentration was estimated in cell lysates and equal concentrations of proteins were resolved on 8–15% sodium dodecyl sulfate-polyacrylamide gel electrophoresis followed by their transfer to a nitrocellulose membrane as reported earlier [44,45,46]. The membranes were treated with 5% skim milk and incubated with the desired antibodies at 4 °C overnight. The next day, membranes were washed in an appropriate buffer and probed with HRP-conjugated secondary antibody for 2 h, followed by their examination using chemiluminescent substrate.

### 2.7. Electrophoretic Mobility Shift Assay (EMSA)

EMSA was performed to analyze the interaction of STAT3–DNA as described previously [47,48]. Briefly, cells were subjected to ACHP treatment (10 µM for 4 h) and the nuclear extract was prepared. 5′-biotinylated STAT3 oligonucleotide in complex with nuclear protein and Oct-1 was used for the loading control. The protein–oligonucleotide complex was subjected to PAGE and blotted to a nylon membrane, followed by cross-linkage with UV. Lastly, the mobility of proteins was examined using a LightShift^®^ Chemiluminescent EMSA kit (Waltham, MA, USA) [47].

### 2.8. Immunocytochemistry for the Distribution of STAT3

The distribution of phosphorylated STAT3 in the cells were analyzed as described earlier [49]. In brief, cells were subjected to ACHP treatment (10 µM for 4 h), followed by fixing using paraformaldehyde (4%) for 20 min. Thereafter, cells were treated with 0.2% Triton X-100 in phosphate-buffered saline for permeabilization, followed by blocking with 5% bovine serum albumin for 1 h. Then, the preparation was incubated overnight at 4 °C with a rabbit polyclonal anti-human STAT3 antibody (dilution, 1:100). The next day, slides were subjected to washing and incubation with Alexa Fluor 594 (dilution, 1:1000) anti-Rabbit IgG1 for 1 h at room temperature in the dark. In the next step, DAPI (5 µg/mL) was used for counterstaining the nuclei. The slides were mounted and analyzed under an Olympus FluoView FV1000 confocal microscope (Tokyo, Japan).

### 2.9. Monitoring of Cell Growth with the RTCA DP Instrument

The growth of A549 and H1299 cells were constantly assessed for 48 h using the xCELLigence RTCA DP Instrument (Roche Diagnostics GmbH, Mannheim, Germany). The reading was taken using 100 µL cell culture medium per well and the final volume in the well was made up to 200 µL using the cell culture medium bearing 5 × 10^3^ cells/well. The impedance was measured continuously in 15 min intervals. Cell index (CI) values were normalized to the time point of 10 µM of ACHP treatment.

### 2.10. siRNA Transfection

A549 cells were seeded and transiently transfected with STAT3 or scrambled siRNAs using a transfection reagent (Intron Biotechnology, Seoul, Korea). At 24 h post-transfection, the cells were treated with 10 μM of ACHP for 24 h or 36 h and then an MTT and TUNEL assay was performed.

### 2.11. Annexin V Assay

The apoptosis-inducing effect of ACHP on A549 and H1299 cells was evaluated using an Annexin assay as described previously [46,50]. The A549 and H1299 cells were exposed to ACHP (10 µM) for indicated time points and collected using trypsin (1%) in phosphate-buffered saline, followed by giving a single wash with cold phosphate-buffered saline. The cell pellet was collected and suspended in binding buffer (1×). Thereafter, FITC Annexin V (5 µL) and propidium iodide (5 µL) were added and incubated in the dark at room temperature for 15 min to stain the cells. Stained samples were analyzed by a BD Accuri C6 plus flow cytometer (BD Biosciences, San Diego, CA, USA). The interpretation of the data was performed using BD Accuri C6 plus software (version 1.0.23.1).

### 2.12. TUNEL Assay

The apoptosis-inducing effect of ACHP was further evaluated using a Roche Diagnosis TUNEL (terminal transferase-mediated dUTP-fluorescein nick end labelling) assay kit as reported earlier [49]. A549 and H1299 cells were exposed to ACHP (10 µM) for indicated time points and washed with cold phosphate-buffered saline, followed by fixing using paraformaldehyde (4%) for 30 min and washing twice with phosphate-buffered saline. Thereafter, cells were treated with 0.1% Triton X-100 and 0.1% sodium citrate for permeabilization for 20 min at 4 °C, followed by washing with cold phosphate-buffered saline. The preparation was treated with a TUNEL enzyme and TUNEL label mixture for 1 h at 37 °C in the dark, followed by washing with phosphate-buffered saline and analyzed using BD Accuri C6 plus software (version 1.0.23.1).

### 2.13. In Silico Interaction Analysis

Discovery Studio 2.5 software from Accelrys was used for docking and visualization of the results as described earlier [51,52]. Initially, we obtained the crystal structure of the STAT3 homodimer bound to DNA (PDB ID: 1BG1) [53], cleaned, minimized the energy, and identified the spatial region of STAT3. The CHARMM force field function was used to calculate the energy calculations. The three-dimensional structure of ligands (ACHP and HAB) were produced and docked towards the SH2 domain of STAT3 using the LIGANDFIT protocol. The interaction map and binding position of ACHP and HAB were evaluated using the interaction score function in the Ligand Fit module of Discovery Studio as reported previously [54].

### 2.14. Statistical Analysis

The Student’s *t*-test and one-way analysis of variance (ANOVA) tests were used for statistical analysis and comparisons between groups. *p* < 0.05 was considered significant. Data are expressed as the mean ± SD and vertical error bars denote SD.

## 3. Results

### 3.1. In Silico Approach for the Identification of Ligands Targeting STAT3

The cheminformatics-based MOLPRINT-2D program was used to identify the lead compounds that target STAT3. For this, active and inactive datasets for a STAT3 model were built and queried with the ZINC database as described earlier [55]. The ranked compounds are provided as a supplementary file and the structure of the top four ranked compounds that target STAT3 are provided as a supplementary figure. Among the queried compounds, ACHP was ranked first and therefore, it was procured from the private firm and used for evaluation of its STAT3 inhibitory activity.

### 3.2. ACHP Reduces NSCLC Cell Viability

To examine the effect of ACHP on the growth of human lung cancer cells and human embryonic lung fibroblasts, the growth inhibitory potential was determined in A549, H1299, and HEL299 cells. We found the concentration-dependent reduction in cell viability on treatment with ACHP for 24 h. The A549 cell viability values of ACHP were 95%, 77%, 64%, 47%, and 34%, and the viability values of H1299 cells were 95%, 79%, 69%, 54%, and 44%, at the concentration of 1, 5, 10, 20, and 30 µM, respectively.

### 3.3. ACHP Inhibits Constitutively Active STAT3 in NSCLC

Persistent activation of STAT3 contributes to uncontrolled cell proliferation, angiogenesis, apoptotic resistance, and prosurvival effect in cancer cells [26]. Therefore, we evaluated whether ACHP can suppress the constitutive activation of STAT3. In A549 cells, ACHP treatment reduced the STAT3 phosphorylation at Tyr705 without affecting the total STAT3 protein expression. These results clearly suggest that ACHP significantly downregulates the constitutive phosphorylation of STAT3 in lung cancer cells, as shown in Figure 1C. ACHP also clearly reduced the constitutive STAT3 DNA-binding activity, as shown in Figure 1D.

### 3.4. ACHP Blocks the Nuclear Localization of STAT3 in A549 Cells

Activated STAT3 dimers can enter the nucleus to transcribe its target genes [56,57]. To confirm that nuclear-translocated STAT3 is inhibited by ACHP, we next analyzed the distribution of phospho-STAT3 in the nucleus and cytoplasm using fluorescent-labeled antibodies. As shown in Figure 1F, ACHP suppressed the nuclear translocation of STAT3 in A549 cells, as shown in Figure 1E.

### 3.5. ACHP Represses Constitutive JAK1, JAK2, and Src Activation

STAT3 is phosphorylated by the upstream tyrosine kinases, such as JAK1, JAK2, and Src [58,59]. We next investigated the effect of ACHP on the activation of JAK1, JAK2, and Src in A549 cells. ACHP treatment resulted in the downregulation of JAK1, JAK2, and Src phosphorylation, whereas ACHP did not affect these total forms, as shown in Figure 1F.

### 3.6. ACHP Inhibits IL-6-Induced Activation of STAT3 and Upstream Kinases

**Figure 2:**
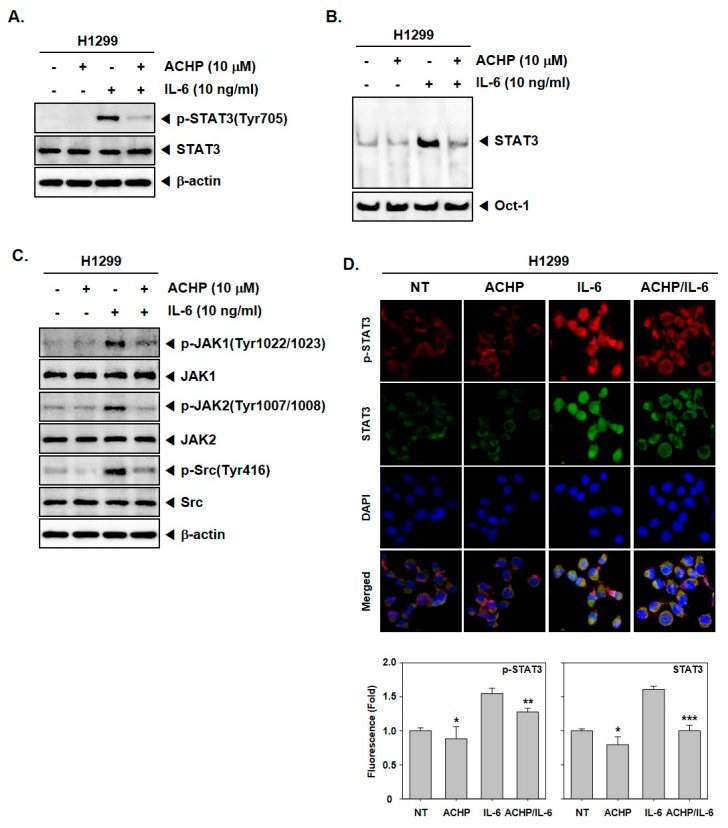
ACHP inhibits IL-6-induced STAT3 activation. (**A**) H1299 cells were treated with 10 µM of ACHP for 4 h and then stimulated with IL-6 (10 ng/mL) for 15 min. Thereafter, equal amounts of lysates were analyzed by western blot analysis using antibodies against p-STAT3(Tyr705) and STAT3. The same blots were stripped and reprobed with β-actin antibody to verify equal protein loading. (**B**) H1299 cells were treated with 10 µM of ACHP for 4 h and then stimulated with IL-6 (10 ng/mL) for 15 min and then tested for DNA binding to STAT3 by EMSA. (**C**) H1299 cells were treated as described above in panel A, and western blot was performed using various antibodies. (**D**) H1299 cells were treated as described above in panel A and then analyzed for intracellular distribution by immunocytochemistry. The results shown are representative of three independent experiments. *** *p* < 0.001. Quantitative analysis of the fluorescence intensity of STAT3 was performed. The merged image indicates the overlapping of p-STAT3/STAT3/DAPI images. * *p* < 0.05, ** *p* < 0.01, *** *p* < 0.001. −: Non-treatment, +: ACHP treatment.

We further investigated the effect of ACHP on IL-6-driven activation of STAT3 in H1299 cells. To analyze this, H1299 cells were treated with ACHP (10 μM) for 6 h followed by stimulation with IL-6 for 15 min. We found that ACHP completely reverted the IL-6-driven phosphorylation of STAT3 in the tested cells, as shown in Figure 2A. The effect of ACHP on IL-6 driven STAT3 activation was further justified using an electrophoretic mobility shift assay (EMSA) and ACHP downregulated IL-6-induced STAT3 activation, as shown in Figure 2B. In addition, JAK1, JAK2, and Src, the kinases of the upstream signaling pathway of STAT3, were maximally activated at 15 min after exposure of H1299 cells to IL-6, whereas it was inhibited by ACHP, as shown in Figure 2C. Interestingly, as shown in Figure 2D, ACHP blocked translocation of IL-6-induced STAT3 to the nucleus in H1299 cells.

### 3.7. ACHP Blocks the Proliferation Activity of NSCLC.

**Figure 3:**
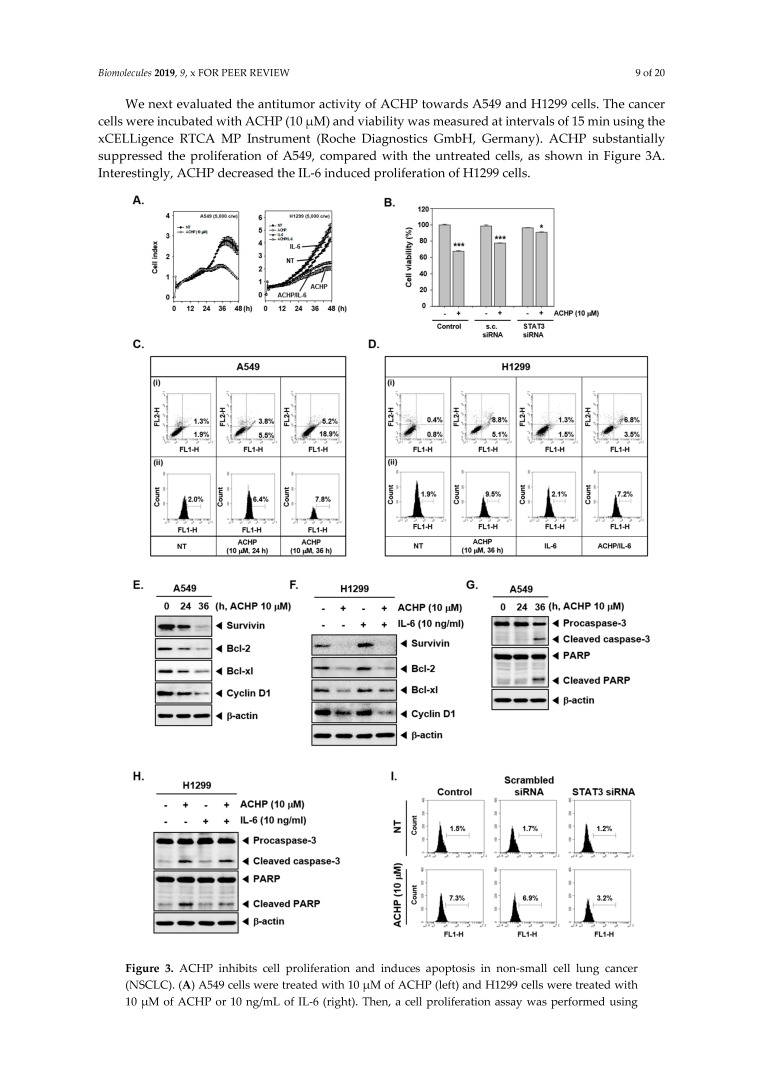
ACHP inhibits cell proliferation and induces apoptosis in non-small cell lung cancer (NSCLC). (**A**) A549 cells were treated with 10 µM of ACHP (left) and H1299 cells were treated with 10 µM of ACHP or 10 ng/mL of IL-6 (right). Then, a cell proliferation assay was performed using Roche xCELLigence Real-Time Cell Analysis (RTCA). (**B**) A549 cells were transfected with either scrambled siRNA or STAT3-specific siRNA (50 nM). After 24 h, the cells were treated with 10 μM of ACHP for 24 h, and cell viability was determined by MTT assay. The results shown are representative of three independent experiments. *** *p* < 0.001. (**C**) A549 cells were treated with 10 µM of ACHP for 24 h and 36 h. (i) The cells were incubated with a FITC conjugated Annexin V, then examined for an early apoptotic effect with flow cytometry. FL1-H: Annexin V-FITC, FL2-H: PI. (ii) The cells were fixed and incubated with a TUNEL reaction solution, and then examined via flow cytometry. (**D**) H1299 cells were treated with 10 µM of ACHP or 10 ng/mL of IL-6 for 36 h. (i) The cells were incubated with a FITC conjugated Annexin V, then examined for an early apoptotic effect with flow cytometry. FL1-H: Annexin V-FITC, FL2-H: PI. (ii) The cells were fixed and incubated with a TUNEL reaction solution, and then examined via flow cytometry. (**E**) A549 cells were treated with 10 µM of ACHP for 24 h and 36 h, then western blotting was performed using various antibodies. (**F**) H1299 cells were treated with 10 µM of ACHP or 10 ng/mL of IL-6 for 36 h, then western blotting was performed using various antibodies. (**G**) A549 cells were treated as described above in panel E, and a western blot was done. (**H**) H1299 cells were treated as described above in panel F, and a western blot was done. Data are expressed as the mean ± SD and vertical error bars denote SD. (**I**) A549 cells were transfected with either scrambled siRNA or STAT3-specific siRNA (50 nM). After 24 h, the cells were treated with 10 μM of ACHP for 36 h and incubated with TUNEL reaction solution, and then examined via flow cytometry. * *p* < 0.05, ** *p* < 0.01, *** *p* < 0.001. −: Non-treatment, +: ACHP treatment.

We next evaluated the antitumor activity of ACHP towards A549 and H1299 cells. The cancer cells were incubated with ACHP (10 µM) and viability was measured at intervals of 15 min using the xCELLigence RTCA MP Instrument (Roche Diagnostics GmbH, Germany). ACHP substantially suppressed the proliferation of A549, compared with the untreated cells, as shown in Figure 3A. Interestingly, ACHP decreased the IL-6 induced proliferation of H1299 cells.

### 3.8. Transfection with STAT3 siRNA Blocks ACHP-Induced Cytotoxicity

We determined whether the knockdown of STAT3 using siRNA could significantly block the increase in ACHP induced cytotoxicity in A549 cells. In A549 cells transfected with scrambled siRNA, ACHP treatment significantly reduced cell viability, but not in the cells transfected with STAT3 siRNA, thereby establishing STAT3 as a pivotal target affected by ACHP, as shown in Figure 3B.

### 3.9. ACHP Induces Apoptosis and Decreases the Expression of Tumorigenic Proteins

We further examined the apoptosis induction potential of ACHP in A549 and H1299 cells using annexin V and TUNEL assays. Annexin V assay results presented that ACHP enhanced the apoptotic population to 24.1% (5.2% + 18.9%) in A549 cells at the dose of 10 µM for 36 h compared with the untreated control cells, as shown in Figure 3C-i. The percentage of apoptotic cells in H1299 cells reached 13.9% (8.8% + 5.1%) by ACHP and decreased to 10.3% (6.8% + 3.5%) in the combined treatment of IL-6, as shown in Figure 3D-i. The results of the TUNEL assay also indicated that the apoptotic population was increased from 2.0% to 6.4% at 24 h and 7.8% at 36 h after the administration of the drug in A549 cells, as shown in Figure 3C-ii. In H1299 cells, the apoptotic population increased to 9.5% by ACHP, whereas it decreased to 7.2% by IL-6, as shown in Figure 3D-ii.

We also evaluated the effect of ACHP on the expression of antiapoptotic proteins in NSCLC. We observed that the ACHP decreased the levels of prosurvival proteins, such as survivin, Bcl-2, Bcl-xl, and cell cycle regulator, cyclin D1, in a time-dependent manner in A549 cells, as shown in Figure 3D. On the other hand, IL-6 treatment induced the expression of survivin, Bcl-2, Bcl-xl, and cyclin D1, whereas ACHP significantly abrogated their expression, as shown in Figure 3E.

### 3.10. ACHP Triggers the Activation of Procaspase-3 and Induces PARP Cleavage

We next examined whether the blocking of STAT3 activation by ACHP leads to caspase-3 mediated apoptosis in NSCLC. We observed the cleavage of full-length procaspase-3 with the corresponding increase in cleaved fragments on treatment with ACHP at 10 µM for 36 h, as shown in Figure 3F. In A549 and H1299 cells treated with ACHP, there were increased cleavage products of PARP and caspase-3, as shown in Figure 3G, whereas they were marginally affected by IL-6, as shown in Figure 3H.

### 3.11. Knockdown of STAT3 Reverses the Apoptotic Effect of ACHP

We determined whether the knockdown of STAT3 using siRNA could significantly block the increase in ACHP-induced apoptosis in A549 cells. As shown in Figure 3I, the ACHP treatment alone resulted in substantial apoptosis (7.3%), whereas only 3.2% of the cells in the STAT3 knockdown group were found to be apoptotic. These findings indicate that STAT3 could be one of the major molecular targets that can be affected by ACHP.

### 3.12. ACHP Interacts with the SH2 Domain of STAT3 In Silico

**Figure 4:**
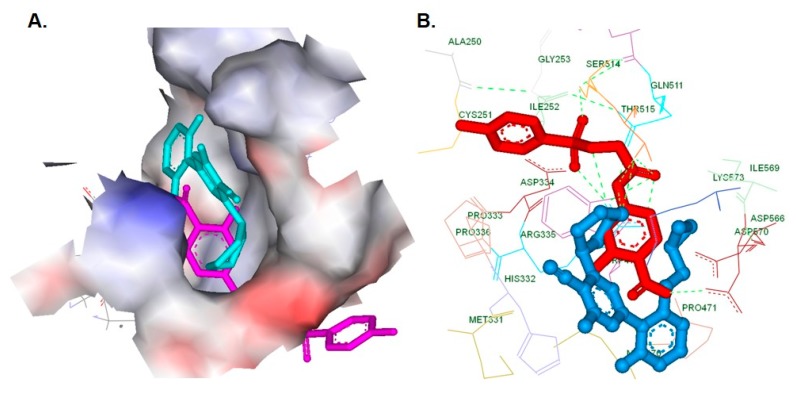
In silico interaction analysis between the SH2 domain of STAT3 and the ligands. (**A**) Surface view of the ACHP/HAB bound SH2 domain of STAT3. (**B**) Interaction map and hydrogen bonding (dotted line) pattern of the SH2 domain of STAT3 with ACHP and HAB.

We performed in silico analysis to determine the possible molecular interactions between the ACHP and SH2 domain of STAT3. ACHP and 2-hydroxy-4-(2-(tosyloxy) acetamido)benzoic acid (HAB), a known STAT3 inhibitor, were docked with the crystal structure of a STAT3 monomer that bound to a DNA oligomer (PDB ID: 1BG1) using the CDOCKER protocol of Accelrys discovery studio 2.5 version, as shown in Figure 4A. Molecular docking analysis gave the ligand-protein binding-energy value, interactions, and also the putative bound conformations of them. The negative CDOCKER energy score for ACHP and HAB towards the SH2 domain of STAT3 and ACHP presented a relatively higher score than its counterpart. The ACHP and HAB shared a similar binding pattern and interacted with Pro471, Ile467, Met470, Trp474, Arg335, Asp570, Ile569, and Asp566, as shown in Figure 4B. A hydrogen bond was established between Thr515 and the amino group of ACHP, and amide of HAB. Additionally, the tosyloxy group and carboxylic acid of HAB established a hydrogen bond with Arg335 and Asp570, respectively. These results suggested that ACHP may have physical interaction with STAT3 protein to induce its inhibitory activity.

## 4. Discussion

The therapeutic efficacy of the blockade of the STAT3 signaling pathway in cancers has been extensively studied, and a number of STAT3 inhibitors have been developed [60,61,62,63]. In the present investigation, we determined the cytotoxic effect of ACHP on the panel of NSCLC cells and found that ACHP possesses a good cytotoxic effect on the tested cancer cell lines. ACHP was found to mediate its cytotoxicity by abrogating the STAT3 signaling pathway. ACHP is a piperidinyl nicotinonitrile derivative, which was initially identified as a selective inhibitor of IKK-β with good aqueous solubility, cell permeability, and oral bioavailability profile in mice and rats [64,65]. In addition, ACHP has also been demonstrated to show inhibitory action towards other kinases, such as IKK-α (IC_50_: 250 nM), IKK3, Syk, and MKK4 (IC_50_ > 20 µM) [66]. Previous studies also suggest that ACHP exhibited cytotoxicity in adult T-cell leukemia and multiple myeloma cells by interfering with NF-κB signaling [65,67]. The activation of NF-κB, in addition to controlling tumorigenesis [68,69,70,71,72], plays a key role in the induction of fibrosis and ACHP displayed strong antifibrotic effects by suppressing the TGFβ1-induced differentiation of fibroblasts into myofibroblasts and collagen synthesis [73]. Recently, ACHP has been reported to block NF-κB signaling in mouse and human keratinocytes and inhibit multiple sources of cutaneous inflammation in mouse skin [74]. Besides, persistent activity of NF-κB and STAT3 has been linked with oncogenesis [75,76], and abrogation of either of these pathways may not lead to significant cytotoxicity [77]. In addition, a small molecule inhibitor (JSI-124 or cucurbitacin I) of STAT3 signaling was reported to activate the NF-κB pathway [78]. Therefore, it may be an effective strategy to have a dual inhibitor of STAT3 and NF-κB pathways to induce potent cytotoxicity [79]. Similarly, 2-[(aminocarbonyl)amino]-5-(4-fluorophenyl)-3-thiophenecarboxamide (TPCA-1) is a synthetic small molecule that has been reported as an ATP-competitive selective inhibitor of IKK2 [80], and subsequent discoveries presented TPCA-1 as a direct dual inhibitor of STAT3 and NF-κB that effectively regresses mutant EGFR-associated human NSCLC [77]. Herein, we performed HTVS of small molecules bearing various scaffolds against STAT3 inhibition and identified ACHP as the lead hit. The predicted target was experimentally validated in NSCLC cellular models.

Overexpression of STAT3 has been reported to potentiate growth, survival, and chemo- and radio-resistance of NSCLC [81,82] and human squamous cell carcinoma cells [83,84]. A significant correlation was found between STAT3 protein expression and tumor differentiation, clinical stage, and lymph node metastasis of NSCLC patients [85]. Notably, the five-year overall survival rate of patients with low STAT3 expression was significantly higher than that of patients with high STAT3 expression indicating the role of STAT3 as a prognostic marker [85]. In addition, several studies have demonstrated the persistent phosphorylation of STAT3 in 22–65% of NSCLC [81], and the deregulation of STAT3 has been associated with malignant transformation [86,87]. The phosphorylation of Tyr705 is a critical event in regulating the transcriptional activity of STAT3, and mitigation of phosphorylation can result in a decline in the STAT3 nuclear pool [88]. Therefore, blocking of nuclear translocation of STAT3 by inhibiting its phosphorylation could be a therapeutic approach. In addition, earlier studies have also demonstrated that STAT3 is present in phosphorylated, as well as in unphosphorylated form in the endosomes. This also suggested that, in addition to the signal transducer role, the membrane-associated cytoplasmic STAT3 may also have a role in STAT3 metabolism [89].

In our study, ACHP was found to significantly inhibit the phosphorylation of STAT3 at Tyr705, which was evidently demonstrated using multiple approaches, and the molecular mechanism by which the ACHP inhibits STAT3 signaling in NSCLC cells has been studied. In addition to the downregulation of phosphorylation, we also noticed the significant deprivation of nuclear STAT3 levels and reduction in DNA binding activity, which is evidence for the decline in the transcription of STAT3 driven genes. Furthermore, ACHP was found to reduce the cell viability of the tested cell lines and we speculated the cytotoxic effect is due to inhibition of STAT3 signaling. To verify this, we knocked down STAT3 using siRNA and tested the effect of ACHP on cell viability of A549 cells. We observed minimal effect on the viability of STAT3-depleted A549 cells, thus indicating the absence of off-target effects.

STAT3 protein can be positively modulated by phosphorylated upstream protein tyrosine kinases, such as Src (Tyr416) and JAK (JAK1: Tyr1022/1023; and JAK2: Tyr1007/1008) [90]. We observed a substantial decrease in the phosphorylation of Src and JAKs. H1299 cells lack the constitutive activity of STAT3 signaling, and we observed the phosphorylation of Src, JAK1, and JAK2 on treatment with IL-6. It is noteworthy that ACHP has been previously demonstrated to exhibit inhibitory activity towards serine/threonine kinases (IKK-α/β), as well as tyrosine kinase (Syk) [66]. In the present effort, we have explored another cellular target kinase of ACHP. Furthermore, ACHP treatment suppressed the IL-6 induced activation of these cascades of proteins in H1299 cells. Activation of executioner caspase (caspase 3/7) is the major biochemical event associated with the cells committed to apoptosis [91], and the activated caspase-3 cleaves PARP to induce apoptosis [54,92].

We noticed that ACHP induced the activation of caspase-3 and cleavage of PARP. Evidently, we also observed the negative modulation in the expression of apoptosis modulators such as Bcl-2, Bcl-xl, and cyclin D1. In addition to its role in the activation of oncogenic gene expression, STAT3 has also been demonstrated to repress the expression of tumor suppressor genes to encourage the survival of cancer cells [93,94,95]. In contrast, some of the studies have highlighted STAT3 as a tumor suppressor protein. In one of the early studies, simultaneous shRNA-mediated knockdown of PTEN and deletion of STAT3 showed substantial increase in in vitro proliferation cells and tumor formation in SCID (Severe combined immunodeficient) mice in astrocytes. In parallel, knockdown of PTEN alone with normal STAT3 expression displayed significantly reduced tumorigenic potential, indicating that STAT3 serves as a tumor suppressor in the absence of PTEN [96]. In similar studies, the tumor suppressor functions of STAT3 were found to have relevance with Ras and p19^ARF^ protein expression [97].

In 2018, Caetano et al. developed a lung epithelial-specific K-ras mutant/STAT3 conditional knockout mouse model, and deletion of epithelial STAT3 resulted in sex-associated discrepancies in which K-ras mutant tumors were decreased in female K-ras mutant/STAT3 conditional knockout, whereas tumor burdens were increased in males [98]. These reports spread light on the multifaceted role of STAT3 in oncogenic and tumor suppressor effects. However, our study highlights the constitutive activation of STAT3 in lung cancer cells and ACHP induced cell death via blocking oncogenic STAT3 signaling. We have previously reported several natural compounds that induce their inhibitory activity towards upstream kinases (JAK/Src) in cell-based assays and displayed interaction with the SH2 domain of STAT3 in computational studies. We obtained similar results in the present study and the exact mechanisms through which ACHP can interrupt STAT3 signaling either through interaction with its SH2 domain or attenuation of phosphorylation of upstream kinases requires further investigations. With these shreds of evidence, we have conclusively reported that STAT3 is the additional signaling cascade impeded by ACHP. In summary, our study shows the feasibility of inhibiting a constitutively active STAT3 signaling pathway in NSCLC cells.

## Supplementary Materials

The Supplementary Materials are available online at https://www.mdpi.com/2218-273X/9/12/875/s1.
