# Supplementary material for: The IκB Kinase Inhibitor ACHP Targets the STAT3 Signaling Pathway in Human Non-Small Cell Lung Carcinoma Cells
Source: Biomolecules. 2019 Dec 13;9(12):875. doi: 10.3390/biom9120875 (PMC6995615; doi:10.3390/biom9120875)
Supplement: Supplementary file 1 [file biomolecules-09-00875-s001.pdf]

EMSA

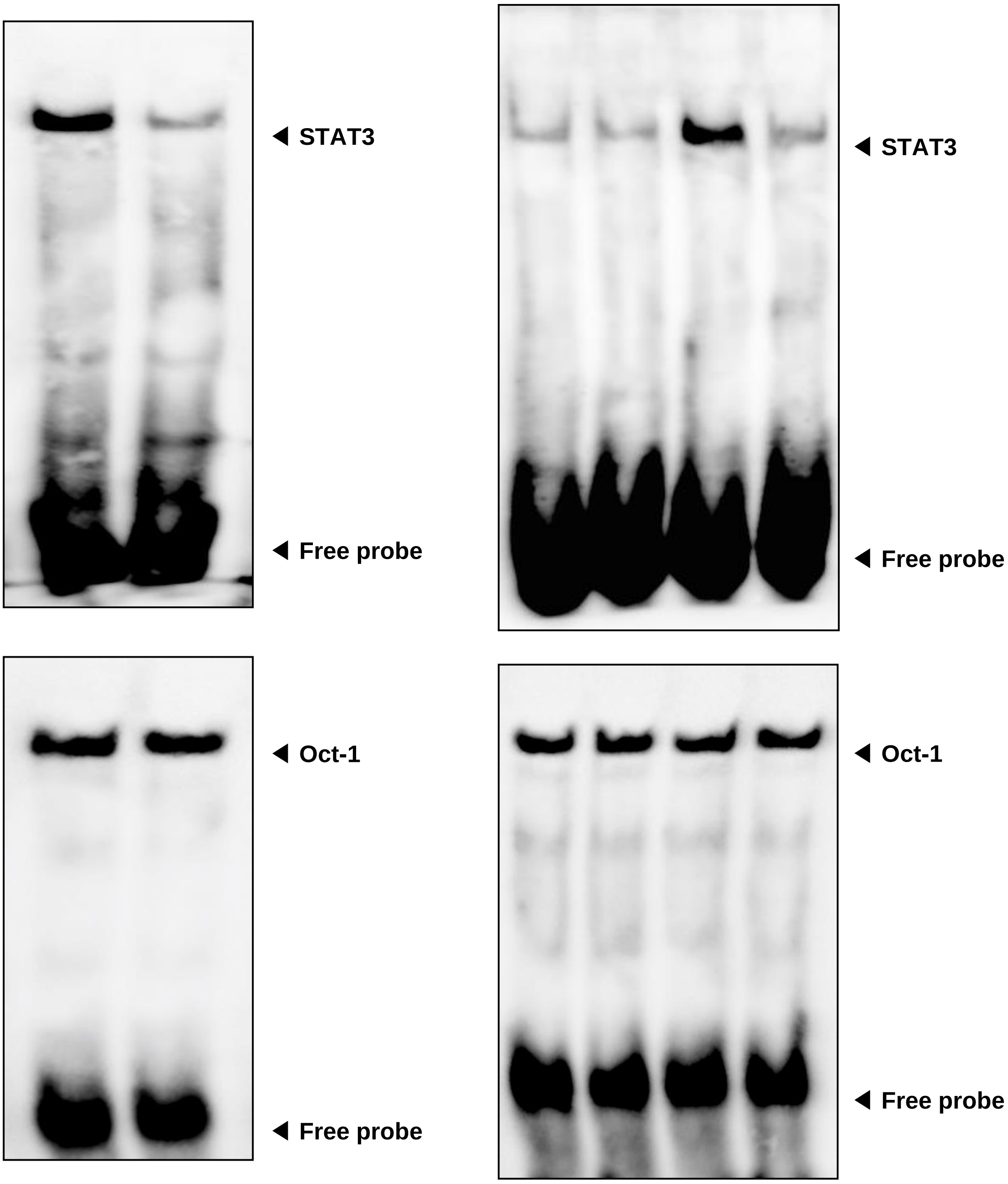
STAT3
Free probe
STAT3
Free probe
Oct-1
Free probe
Oct-1
Free probe

Top four ranked compounds identified to target STAT3

ZINC6718453

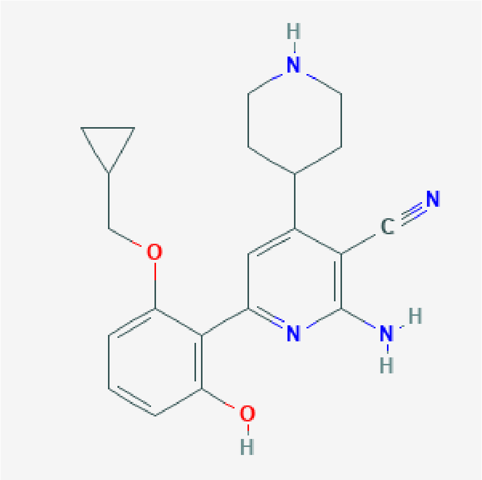

ZINC08441103

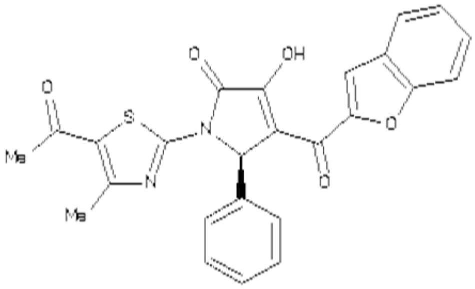

ZINC08440465

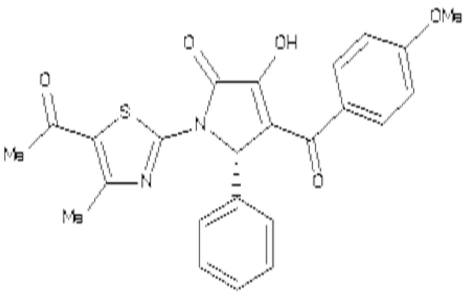

ZINC00702936

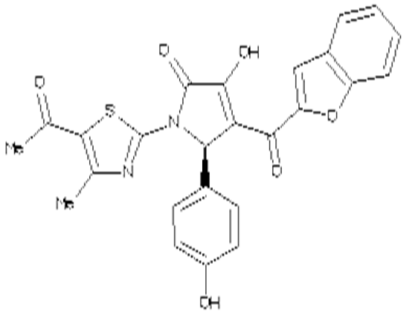

HTVS ranking of compounds

database	15	ZINC6718453	364.446868
database	882	ZINC08441103	562.1329845
database	1199	ZINC08440465	562.1329845
database	868	ZINC00702936	562.1329845
database	866	ZINC00702935	562.1329845
database	880	ZINC00702925	562.1329845
database	870	ZINC08441127	562.1329845
database	874	ZINC00702930	562.1329845
database	878	ZINC00702928	562.1329845
database	861	ZINC00702948	562.1329845
database	872	ZINC08441114	562.1329845
database	1205	ZINC00702554	562.1329845
database	876	ZINC00702927	562.1329845
database	859	ZINC00702947	562.1329845
database	5213	ZINC08837018	374.755323
database	985	ZINC00702761	374.755323
database	2236	ZINC00626508	374.755323
database	1464	ZINC00717758	374.755323
database	1001	ZINC05921386	374.755323
database	2147	ZINC01413489	374.755323
database	2263	ZINC01019496	374.755323
database	2039	ZINC01414771	374.755323
database	2325	ZINC00625888	374.755323
database	2283	ZINC08438677	374.755323
database	2116	ZINC01414763	374.755323

database	689	ZINC08441471	374.755323
database	3330	ZINC06156995	374.755323
database	2233	ZINC00626535	374.755323
database	3325	ZINC06177076	374.755323
database	2191	ZINC01413499	374.755323
database	2297	ZINC08438658	374.755323
database	1004	ZINC00702733	374.755323
database	2105	ZINC01413449	374.755323
database	2360	ZINC00625740	374.755323
database	5280	ZINC00836256	374.755323
database	2239	ZINC00626518	374.755323
database	735	ZINC00703050	374.755323
database	703	ZINC08441462	374.755323
database	1731	ZINC08439285	374.755323
database	709	ZINC08441455	374.755323
database	1323	ZINC17160093	374.755323
database	472	ZINC03646241	374.755323
database	820	ZINC08897840	374.755323
database	1321	ZINC17160093	374.755323
database	1314	ZINC00702426	374.755323
database	2194	ZINC01413490	374.755323
database	2293	ZINC08438660	374.755323
database	2122	ZINC01413446	374.755323
database	2376	ZINC08438528	374.755323
database	2301	ZINC08438654	374.755323
database	2372	ZINC08438531	374.755323
database	1466	ZINC00717759	374.755323

database	493	ZINC00703128	374.755323
database	1275	ZINC00702446	374.755323
database	1733	ZINC08439285	374.755323
database	1613	ZINC00717196	374.755323
database	4131	ZINC00985526	374.755323
database	2218	ZINC08438704	374.755323
database	2367	ZINC00625470	374.755323
database	2199	ZINC01413491	374.755323
database	568	ZINC08441630	374.755323
database	982	ZINC00702760	374.755323
database	2087	ZINC01414786	374.755323
database	3971	ZINC08432721	374.755323
database	2093	ZINC01414751	374.755323
database	1085	ZINC00702689	374.755323
database	2320	ZINC00625874	374.755323
database	5545	ZINC08424490	374.755323
database	3249	ZINC00864421	374.755323
database	1368	ZINC08440096	374.755323
database	2155	ZINC01413497	374.755323
database	2112	ZINC01414732	374.755323
database	2362	ZINC08438558	374.755323
database	3758	ZINC08742399	374.755323
database	2252	ZINC06445943	374.755323
database	2224	ZINC00626544	374.755323
database	1027	ZINC00702724	374.755323
database	2254	ZINC06445943	374.755323
database	2035	ZINC01414770	374.755323

database	483	ZINC00703147	374.755323
database	2062	ZINC01413468	374.755323
database	3746	ZINC06144032	374.755323
database	1280	ZINC08440216	374.755323
database	1029	ZINC00702725	374.755323
database	1105	ZINC05918668	374.755323
database	2334	ZINC08438563	374.755323
database	706	ZINC08441459	374.755323
database	2353	ZINC00625729	374.755323
database	2075	ZINC01414784	374.755323
database	800	ZINC00702990	374.755323
database	5275	ZINC00836245	374.755323
database	2143	ZINC01413488	374.755323
database	1092	ZINC00702692	374.755323
database	1031	ZINC00702726	374.755323
database	2188	ZINC01413498	374.755323
database	3297	ZINC08817352	374.755323
database	2323	ZINC00625888	374.755323
database	544	ZINC00703105	374.755323
database	3760	ZINC08742399	374.755323
database	1317	ZINC00702427	374.755323
database	481	ZINC00703149	374.755323
database	1738	ZINC08439281	374.755323
database	2350	ZINC00625722	374.755323
database	3327	ZINC06177076	374.755323
database	1424	ZINC00702307	374.755323
database	2136	ZINC08438771	374.755323

database	2347	ZINC00625759	374.755323
database	1444	ZINC09109830	374.755323
database	2291	ZINC08438660	374.755323
database	5222	ZINC09271506	374.755323
database	2249	ZINC06445928	374.755323
database	2343	ZINC00625755	374.755323
database	733	ZINC00703050	374.755323
database	2097	ZINC01414752	374.755323
database	814	ZINC16956755	374.755323
database	3322	ZINC06177078	374.755323
database	1058	ZINC00702701	374.755323
database	805	ZINC00702988	374.755323
database	1181	ZINC00702588	374.755323
database	2271	ZINC08438685	374.755323
database	2085	ZINC01414786	374.755323
database	764	ZINC00703043	374.755323
database	5278	ZINC00836256	374.755323
database	791	ZINC08441316	374.755323
database	2227	ZINC00626552	374.755323
database	2340	ZINC00625750	374.755323
database	754	ZINC08441400	374.755323
database	1063	ZINC00702695	374.755323
database	2059	ZINC01414774	374.755323
database	793	ZINC08441316	374.755323
database	2565	ZINC00623246	374.755323
database	700	ZINC08441465	374.755323
database	2221	ZINC08438703	374.755323

database	2378	ZINC08438527	374.755323
database	2077	ZINC01414784	374.755323
database	3252	ZINC00864422	374.755323
database	2125	ZINC01413447	374.755323
database	2041	ZINC01414772	374.755323
database	988	ZINC00702762	374.755323
database	2081	ZINC01414785	374.755323
database	1361	ZINC00702370	374.755323
database	2079	ZINC01414785	374.755323
database	3310	ZINC06445233	374.755323
database	2542	ZINC01815197	374.755323
database	2268	ZINC08438686	374.755323
database	1042	ZINC00702713	374.755323
database	2170	ZINC01413501	374.755323
database	798	ZINC00702989	374.755323
database	2045	ZINC01413485	374.755323
database	1179	ZINC00702587	374.755323
database	2049	ZINC01414765	374.755323
database	3686	ZINC08433275	374.755323
database	4456	ZINC05175548	374.755323
database	2215	ZINC08438705	374.755323
database	495	ZINC00703129	374.755323
database	743	ZINC00703052	374.755323
database	2328	ZINC08438566	374.755323
database	2247	ZINC06445928	374.755323
database	1450	ZINC09041905	374.755323
database	1442	ZINC00721238	374.755323

database	2286	ZINC08438676	374.755323
database	1268	ZINC08440218	374.755323
database	2009	ZINC08438781	374.755323
database	3701	ZINC09271700	374.755323
database	1007	ZINC00702734	374.755323
database	1319	ZINC00702427	374.755323
database	3275	ZINC09280114	374.755323
database	2544	ZINC01764019	374.755323
database	1459	ZINC00717892	374.755323
database	1272	ZINC00702446	374.755323
database	2174	ZINC01413494	374.755323
database	2052	ZINC01414766	374.755323
database	1288	ZINC00702437	374.755323
database	3306	ZINC08435579	374.755323
database	757	ZINC08441398	374.755323
database	5546	ZINC08424489	374.755323
database	563	ZINC00703108	374.755323
database	2512	ZINC08437882	374.755323
database	586	ZINC08441623	374.755323
database	2503	ZINC08437892	374.755323
database	1022	ZINC00702727	374.755323
database	2498	ZINC00660395	374.755323
database	1265	ZINC08440218	374.755323
database	2138	ZINC08438769	374.755323
database	2543	ZINC01815196	374.755323
database	2295	ZINC08438658	374.755323
database	2230	ZINC00626525	374.755323

database	802	ZINC00702987	374.755323
database	1118	ZINC00702674	374.755323
database	2501	ZINC08437898	374.755323
database	2495	ZINC08437936	374.755323
database	1615	ZINC00717197	374.755323
database	2185	ZINC08438763	374.755323
database	738	ZINC00703051	374.755323
database	497	ZINC00703120	374.755323
database	2315	ZINC08438568	374.755323
database	817	ZINC16956754	374.755323
database	4156	ZINC08432114	374.755323
database	972	ZINC08440874	374.755323
database	760	ZINC01019935	374.755323
database	2159	ZINC01413516	374.755323
database	3320	ZINC06177078	374.755323
database	1065	ZINC00702696	374.755323
database	4386	ZINC13161176	374.755323
database	771	ZINC00703045	374.755323
database	2303	ZINC08438644	374.755323
database	6349	ZINC02951124	374.755323
database	2243	ZINC06445949	374.755323
database	1075	ZINC00702698	374.755323
database	1298	ZINC00702434	374.755323
database	2119	ZINC01414764	374.755323
database	2260	ZINC08438691	374.755323
database	2108	ZINC01414731	374.755323
database	2265	ZINC01019496	374.755323

database	555	ZINC00703107	374.755323
database	3315	ZINC06136516	374.755323
database	1013	ZINC00702730	374.755323
database	1312	ZINC00702426	374.755323
database	1363	ZINC00702370	374.755323
database	2274	ZINC08438683	374.755323
database	2898	ZINC08903739	374.755323
database	694	ZINC08441470	374.755323
database	3290	ZINC18275977	374.755323
database	822	ZINC08897840	374.755323
database	697	ZINC08441467	374.755323
database	2166	ZINC01413500	374.755323
database	602	ZINC08441614	374.755323
database	1055	ZINC00702700	374.755323
database	1462	ZINC00717893	374.755323
database	2821	ZINC08437173	374.755323
database	2071	ZINC01414783	374.755323
database	1366	ZINC08440096	374.755323
database	2209	ZINC00626750	374.755323
database	740	ZINC00703051	374.755323
database	1306	ZINC08440200	374.755323
database	3683	ZINC08433276	374.755323
database	1019	ZINC00702732	374.755323
database	2306	ZINC01019485	374.755323
database	2130	ZINC01413486	374.755323
database	2211	ZINC00626750	374.755323
database	2151	ZINC01413496	374.755323

database	3748	ZINC06144031	374.755323
database	3301	ZINC08818093	374.755323
database	5157	ZINC08817407	374.755323
database	1740	ZINC08439281	374.755323
database	5789	ZINC08414041	374.755323
database	1295	ZINC00702433	374.755323
database	2066	ZINC01413469	374.755323
database	2007	ZINC08438782	374.755323
database	2337	ZINC08438562	374.755323
database	593	ZINC08441621	374.755323
database	2133	ZINC01413487	374.755323
database	2204	ZINC08438758	374.755323
database	2504	ZINC00660382	374.755323
database	2017	ZINC09374642	374.755323
database	2500	ZINC00660392	374.755323
database	499	ZINC00703121	374.755323
database	1447	ZINC09109829	374.755323
database	1292	ZINC08440205	374.755323
database	1067	ZINC00702697	374.755323
database	5273	ZINC00836245	374.755323
database	1061	ZINC00702702	374.755323
database	5788	ZINC00688634	374.755323
database	1102	ZINC05918675	374.755323
database	2318	ZINC00625874	374.755323
database	1116	ZINC00702674	374.755323
database	2277	ZINC08438681	374.755323
database	1308	ZINC08440200	374.755323

database	1759	ZINC00715098	374.755323
database	581	ZINC08441625	374.755323
database	2299	ZINC08438656	374.755323
database	1016	ZINC00702731	374.755323
database	1283	ZINC08440213	374.755323
database	551	ZINC00703106	374.755323
database	2028	ZINC08438776	374.755323
database	2499	ZINC08437901	374.755323
database	5219	ZINC09271507	374.755323
database	768	ZINC00703044	374.755323
database	575	ZINC01019940	374.755323
database	2289	ZINC08438674	374.755323
database	2374	ZINC08438529	374.755323
database	745	ZINC00703052	374.755323
database	597	ZINC08441616	374.755323
database	1611	ZINC00717199	374.755323
database	2055	ZINC01414773	374.755323
database	2370	ZINC00625480	374.755323
database	3335	ZINC08435575	374.755323
database	3333	ZINC08435576	374.755323
database	1088	ZINC00702690	374.755323
database	2258	ZINC08438693	374.755323
database	2505	ZINC00660380	374.755323
database	2357	ZINC00625733	374.755323
database	2331	ZINC08438565	374.755323
database	3304	ZINC08435580	374.755323
database	2280	ZINC08438679	374.755323

database	1303	ZINC00702428	374.755323
database	572	ZINC08441629	374.755323
database	2024	ZINC08438777	374.755323
database	1756	ZINC00715097	374.755323
database	5155	ZINC08817408	374.755323
database	1301	ZINC00702428	374.755323
database	479	ZINC00703148	374.755323
database	2101	ZINC01413448	374.755323
database	2069	ZINC01414783	374.755323
database	969	ZINC08440877	374.755323
database	900	ZINC08996455	374.755323
database	686	ZINC08441472	374.755323
database	2312	ZINC00625900	374.755323
database	1010	ZINC00702729	374.755323
database	2177	ZINC01413495	374.755323
database	2181	ZINC08438764	374.755323
database	1111	ZINC00702673	374.755323
database	1025	ZINC08440772	374.755323
database	2162	ZINC01413517	374.755323
database	1439	ZINC00721236	374.755323
database	1052	ZINC00702699	374.755323
database	2502	ZINC08437894	374.755323
database	1109	ZINC00702673	374.755323
database	2309	ZINC01019486	374.755323
database	2823	ZINC08437172	374.755323
database	2545	ZINC01764016	374.755323
database	1045	ZINC00702714	374.755323

database	750	ZINC08441403	374.755323
database	2032	ZINC01414769	374.755323
database	2205	ZINC08438758	374.755323
database	1427	ZINC00702308	374.755323
database	978	ZINC00702759	374.755323
database	540	ZINC00971821	187.3776615
database	3667	ZINC00730198	187.3776615
database	5712	ZINC08415472	187.3776615
database	881	ZINC08441103	187.3776615
database	2573	ZINC05945451	187.3776615
database	2450	ZINC00661065	187.3776615
database	4727	ZINC02065557	187.3776615
database	4692	ZINC08430249	187.3776615
database	1634	ZINC04113088	187.3776615
database	1198	ZINC08440465	187.3776615
database	6694	ZINC00687560	187.3776615
database	4601	ZINC08430528	187.3776615
database	4589	ZINC08430572	187.3776615
database	5260	ZINC00836117	187.3776615
database	6451	ZINC02317097	187.3776615
database	5859	ZINC08413894	187.3776615
database	5077	ZINC08426798	187.3776615
database	2433	ZINC00669284	187.3776615
database	1766	ZINC02170045	187.3776615
database	4461	ZINC01026533	187.3776615
database	6537	ZINC00705501	187.3776615
database	1739	ZINC08439281	187.3776615

database	4905	ZINC08427628	187.3776615
database	5855	ZINC08413894	187.3776615
database	2808	ZINC08437199	187.3776615
database	1376	ZINC08440085	187.3776615
database	612	ZINC08441601	187.3776615
database	6458	ZINC19313041	187.3776615
database	5329	ZINC00708339	187.3776615
database	6691	ZINC00687563	187.3776615
database	1730	ZINC02497152	187.3776615
database	475	ZINC00630347	187.3776615
database	5479	ZINC08424944	187.3776615
database	1097	ZINC08440687	187.3776615
database	5826	ZINC08413954	187.3776615
database	5944	ZINC08413415	187.3776615
database	2444	ZINC08438196	187.3776615
database	4629	ZINC08430361	187.3776615
database	1630	ZINC04113089	187.3776615
database	1720	ZINC01013180	187.3776615
database	2432	ZINC00669283	187.3776615
database	5878	ZINC08413878	187.3776615
database	5685	ZINC08415491	187.3776615
database	6030	ZINC08413100	187.3776615
database	6434	ZINC19757571	187.3776615
database	5206	ZINC08426278	187.3776615
database	6038	ZINC08413079	187.3776615
database	5900	ZINC01010011	187.3776615
database	5845	ZINC08413924	187.3776615

database	5890	ZINC08413865	187.3776615
database	635	ZINC00181717	187.3776615
database	5715	ZINC08415466	187.3776615
database	1521	ZINC00643564	187.3776615
database	5302	ZINC06446067	187.3776615
database	5924	ZINC08413491	187.3776615
database	5246	ZINC00836164	187.3776615
database	1532	ZINC08439707	187.3776615
database	5064	ZINC08426812	187.3776615
database	1480	ZINC08439790	187.3776615
database	5970	ZINC08413383	187.3776615
database	5692	ZINC08415486	187.3776615
database	5569	ZINC00799070	187.3776615
database	532	ZINC08441642	187.3776615
database	5060	ZINC08426813	187.3776615
database	860	ZINC00702948	187.3776615
database	3337	ZINC08435574	187.3776615
database	1502	ZINC00630228	187.3776615
database	4716	ZINC08430219	187.3776615
database	1237	ZINC00702477	187.3776615
database	6224	ZINC08411232	187.3776615
database	3554	ZINC00123806	187.3776615
database	3236	ZINC00976119	187.3776615
database	4232	ZINC08431778	187.3776615
database	7169	ZINC00675545	187.3776615
database	1590	ZINC02170779	187.3776615
database	539	ZINC00971822	187.3776615

database	6715	ZINC00687467	187.3776615
database	3762	ZINC01017579	187.3776615
database	6729	ZINC00687438	187.3776615
database	4635	ZINC08430354	187.3776615
database	6546	ZINC01022589	187.3776615
database	5952	ZINC08413407	187.3776615
database	4639	ZINC08430353	187.3776615
database	5906	ZINC08413556	187.3776615
database	4719	ZINC08430219	187.3776615
database	1340	ZINC08440140	187.3776615
database	3234	ZINC00976121	187.3776615
database	4025	ZINC00669643	187.3776615
database	5544	ZINC08424503	187.3776615
database	4140	ZINC17044116	187.3776615
database	5055	ZINC08426820	187.3776615
database	1825	ZINC04198939	187.3776615
database	5301	ZINC08425969	187.3776615
database	4822	ZINC08430011	187.3776615
database	3340	ZINC08435573	187.3776615
database	6042	ZINC08413079	187.3776615
database	3538	ZINC00194938	187.3776615
database	5718	ZINC08415461	187.3776615
database	5902	ZINC01010013	187.3776615
database	3206	ZINC00976168	187.3776615
database	6033	ZINC08413085	187.3776615
database	3635	ZINC00974113	187.3776615
database	858	ZINC00702947	187.3776615

database	3994	ZINC08818437	187.3776615
database	533	ZINC08441641	187.3776615
database	211	ZINC08442062	187.3776615
database	3891	ZINC08432907	187.3776615
database	1648	ZINC02170620	187.3776615
database	6545	ZINC01022588	187.3776615
database	4704	ZINC02065636	187.3776615
database	6072	ZINC01009886	187.3776615
database	5248	ZINC00780341	187.3776615
database	2766	ZINC06444576	187.3776615
database	6487	ZINC00705716	187.3776615
database	680	ZINC04180917	187.3776615
database	916	ZINC00850103	187.3776615
database	5991	ZINC08413182	187.3776615
database	6461	ZINC19312985	187.3776615
database	1545	ZINC02170892	187.3776615
database	3883	ZINC02566679	187.3776615
database	5849	ZINC08413924	187.3776615
database	2905	ZINC08436997	187.3776615
database	4141	ZINC17044111	187.3776615
database	4023	ZINC00628150	187.3776615
database	1797	ZINC02169664	187.3776615
database	853	ZINC05313283	187.3776615
database	6730	ZINC00687437	187.3776615
database	4984	ZINC08427321	187.3776615
database	4516	ZINC00996973	187.3776615
database	4638	ZINC08430354	187.3776615

database	2577	ZINC08437722	187.3776615
database	6356	ZINC00984823	187.3776615
database	3231	ZINC06442929	187.3776615
database	69	ZINC08442186	187.3776615
database	6513	ZINC03103564	187.3776615
database	869	ZINC08441127	187.3776615
database	7013	ZINC00676754	187.3776615
database	5722	ZINC08415447	187.3776615
database	4887	ZINC08427630	187.3776615
database	3926	ZINC08432852	187.3776615
database	4688	ZINC04003842	187.3776615
database	852	ZINC05313283	187.3776615
database	4164	ZINC00668291	187.3776615
database	1345	ZINC08440140	187.3776615
database	5843	ZINC08413942	187.3776615
database	5411	ZINC00708086	187.3776615
database	5714	ZINC08415472	187.3776615
database	3898	ZINC00632115	187.3776615
database	6460	ZINC19312985	187.3776615
database	3998	ZINC00986157	187.3776615
database	6355	ZINC00984823	187.3776615
database	2742	ZINC08437418	187.3776615
database	873	ZINC00702930	187.3776615
database	3942	ZINC00628526	187.3776615
database	5296	ZINC08425972	187.3776615
database	911	ZINC08441036	187.3776615
database	5927	ZINC08413491	187.3776615

database	3914	ZINC00710388	187.3776615
database	3465	ZINC00655680	187.3776615
database	6459	ZINC19313041	187.3776615
database	1240	ZINC00702477	187.3776615
database	5887	ZINC08413865	187.3776615
database	5727	ZINC08415310	187.3776615
database	5840	ZINC08413942	187.3776615
database	5073	ZINC08426799	187.3776615
database	1621	ZINC08439535	187.3776615
database	3293	ZINC00676053	187.3776615
database	1837	ZINC08439070	187.3776615
database	4603	ZINC08430525	187.3776615
database	4630	ZINC08430357	187.3776615
database	4894	ZINC08427629	187.3776615
database	1591	ZINC02170776	187.3776615
database	3996	ZINC08927626	187.3776615
database	5837	ZINC08413952	187.3776615
database	5669	ZINC08415697	187.3776615
database	6727	ZINC00687440	187.3776615
database	212	ZINC08442062	187.3776615
database	1474	ZINC08439794	187.3776615
database	4517	ZINC00996974	187.3776615
database	5670	ZINC08415693	187.3776615
database	5942	ZINC08413440	187.3776615
database	5475	ZINC08424949	187.3776615
database	56	ZINC08442214	187.3776615
database	1241	ZINC00702469	187.3776615

database	7033	ZINC00999324	187.3776615
database	4886	ZINC08427631	187.3776615
database	5793	ZINC01010093	187.3776615
database	2599	ZINC08437700	187.3776615
database	4600	ZINC08430530	187.3776615
database	5057	ZINC01027227	187.3776615
database	4599	ZINC08430533	187.3776615
database	5738	ZINC08414959	187.3776615
database	6485	ZINC00705719	187.3776615
database	4705	ZINC02065637	187.3776615
database	5068	ZINC08426811	187.3776615
database	6456	ZINC19320421	187.3776615
database	5253	ZINC00780342	187.3776615
database	4138	ZINC08995930	187.3776615
database	2574	ZINC05945442	187.3776615
database	5409	ZINC00708084	187.3776615
database	1769	ZINC00621935	187.3776615
database	4022	ZINC02085622	187.3776615
database	5825	ZINC08413966	187.3776615
database	6576	ZINC01022410	187.3776615
database	5986	ZINC08413236	187.3776615
database	6024	ZINC08413106	187.3776615
database	1570	ZINC00629164	187.3776615
database	2804	ZINC08437204	187.3776615
database	537	ZINC00971824	187.3776615
database	5726	ZINC08415314	187.3776615
database	3622	ZINC00788120	187.3776615

database	1721	ZINC08439304	187.3776615
database	5564	ZINC00707161	187.3776615
database	6542	ZINC00705440	187.3776615
database	1746	ZINC02694664	187.3776615
database	1157	ZINC08440584	187.3776615
database	1477	ZINC08439792	187.3776615
database	1626	ZINC08439530	187.3776615
database	1130	ZINC08440596	187.3776615
database	5408	ZINC00708085	187.3776615
database	1605	ZINC00717217	187.3776615
database	3466	ZINC00655678	187.3776615
database	6037	ZINC08413085	187.3776615
database	1694	ZINC08439343	187.3776615
database	4328	ZINC00870831	187.3776615
database	5263	ZINC00836121	187.3776615
database	5721	ZINC08415450	187.3776615
database	7131	ZINC00999199	187.3776615
database	4879	ZINC08427631	187.3776615
database	5719	ZINC08415458	187.3776615
database	3925	ZINC08432853	187.3776615
database	5948	ZINC08413411	187.3776615
database	5821	ZINC08413973	187.3776615
database	3623	ZINC00788122	187.3776615
database	5230	ZINC02475452	187.3776615
database	5905	ZINC08413584	187.3776615
database	5823	ZINC08413973	187.3776615
database	6692	ZINC00687562	187.3776615

database	5995	ZINC08413134	187.3776615
database	5786	ZINC08414147	187.3776615
database	3673	ZINC00728061	187.3776615
database	4489	ZINC00984135	187.3776615
database	6811	ZINC00687267	187.3776615
database	5070	ZINC08426810	187.3776615
database	5243	ZINC00836161	187.3776615
database	4990	ZINC03087694	187.3776615
database	5872	ZINC08413885	187.3776615
database	4587	ZINC08430576	187.3776615
database	3932	ZINC08432844	187.3776615
database	6071	ZINC01009885	187.3776615
database	4024	ZINC00669641	187.3776615
database	3908	ZINC00628537	187.3776615
database	1981	ZINC08438827	187.3776615
database	1729	ZINC02497152	187.3776615
database	1197	ZINC08440465	187.3776615
database	4897	ZINC08427629	187.3776615
database	5901	ZINC01010012	187.3776615
database	3150	ZINC00907486	187.3776615
database	5335	ZINC00708331	187.3776615
database	5899	ZINC01010018	187.3776615
database	4890	ZINC08427630	187.3776615
database	3852	ZINC00628758	187.3776615
database	5244	ZINC00836161	187.3776615
database	5797	ZINC08413985	187.3776615
database	5791	ZINC01010091	187.3776615

database	2768	ZINC06444558	187.3776615
database	3794	ZINC00632285	187.3776615
database	5166	ZINC08426425	187.3776615
database	6055	ZINC08413061	187.3776615
database	5965	ZINC08413387	187.3776615
database	6728	ZINC00687439	187.3776615
database	5862	ZINC08413890	187.3776615
database	5432	ZINC01024748	187.3776615
database	5903	ZINC01010014	187.3776615
database	1236	ZINC00702484	187.3776615
database	1544	ZINC02170889	187.3776615
database	5338	ZINC00708332	187.3776615
database	875	ZINC00702927	187.3776615
database	5699	ZINC08415481	187.3776615
database	5255	ZINC00836126	187.3776615
database	5720	ZINC08415458	187.3776615
database	5686	ZINC08415486	187.3776615
database	2932	ZINC01019220	187.3776615
database	4591	ZINC08430571	187.3776615
database	6223	ZINC08411232	187.3776615
database	5671	ZINC08415497	187.3776615
database	5330	ZINC00708340	187.3776615
database	4909	ZINC08427628	187.3776615
database	5331	ZINC00827963	187.3776615
database	3521	ZINC00195057	187.3776615
database	5993	ZINC08413182	187.3776615
database	5860	ZINC08413890	187.3776615

database	6058	ZINC08413048	187.3776615
database	4036	ZINC00628446	187.3776615
database	3213	ZINC00867609	187.3776615
database	5167	ZINC08426422	187.3776615
database	1540	ZINC08439703	187.3776615
database	3927	ZINC08432851	187.3776615
database	6455	ZINC19320421	187.3776615
database	4721	ZINC08430218	187.3776615
database	1719	ZINC01013182	187.3776615
database	3884	ZINC00332968	187.3776615
database	1833	ZINC02497229	187.3776615
database	3591	ZINC00194823	187.3776615
database	1433	ZINC08439923	187.3776615
database	865	ZINC00702935	187.3776615
database	3890	ZINC08432908	187.3776615
database	5716	ZINC08415466	187.3776615
database	5693	ZINC08415481	187.3776615
database	6491	ZINC01022739	187.3776615
database	4021	ZINC02085621	187.3776615
database	4991	ZINC00711258	187.3776615
database	1800	ZINC01012880	187.3776615
database	2585	ZINC08437719	187.3776615
database	5971	ZINC08413383	187.3776615
database	3067	ZINC06444403	187.3776615
database	1238	ZINC00702477	187.3776615
database	3902	ZINC00710426	187.3776615
database	1657	ZINC06321516	187.3776615

database	5256	ZINC08426069	187.3776615
database	5674	ZINC08415497	187.3776615
database	5981	ZINC08413327	187.3776615
database	5893	ZINC01010032	187.3776615
database	4149	ZINC00707041	187.3776615
database	5882	ZINC08413868	187.3776615
database	5056	ZINC01027229	187.3776615
database	5983	ZINC08413236	187.3776615
database	5562	ZINC00707160	187.3776615
database	1665	ZINC00643192	187.3776615
database	4985	ZINC08427321	187.3776615
database	5909	ZINC08413551	187.3776615
database	5332	ZINC00827963	187.3776615
database	4989	ZINC00711263	187.3776615
database	3791	ZINC01017546	187.3776615
database	5892	ZINC01010031	187.3776615
database	6348	ZINC02951124	187.3776615
database	5407	ZINC00708083	187.3776615
database	5683	ZINC08415491	187.3776615
database	5300	ZINC08425969	187.3776615
database	5194	ZINC08426285	187.3776615
database	3778	ZINC00723976	187.3776615
database	3915	ZINC00710387	187.3776615
database	213	ZINC08442062	187.3776615
database	1768	ZINC00621935	187.3776615
database	3149	ZINC00907493	187.3776615
database	6514	ZINC03103563	187.3776615

database	2806	ZINC08437202	187.3776615
database	4588	ZINC08430574	187.3776615
database	3668	ZINC00730196	187.3776615
database	3409	ZINC08435455	187.3776615
database	5488	ZINC08424914	187.3776615
database	4139	ZINC17044107	187.3776615
database	3338	ZINC08435574	187.3776615
database	2569	ZINC05945448	187.3776615
database	4614	ZINC00996358	187.3776615
database	1374	ZINC08440085	187.3776615
database	4595	ZINC00996453	187.3776615
database	5202	ZINC08426280	187.3776615
database	4717	ZINC08430219	187.3776615
database	5471	ZINC08424954	187.3776615
database	6004	ZINC08413118	187.3776615
database	996	ZINC00647877	187.3776615
database	5265	ZINC00677994	187.3776615
database	3820	ZINC00719622	187.3776615
database	618	ZINC08441595	187.3776615
database	1354	ZINC00828732	187.3776615
database	2580	ZINC08437721	187.3776615
database	5250	ZINC00780341	187.3776615
database	1431	ZINC02061254	187.3776615
database	663	ZINC00241369	187.3776615
database	3227	ZINC06442944	187.3776615
database	3923	ZINC08432865	187.3776615
database	679	ZINC04180918	187.3776615

database	5337	ZINC00708332	187.3776615
database	3995	ZINC08818436	187.3776615
database	538	ZINC00971823	187.3776615
database	5262	ZINC00836121	187.3776615
database	4262	ZINC00703690	187.3776615
database	5433	ZINC03669786	187.3776615
database	210	ZINC08442062	187.3776615
database	4689	ZINC04003838	187.3776615
database	1560	ZINC08439649	187.3776615
database	4901	ZINC08427629	187.3776615
database	1082	ZINC08440704	187.3776615
database	1348	ZINC00842712	187.3776615
database	4878	ZINC08427631	187.3776615
database	867	ZINC00702936	187.3776615
database	6492	ZINC01022740	187.3776615
database	5706	ZINC08415476	187.3776615
database	5735	ZINC08414961	187.3776615
database	3504	ZINC00678329	187.3776615
database	5833	ZINC08413952	187.3776615
database	6486	ZINC00705720	187.3776615
database	1071	ZINC08440711	187.3776615
database	1369	ZINC08440095	187.3776615
database	6575	ZINC01022409	187.3776615
database	4687	ZINC04003841	187.3776615
database	4385	ZINC13161176	187.3776615
database	1622	ZINC08439535	187.3776615
database	871	ZINC08441114	187.3776615

database	5336	ZINC00708331	187.3776615
database	1203	ZINC00702554	187.3776615
database	1651	ZINC08439418	187.3776615
database	6726	ZINC00687444	187.3776615
database	5467	ZINC08424956	187.3776615
database	5897	ZINC01010016	187.3776615
database	1484	ZINC08439788	187.3776615
database	2594	ZINC08437705	187.3776615
database	3229	ZINC06442961	187.3776615
database	1676	ZINC08439378	187.3776615
database	1243	ZINC00702465	187.3776615
database	4888	ZINC08427630	187.3776615
database	6018	ZINC08413106	187.3776615
database	2760	ZINC00694033	187.3776615
database	1669	ZINC08439383	187.3776615
database	4722	ZINC08430218	187.3776615
database	4426	ZINC00984560	187.3776615
database	5977	ZINC08413327	187.3776615
database	1204	ZINC00702554	187.3776615
database	1624	ZINC08439530	187.3776615
database	4927	ZINC00711873	187.3776615
database	4032	ZINC02679198	187.3776615
database	1432	ZINC08439925	187.3776615
database	4992	ZINC00711260	187.3776615
database	5988	ZINC04718185	187.3776615
database	5812	ZINC08413980	187.3776615
database	1076	ZINC08440709	187.3776615

database	4299	ZINC08994408	187.3776615
database	5054	ZINC08426821	187.3776615
database	5895	ZINC01010034	187.3776615
database	534	ZINC00971825	187.3776615
database	3341	ZINC08435573	187.3776615
database	3889	ZINC08432909	187.3776615
database	6428	ZINC03664825	187.3776615
database	467	ZINC00630388	187.3776615
database	6693	ZINC00687561	187.3776615
database	6026	ZINC08413100	187.3776615
database	3406	ZINC08435456	187.3776615
database	3135	ZINC00812969	187.3776615
database	5898	ZINC01010017	187.3776615
database	1498	ZINC08439765	187.3776615
database	4696	ZINC08430248	187.3776615
database	1093	ZINC08440699	187.3776615
database	1550	ZINC08439659	187.3776615
database	1659	ZINC06321511	187.3776615
database	4163	ZINC00628266	187.3776615
database	1787	ZINC02497202	187.3776615
database	6690	ZINC00687564	187.3776615
database	1832	ZINC02497229	187.3776615
database	1623	ZINC08439535	187.3776615
database	5831	ZINC08413954	187.3776615
database	5790	ZINC01010090	187.3776615
database	4882	ZINC08427631	187.3776615
database	1555	ZINC08439654	187.3776615

database	2434	ZINC08438232	187.3776615
database	541	ZINC02998601	187.3776615
database	1642	ZINC04113086	187.3776615
database	4642	ZINC08430353	187.3776615
database	6543	ZINC08404584	187.3776615
database	3928	ZINC08432850	187.3776615
database	2453	ZINC00661067	187.3776615
database	1538	ZINC08439704	187.3776615
database	3235	ZINC00976120	187.3776615
database	5668	ZINC08416432	187.3776615
database	1535	ZINC08439705	187.3776615
database	5316	ZINC08425758	187.3776615
database	6540	ZINC01022604	187.3776615
database	3295	ZINC00676053	187.3776615
database	1242	ZINC00702469	187.3776615
database	5678	ZINC08415491	187.3776615
database	6000	ZINC08413118	187.3776615
database	6043	ZINC08413066	187.3776615
database	1625	ZINC08439530	187.3776615
database	4119	ZINC00707055	187.3776615
database	6544	ZINC08404581	187.3776615
database	4691	ZINC04003840	187.3776615
database	3294	ZINC00676053	187.3776615
database	1765	ZINC02170042	187.3776615
database	6731	ZINC00687436	187.3776615
database	1137	ZINC08440596	187.3776615
database	3214	ZINC00867608	187.3776615

database	6539	ZINC01022603	187.3776615
database	66	ZINC08442186	187.3776615
database	2452	ZINC00624531	187.3776615
database	1235	ZINC00702484	187.3776615
database	1638	ZINC04113087	187.3776615
database	917	ZINC08441032	187.3776615
database	5904	ZINC08413588	187.3776615
database	857	ZINC02063810	187.3776615
database	915	ZINC00850102	187.3776615
database	879	ZINC00702925	187.3776615
database	6057	ZINC08413048	187.3776615
database	5725	ZINC08415317	187.3776615
database	5807	ZINC08413980	187.3776615
database	5259	ZINC00836117	187.3776615
database	637	ZINC00181552	187.3776615
database	4633	ZINC08430357	187.3776615
database	5999	ZINC08413134	187.3776615
database	3505	ZINC00678328	187.3776615
database	4928	ZINC00711875	187.3776615
database	5717	ZINC08415461	187.3776615
database	5896	ZINC01010015	187.3776615
database	5251	ZINC00780342	187.3776615
database	5707	ZINC08415472	187.3776615
database	1119	ZINC08440677	187.3776615
database	5254	ZINC00836126	187.3776615
database	662	ZINC08441519	187.3776615
database	1497	ZINC00630257	187.3776615

database	4893	ZINC08427630	187.3776615
database	1696	ZINC08439341	187.3776615
database	2595	ZINC08437704	187.3776615
database	1239	ZINC00702477	187.3776615
database	3892	ZINC08432906	187.3776615
database	1600	ZINC00717216	187.3776615
database	2582	ZINC08437720	187.3776615
database	3929	ZINC08432849	187.3776615
database	1349	ZINC00842713	187.3776615
database	79	ZINC08442163	187.3776615
database	5297	ZINC08425972	187.3776615
database	5881	ZINC08413878	187.3776615
database	5334	ZINC00708331	187.3776615
database	2743	ZINC08437418	187.3776615
database	1503	ZINC00630229	187.3776615
database	476	ZINC00630276	187.3776615
database	7132	ZINC00999199	187.3776615
database	4902	ZINC08427628	187.3776615
database	5865	ZINC08413888	187.3776615
database	5257	ZINC08426069	187.3776615
database	6082	ZINC08411698	187.3776615
database	1079	ZINC08440708	187.3776615
database	2744	ZINC08437418	187.3776615
database	4109	ZINC00870337	187.3776615
database	5700	ZINC08415476	187.3776615
database	2597	ZINC08437702	187.3776615
database	1592	ZINC00717214	187.3776615

database	1546	ZINC08439662	187.3776615
database	1795	ZINC02169720	187.3776615
database	2881	ZINC08437085	187.3776615
database	1068	ZINC08440712	187.3776615
database	2810	ZINC08437198	187.3776615
database	5198	ZINC08426283	187.3776615
database	877	ZINC00702928	187.3776615
database	2571	ZINC05945440	187.3776615
database	1767	ZINC02170045	187.3776615
database	1596	ZINC00717215	187.3776615
database	2598	ZINC08437702	187.3776615
database	1653	ZINC08439415	187.3776615
database	3225	ZINC06442984	187.3776615
database	1822	ZINC02497224	187.3776615
database	4626	ZINC08430361	187.3776615
database	5485	ZINC08424915	187.3776615
database	5247	ZINC00836164	187.3776615
database	2435	ZINC08438231	187.3776615
database	2592	ZINC00623095	187.3776615
database	1542	ZINC00643446	187.3776615
database	6535	ZINC00705503	187.3776615
database	6695	ZINC00687559	187.3776615
database	6541	ZINC00705439	187.3776615
database	1534	ZINC08439706	187.3776615
database	4233	ZINC08431778	187.3776615
database	3634	ZINC08434753	187.3776615
database	5886	ZINC08413868	187.3776615

database	4300	ZINC08994407	187.3776615
database	4724	ZINC08430218	187.3776615
database	1664	ZINC00643194	187.3776615
database	6452	ZINC02317098	187.3776615
database	4136	ZINC01250552	187.3776615
database	5264	ZINC00677994	187.3776615
database	6019	ZINC08413106	187.3776615
database	6290	ZINC02492153	187.3776615
database	1095	ZINC08440687	187.3776615
database	4007	ZINC01162829	187.3776615
database	2600	ZINC08437700	187.3776615
database	1732	ZINC08439285	187.3776615
database	320	ZINC08441964	187.3776615
database	1764	ZINC02170042	187.3776615
database	1430	ZINC02484263	187.3776615
database	5894	ZINC01010033	187.3776615
database	3924	ZINC08432855	187.3776615
database	6536	ZINC00705504	187.3776615
database	1786	ZINC02497202	187.3776615
database	6427	ZINC08406651	187.3776615
database	1122	ZINC08440677	187.3776615
database	5792	ZINC01010092	187.3776615
database	58	ZINC08442210	187.3776615
database	4613	ZINC00996358	187.3776615
database	6538	ZINC00705502	187.3776615
database	5989	ZINC04718185	187.3776615
database	1649	ZINC02170616	187.3776615

database	2734	ZINC08437427	0
database	596	ZINC08441616	0
database	3698	ZINC09271701	0
database	3742	ZINC02895344	0
database	2034	ZINC01414770	0
database	6789	ZINC00687341	0
database	7029	ZINC00676679	0
database	3217	ZINC00857428	0
database	1476	ZINC08439794	0
database	595	ZINC08441616	0
database	5813	ZINC08413980	0
database	4186	ZINC13775163	0
database	2220	ZINC08438703	0
database	7197	ZINC08400284	0
database	6265	ZINC04168776	0
database	4818	ZINC00995994	0
database	5130	ZINC06245044	0
database	2953	ZINC06144544	0
database	991	ZINC08440849	0
database	2625	ZINC01011079	0
database	710	ZINC15884982	0
database	5364	ZINC00708251	0
database	5750	ZINC08414746	0
database	3739	ZINC01017590	0
database	5572	ZINC02057086	0
database	2228	ZINC00626525	0
database	6815	ZINC08400811	0

database	2916	ZINC01803477	0
database	5012	ZINC08427108	0
database	2424	ZINC08829718	0
database	6906	ZINC08400729	0
database	5103	ZINC08426767	0
database	4543	ZINC13601787	0
database	6735	ZINC00687429	0
database	1195	ZINC00702562	0
database	2841	ZINC08437147	0
database	7073	ZINC08400484	0
database	2863	ZINC00646442	0
database	5047	ZINC08426844	0
database	2259	ZINC08438691	0
database	2884	ZINC08437080	0
database	823	ZINC08897840	0
database	5367	ZINC08425602	0
database	1547	ZINC08439662	0
database	1883	ZINC00627559	0
database	2555	ZINC02162277	0
database	1257	ZINC02062114	0
database	7090	ZINC02252328	0
database	5777	ZINC08414287	0
database	4352	ZINC00991572	0
database	4094	ZINC01794772	0
database	6416	ZINC08407228	0
database	5913	ZINC08413520	0
database	6872	ZINC00686684	0

database	1965	ZINC01011872	0
database	183	ZINC06303157	0
database	1785	ZINC08439180	0
database	7098	ZINC01008132	0
database	1284	ZINC00848684	0
database	362	ZINC08441913	0
database	407	ZINC00881949	0
database	5592	ZINC00693000	0
database	5966	ZINC08413387	0
database	5240	ZINC00643463	0
database	5195	ZINC08426285	0
database	951	ZINC00648073	0
database	1635	ZINC04113088	0
database	1780	ZINC02169882	0
database	3953	ZINC06659995	0
database	2897	ZINC08903739	0
database	4117	ZINC02680115	0
database	4077	ZINC08432385	0
database	6235	ZINC13136834	0
database	3934	ZINC00249652	0
database	2456	ZINC00624443	0
database	46	ZINC08442272	0
database	5185	ZINC00839005	0
database	4766	ZINC08430149	0
database	5590	ZINC08417802	0
database	944	ZINC00970849	0
database	960	ZINC06445743	0

database	2242	ZINC06445949	0
database	7100	ZINC08400465	0
database	3415	ZINC00827958	0
database	6862	ZINC00686708	0
database	6755	ZINC00687404	0
database	6261	ZINC00987206	0
database	6797	ZINC00687327	0
database	6846	ZINC00686819	0
database	5746	ZINC08414751	0
database	313	ZINC06446604	0
database	4006	ZINC05748687	0
database	1293	ZINC00702433	0
database	5636	ZINC01010552	0
database	4758	ZINC08430155	0
database	42	ZINC08442278	0
database	1246	ZINC02666666	0
database	4100	ZINC08432296	0
database	2615	ZINC08437693	0
database	6114	ZINC00688053	0
database	5641	ZINC02153808	0
database	2061	ZINC01413468	0
database	4168	ZINC02084907	0
database	2161	ZINC01413517	0
database	6505	ZINC19369484	0
database	2892	ZINC02156644	0
database	1381	ZINC00702349	0
database	5731	ZINC08415069	0

database	1452	ZINC09008533	0
database	4129	ZINC08432203	0
database	4609	ZINC00983556	0
database	5365	ZINC00708252	0
database	5033	ZINC08426974	0
database	2940	ZINC00645584	0
database	5306	ZINC01830774	0
database	2694	ZINC06147718	0
database	3766	ZINC02106712	0
database	1619	ZINC08439539	0
database	5396	ZINC02285986	0
database	6768	ZINC00687392	0
database	3119	ZINC00976278	0
database	3779	ZINC08433139	0
database	628	ZINC00857151	0
database	5099	ZINC08426773	0
database	4841	ZINC08429973	0
database	52	ZINC00666987	0
database	6647	ZINC00687680	0
database	4448	ZINC08431223	0
database	6220	ZINC08411267	0
database	4661	ZINC08430309	0
database	3367	ZINC08995859	0
database	529	ZINC15952847	0
database	4336	ZINC00703588	0
database	401	ZINC01860012	0
database	162	ZINC00664621	0

database	6237	ZINC13132812	0
database	6994	ZINC08400612	0
database	4915	ZINC08427614	0
database	7130	ZINC08400424	0
database	6896	ZINC00686582	0
database	1128	ZINC00702636	0
database	984	ZINC00702761	0
database	2310	ZINC00625900	0
database	6002	ZINC08413118	0
database	5996	ZINC08413134	0
database	4981	ZINC08427360	0
database	5322	ZINC00647606	0
database	4315	ZINC00862954	0
database	6091	ZINC04457875	0
database	3508	ZINC00678273	0
database	3453	ZINC00662865	0
database	3435	ZINC00663228	0
database	354	ZINC08441920	0
database	1697	ZINC08439341	0
database	4455	ZINC09065134	0
database	5625	ZINC00312408	0
database	2608	ZINC01011102	0
database	5864	ZINC08413890	0
database	5755	ZINC08414700	0
database	1891	ZINC00627468	0
database	3922	ZINC00710364	0
database	5460	ZINC00833682	0

database	1709	ZINC08439320	0
database	5526	ZINC00627179	0
database	3710	ZINC08433222	0
database	2729	ZINC08437430	0
database	2820	ZINC08437173	0
database	4889	ZINC08427630	0
database	6148	ZINC02846827	0
database	2539	ZINC00623500	0
database	655	ZINC00859989	0
database	5582	ZINC00863104	0
database	712	ZINC15884984	0
database	7023	ZINC02158339	0
database	5112	ZINC08426765	0
database	294	ZINC08441983	0
database	7180	ZINC05045065	0
database	6488	ZINC08405625	0
database	5389	ZINC06446032	0
database	2390	ZINC08438427	0
database	4602	ZINC08430526	0
database	3636	ZINC00889266	0
database	3397	ZINC06473864	0
database	1356	ZINC00849729	0
database	4735	ZINC08430174	0
database	3289	ZINC20032743	0
database	5428	ZINC00708064	0
database	3816	ZINC08433050	0
database	3429	ZINC00845543	0

database	3296	ZINC08817352	0
database	6807	ZINC02146020	0
database	654	ZINC00860537	0
database	5398	ZINC08425458	0
database	614	ZINC08441601	0
database	2420	ZINC08438279	0
database	4477	ZINC08431050	0
database	2232	ZINC00626535	0
database	4042	ZINC08432500	0
database	4406	ZINC08431390	0
database	5920	ZINC08413517	0
database	6473	ZINC01022827	0
database	5846	ZINC08413924	0
database	3689	ZINC08433259	0
database	4310	ZINC02742361	0
database	2342	ZINC00625755	0
database	1990	ZINC02165104	0
database	410	ZINC00881917	0
database	4908	ZINC08427628	0
database	3809	ZINC00723827	0
database	2308	ZINC01019486	0
database	4097	ZINC08432297	0
database	1946	ZINC00627111	0
database	5187	ZINC06446990	0
database	4718	ZINC08430219	0
database	88	ZINC08442122	0
database	7072	ZINC08400484	0

database	5229	ZINC00969703	0
database	464	ZINC00881578	0
database	5673	ZINC08415497	0
database	126	ZINC00664787	0
database	4494	ZINC00037806	0
database	5100	ZINC08426773	0
database	6297	ZINC00985879	0
database	7221	ZINC08400213	0
database	3619	ZINC08434812	0
database	2475	ZINC08438068	0
database	400	ZINC02064380	0
database	2123	ZINC01011732	0
database	3278	ZINC08435586	0
database	2217	ZINC08438704	0
database	6354	ZINC08410134	0
database	141	ZINC08442088	0
database	184	ZINC06303157	0
database	4447	ZINC00072909	0
database	3203	ZINC00976194	0
database	562	ZINC00703108	0
database	6723	ZINC08400816	0
database	2273	ZINC08438683	0
database	2391	ZINC08438424	0
database	736	ZINC00703051	0
database	7122	ZINC08400437	0
database	4182	ZINC01814152	0
database	2885	ZINC08437079	0

database	6076	ZINC01009832	0
database	1845	ZINC02497214	0
database	1834	ZINC08439073	0
database	358	ZINC08441915	0
database	2914	ZINC08436971	0
database	3609	ZINC00816791	0
database	6089	ZINC08411690	0
database	5921	ZINC08413517	0
database	6812	ZINC02144229	0
database	4804	ZINC02144296	0
database	3989	ZINC08432678	0
database	660	ZINC13123055	0
database	4103	ZINC08432294	0
database	3813	ZINC08433064	0
database	2466	ZINC00660708	0
database	2662	ZINC01113150	0
database	1180	ZINC00702588	0
database	5452	ZINC08425099	0
database	2110	ZINC01414732	0
database	1041	ZINC00702713	0
database	1496	ZINC00645878	0
database	4093	ZINC02745735	0
database	51	ZINC00666987	0
database	997	ZINC00848995	0
database	4241	ZINC06232509	0
database	3626	ZINC01952505	0
database	1781	ZINC02169869	0

database	3999	ZINC00670488	0
database	6856	ZINC00686745	0
database	2964	ZINC00645577	0
database	1507	ZINC00630191	0
database	4260	ZINC00984971	0
database	5489	ZINC08424914	0
database	2893	ZINC08437044	0
database	4019	ZINC02085636	0
database	4224	ZINC00997828	0
database	324	ZINC08441956	0
database	6641	ZINC08400930	0
database	3102	ZINC00883794	0
database	7004	ZINC00676808	0
database	3981	ZINC00710221	0
database	2935	ZINC00645590	0
database	2392	ZINC08438422	0
database	2637	ZINC08437678	0
database	6498	ZINC19369493	0
database	209	ZINC13161527	0
database	6644	ZINC00687791	0
database	5690	ZINC08415486	0
database	2511	ZINC00624095	0
database	5023	ZINC08427048	0
database	2679	ZINC00646878	0
database	4323	ZINC02179073	0
database	6203	ZINC08411308	0
database	4434	ZINC08431302	0

database	3693	ZINC17163311	0
database	3825	ZINC08433025	0
database	3545	ZINC08435018	0
database	3392	ZINC02071516	0
database	3121	ZINC00976275	0
database	1576	ZINC08439614	0
database	6163	ZINC08411417	0
database	1598	ZINC00717215	0
database	4743	ZINC08430167	0
database	3692	ZINC00728010	0
database	4747	ZINC06287773	0
database	463	ZINC00881582	0
database	758	ZINC01019935	0
database	4555	ZINC08430671	0
database	4158	ZINC00751988	0
database	6640	ZINC08400934	0
database	1404	ZINC08439974	0
database	5148	ZINC08426529	0
database	427	ZINC08441879	0
database	57	ZINC08442214	0
database	3073	ZINC08435661	0
database	1708	ZINC08439320	0
database	5224	ZINC00969728	0
database	289	ZINC00660155	0
database	196	ZINC04167831	0
database	2609	ZINC01011098	0
database	2699	ZINC08437491	0

database	2639	ZINC00622873	0
database	550	ZINC00703106	0
database	6683	ZINC00687599	0
database	3029	ZINC00645505	0
database	7125	ZINC02135751	0
database	99	ZINC08442116	0
database	7007	ZINC01008216	0
database	6868	ZINC02835492	0
database	6145	ZINC08411455	0
database	1132	ZINC08440596	0
database	494	ZINC00703129	0
database	451	ZINC00881648	0
database	6750	ZINC00687409	0
database	1018	ZINC00702732	0
database	2705	ZINC00149203	0
database	2664	ZINC00622712	0
database	5567	ZINC13893243	0
database	3961	ZINC00986403	0
database	1957	ZINC00627054	0
database	1508	ZINC00381968	0
database	7051	ZINC08400510	0
database	5476	ZINC08424949	0
database	1867	ZINC01012622	0
database	5956	ZINC08413407	0
database	4728	ZINC02065553	0
database	4294	ZINC00845639	0
database	1469	ZINC08439871	0

database	5570	ZINC08424377	0
database	2776	ZINC00646486	0
database	2997	ZINC00645563	0
database	1826	ZINC08439094	0
database	2091	ZINC01414751	0
database	7014	ZINC02135936	0
database	1299	ZINC00702428	0
database	3846	ZINC01801810	0
database	266	ZINC08442009	0
database	684	ZINC08441472	0
database	3507	ZINC08435121	0
database	6176	ZINC08411403	0
database	2314	ZINC08438568	0
database	1451	ZINC09122892	0
database	2653	ZINC08437659	0
database	3747	ZINC06144031	0
database	1255	ZINC00830700	0
database	5606	ZINC00713385	0
database	201	ZINC08442069	0
database	3782	ZINC08433138	0
database	5828	ZINC08413954	0
database	5288	ZINC08426006	0
database	5177	ZINC00678144	0
database	3930	ZINC08432847	0
database	3375	ZINC00868353	0
database	1670	ZINC08439383	0
database	6895	ZINC00686583	0

database	5575	ZINC00827398	0
database	3512	ZINC08435081	0
database	5495	ZINC00258076	0
database	5269	ZINC08426016	0
database	7108	ZINC01008130	0
database	6599	ZINC19781721	0
database	6385	ZINC08407977	0
database	2296	ZINC08438658	0
database	6820	ZINC08400808	0
database	4582	ZINC00667456	0
database	4764	ZINC08430150	0
database	772	ZINC00856090	0
database	3424	ZINC08435392	0
database	5400	ZINC01024806	0
database	4954	ZINC00291192	0
database	3090	ZINC00884091	0
database	25	ZINC05519407	0
database	3879	ZINC06135996	0
database	1096	ZINC08440687	0
database	6819	ZINC08400808	0
database	6305	ZINC00985843	0
database	7161	ZINC02135508	0
database	6364	ZINC15989997	0
database	5605	ZINC08417639	0
database	4346	ZINC00703575	0
database	2858	ZINC00646442	0
database	1886	ZINC00627488	0

database	3675	ZINC09301976	0
database	2785	ZINC00646484	0
database	4873	ZINC08427656	0
database	1699	ZINC08439340	0
database	7118	ZINC08400445	0
database	5931	ZINC08413446	0
database	3954	ZINC00710233	0
database	3393	ZINC08435492	0
database	1736	ZINC08439284	0
database	231	ZINC00803814	0
database	257	ZINC00633758	0
database	4333	ZINC00845173	0
database	6861	ZINC00686722	0
database	2969	ZINC00645575	0
database	262	ZINC08442010	0
database	3155	ZINC00907393	0
database	2855	ZINC00646445	0
database	994	ZINC00070425	0
database	3140	ZINC02072174	0
database	3844	ZINC01801782	0
database	2640	ZINC08437674	0
database	6375	ZINC00706542	0
database	4612	ZINC08430434	0
database	5140	ZINC08426556	0
database	1854	ZINC08438972	0
database	244	ZINC08442028	0
database	6627	ZINC00219613	0

database	6791	ZINC00687339	0
database	4751	ZINC08430164	0
database	1964	ZINC02165282	0
database	1245	ZINC08440331	0
database	4379	ZINC00703533	0
database	4289	ZINC00997681	0
database	1083	ZINC08440704	0
database	505	ZINC00971827	0
database	5088	ZINC08426781	0
database	4123	ZINC00872119	0
database	6785	ZINC00687348	0
database	6016	ZINC08413110	0
database	112	ZINC08442108	0
database	909	ZINC06144685	0
database	6079	ZINC00688123	0
database	5656	ZINC08416809	0
database	975	ZINC02063421	0
database	5960	ZINC08413403	0
database	2467	ZINC00660707	0
database	155	ZINC08442082	0
database	5372	ZINC06177314	0
database	707	ZINC08441455	0
database	312	ZINC08441970	0
database	1457	ZINC00717892	0
database	3259	ZINC05359859	0
database	5311	ZINC08425805	0
database	470	ZINC00881560	0

database	3702	ZINC01802994	0
database	420	ZINC00881863	0
database	6172	ZINC08411410	0
database	1161	ZINC00702621	0
database	4248	ZINC00706748	0
database	5376	ZINC00293212	0
database	41	ZINC08442278	0
database	5765	ZINC08414490	0
database	6467	ZINC00035805	0
database	4057	ZINC08432460	0
database	1475	ZINC08439794	0
database	4048	ZINC08432481	0
database	3043	ZINC00645295	0
database	3553	ZINC08434998	0
database	7052	ZINC02777284	0
database	15	ZINC19990034	0
database	6169	ZINC08411415	0
database	5074	ZINC08426799	0
database	5431	ZINC08425332	0
database	2327	ZINC08438566	0
database	3970	ZINC08432723	0
database	1330	ZINC08738116	0
database	6638	ZINC08400938	0
database	5682	ZINC08415491	0
database	3610	ZINC01511146	0
database	5934	ZINC08413446	0
database	1164	ZINC00666441	0

database	841	ZINC00851289	0
database	2839	ZINC08437150	0
database	81	ZINC00633925	0
database	6534	ZINC02753981	0
database	6652	ZINC00687673	0
database	4666	ZINC00665550	0
database	6933	ZINC00686326	0
database	6357	ZINC00984804	0
database	3963	ZINC08432760	0
database	5150	ZINC08426456	0
database	1334	ZINC08440166	0
database	5134	ZINC03671790	0
database	718	ZINC15952848	0
database	2939	ZINC00645584	0
database	6325	ZINC00985620	0
database	5435	ZINC08425268	0
database	2150	ZINC01413496	0
database	1838	ZINC08439066	0
database	956	ZINC08440954	0
database	4188	ZINC00706922	0
database	3408	ZINC08435456	0
database	2722	ZINC08437433	0
database	4775	ZINC02061581	0
database	4269	ZINC00984954	0
database	5501	ZINC00707572	0
database	3556	ZINC00675997	0
database	3524	ZINC08435046	0

database	6573	ZINC00031404	0
database	3651	ZINC08433396	0
database	6919	ZINC01008440	0
database	2835	ZINC01890479	0
database	1582	ZINC08439613	0
database	3859	ZINC03243098	0
database	2427	ZINC08438245	0
database	4733	ZINC08430182	0
database	6744	ZINC02146168	0
database	4214	ZINC02083158	0
database	6490	ZINC08405578	0
database	4015	ZINC00986100	0
database	5002	ZINC02290799	0
database	1878	ZINC00627694	0
database	1523	ZINC02171063	0
database	2942	ZINC00645585	0
database	6873	ZINC00686681	0
database	4439	ZINC00997285	0
database	5186	ZINC08426324	0
database	5762	ZINC08414555	0
database	5644	ZINC08417208	0
database	2423	ZINC08829718	0
database	1402	ZINC08439979	0
database	4471	ZINC00292424	0
database	3705	ZINC02107105	0
database	4580	ZINC00845234	0
database	4837	ZINC08429974	0

database	5266	ZINC00708683	0
database	5587	ZINC08417896	0
database	5038	ZINC08426910	0
database	1932	ZINC00627341	0
database	3672	ZINC08433300	0
database	3256	ZINC06406689	0
database	1490	ZINC00645911	0
database	1434	ZINC08439920	0
database	6562	ZINC19791667	0
database	5780	ZINC08414189	0
database	6472	ZINC00705789	0
database	6688	ZINC08400828	0
database	5744	ZINC08414838	0
database	2631	ZINC01011073	0
database	3798	ZINC06144470	0
database	2901	ZINC09186823	0
database	6701	ZINC00687526	0
database	5178	ZINC00678144	0
database	7129	ZINC01008108	0
database	3027	ZINC00645507	0
database	7034	ZINC00999324	0
database	4279	ZINC00997713	0
database	3318	ZINC06177078	0
database	2054	ZINC01414773	0
database	3868	ZINC00078802	0
database	4564	ZINC02140776	0
database	674	ZINC00858026	0

database	4645	ZINC00983250	0
database	6966	ZINC08400654	0
database	4009	ZINC02889005	0
database	334	ZINC08441933	0
database	670	ZINC06406737	0
database	3023	ZINC00645510	0
database	6341	ZINC00985561	0
database	5418	ZINC00729165	0
database	4656	ZINC08430316	0
database	1223	ZINC00702507	0
database	5512	ZINC06444874	0
database	2256	ZINC08438693	0
database	577	ZINC08441626	0
database	107	ZINC08442110	0
database	496	ZINC00703120	0
database	2590	ZINC02161875	0
database	446	ZINC08441864	0
database	6837	ZINC08400793	0
database	135	ZINC00664736	0
database	973	ZINC00851794	0
database	2746	ZINC00646592	0
database	2710	ZINC08437467	0
database	4105	ZINC00985738	0
database	1138	ZINC08440596	0
database	2490	ZINC00660447	0
database	6338	ZINC00985575	0
database	901	ZINC08996454	0

database	6307	ZINC00985844	0
database	1943	ZINC08438875	0
database	1428	ZINC00843796	0
database	4353	ZINC00997533	0
database	2457	ZINC00624438	0
database	2235	ZINC00626508	0
database	5383	ZINC08425508	0
database	5267	ZINC08426023	0
database	3064	ZINC00877851	0
database	747	ZINC00856396	0
database	6292	ZINC00789915	0
database	5776	ZINC00689415	0
database	3918	ZINC02190790	0
database	3674	ZINC09301976	0
database	6080	ZINC00688122	0
database	598	ZINC00331109	0
database	1713	ZINC06270540	0
database	7002	ZINC01008228	0
database	3025	ZINC00645511	0
database	1373	ZINC02779333	0
database	5634	ZINC00692296	0
database	3281	ZINC00374021	0
database	1134	ZINC08440596	0
database	4842	ZINC08429973	0
database	6285	ZINC00792716	0
database	5775	ZINC08414433	0
database	6157	ZINC08411433	0

database	5829	ZINC08413954	0
database	1269	ZINC08440218	0
database	5867	ZINC08413888	0
database	242	ZINC08442028	0
database	4772	ZINC00664783	0
database	4620	ZINC08430390	0
database	1997	ZINC01011831	0
database	3262	ZINC00862462	0
database	2647	ZINC08437665	0
database	5962	ZINC08413403	0
database	5378	ZINC02573295	0
database	6824	ZINC00687237	0
database	4496	ZINC16916875	0
database	6885	ZINC00686605	0
database	992	ZINC08440840	0
database	3258	ZINC00625459	0
database	5036	ZINC08426912	0
database	5421	ZINC06279984	0
database	3062	ZINC00877863	0
database	4655	ZINC08430323	0
database	6419	ZINC08407214	0
database	3498	ZINC08435169	0
database	4598	ZINC08430538	0
database	4086	ZINC08432343	0
database	4268	ZINC00984954	0
database	2787	ZINC00646485	0
database	4454	ZINC09065134	0

database	1927	ZINC00627353	0
database	511	ZINC00881507	0
database	6133	ZINC08411476	0
database	2886	ZINC08437078	0
database	1970	ZINC02165246	0
database	6628	ZINC08402092	0
database	1383	ZINC00702350	0
database	3810	ZINC00723827	0
database	6369	ZINC08409920	0
database	168	ZINC05918663	0
database	7156	ZINC08400397	0
database	344	ZINC08441926	0
database	6068	ZINC00688318	0
database	3352	ZINC00975930	0
database	6949	ZINC08400672	0
database	4787	ZINC08430121	0
database	3350	ZINC00975932	0
database	5907	ZINC08413556	0
database	6656	ZINC00687669	0
database	2282	ZINC08438677	0
database	1260	ZINC08440226	0
database	6391	ZINC08407947	0
database	5236	ZINC02060713	0
database	4932	ZINC00711860	0
database	6830	ZINC02144099	0
database	4640	ZINC08430353	0
database	2016	ZINC09374642	0

database	2878	ZINC00646260	0
database	6497	ZINC19369493	0
database	406	ZINC00881954	0
database	3384	ZINC08435513	0
database	5192	ZINC00641029	0
database	6181	ZINC08411403	0
database	4569	ZINC02073196	0
database	1226	ZINC05944355	0
database	6442	ZINC18043169	0
database	4948	ZINC00711757	0
database	4261	ZINC00845119	0
database	1934	ZINC00627343	0
database	2430	ZINC00669316	0
database	5940	ZINC08413440	0
database	929	ZINC00859938	0
database	6978	ZINC08400629	0
database	3875	ZINC01738090	0
database	2967	ZINC00645574	0
database	3654	ZINC09045651	0
database	7119	ZINC08400443	0
database	6384	ZINC08407981	0
database	6121	ZINC08411515	0
database	6407	ZINC08407866	0
database	7025	ZINC02141503	0
database	2244	ZINC06445949	0
database	952	ZINC06300227	0
database	3835	ZINC00632130	0

database	3037	ZINC00144565	0
database	987	ZINC00702762	0
database	6067	ZINC00688318	0
database	1951	ZINC08438863	0
database	2845	ZINC08437142	0
database	5784	ZINC08414158	0
database	3220	ZINC00862964	0
database	1326	ZINC17160090	0
database	111	ZINC08442108	0
database	4714	ZINC00665093	0
database	2460	ZINC00624425	0
database	286	ZINC08836443	0
database	1390	ZINC08439999	0
database	143	ZINC00664717	0
database	5102	ZINC08426768	0
database	4781	ZINC00664762	0
database	3830	ZINC00719400	0
database	3086	ZINC00884272	0
database	2057	ZINC01414774	0
database	5041	ZINC08426875	0
database	2875	ZINC08437116	0
database	4411	ZINC08431358	0
database	5742	ZINC08414848	0
database	4244	ZINC02680120	0
database	527	ZINC15952846	0
database	2956	ZINC06144543	0
database	1755	ZINC00715097	0

database	4012	ZINC00669742	0
database	3107	ZINC00887444	0
database	4748	ZINC00996120	0
database	4106	ZINC00985737	0
database	2773	ZINC00646551	0
database	6191	ZINC08411350	0
database	3482	ZINC00196127	0
database	327	ZINC08441947	0
database	5239	ZINC00708739	0
database	474	ZINC00881548	0
database	6046	ZINC08413066	0
database	4521	ZINC08430843	0
database	3191	ZINC00907251	0
database	759	ZINC01019935	0
database	693	ZINC08441470	0
database	1344	ZINC08440140	0
database	4829	ZINC06287683	0
database	2713	ZINC00646623	0
database	2284	ZINC08438676	0
database	5794	ZINC08413985	0
database	1703	ZINC08439331	0
database	3363	ZINC00047417	0
database	2476	ZINC02163138	0
database	3240	ZINC00976114	0
database	4548	ZINC13941789	0
database	1491	ZINC00645908	0
database	1006	ZINC00702734	0

database	3895	ZINC00715260	0
database	5522	ZINC00627292	0
database	4556	ZINC08430666	0
database	3038	ZINC00144910	0
database	3357	ZINC08435556	0
database	3185	ZINC00907213	0
database	117	ZINC08442100	0
database	2840	ZINC08437150	0
database	2025	ZINC01011780	0
database	1200	ZINC02062354	0
database	819	ZINC08897840	0
database	2330	ZINC08438565	0
database	737	ZINC00703051	0
database	5392	ZINC00729269	0
database	3366	ZINC09332279	0
database	104	ZINC01102227	0
database	7076	ZINC08400479	0
database	4467	ZINC00984330	0
database	3658	ZINC09045647	0
database	2980	ZINC00645568	0
database	2117	ZINC01414764	0
database	5034	ZINC02990250	0
database	4034	ZINC00669557	0
database	2783	ZINC00646487	0
database	5290	ZINC02060588	0
database	2601	ZINC02161821	0
database	7036	ZINC00999322	0

database	4729	ZINC06287766	0
database	1126	ZINC08440656	0
database	6682	ZINC08400846	0
database	5343	ZINC02059571	0
database	169	ZINC05918661	0
database	966	ZINC06445739	0
database	3328	ZINC06156995	0
database	1909	ZINC08438901	0
database	4199	ZINC02084704	0
database	963	ZINC06445857	0
database	2264	ZINC01019496	0
database	1035	ZINC00702716	0
database	616	ZINC08441598	0
database	6632	ZINC00687887	0
database	4450	ZINC09065134	0
database	1992	ZINC01011832	0
database	1328	ZINC17160090	0
database	688	ZINC08441471	0
database	6703	ZINC00687518	0
database	6256	ZINC00987215	0
database	4171	ZINC00706992	0
database	1868	ZINC02167981	0
database	1578	ZINC08439614	0
database	6858	ZINC00686736	0
database	4855	ZINC08429927	0
database	4850	ZINC00982640	0
database	5998	ZINC08413134	0

database	3605	ZINC03057195	0
database	7101	ZINC08400465	0
database	5223	ZINC00969727	0
database	4863	ZINC00845183	0
database	4673	ZINC08430286	0
database	5985	ZINC08413236	0
database	4236	ZINC09012357	0
database	5778	ZINC08414278	0
database	395	ZINC02064383	0
database	6110	ZINC08411532	0
database	5713	ZINC08415472	0
database	2523	ZINC00623810	0
database	2388	ZINC08438456	0
database	5496	ZINC00345164	0
database	4753	ZINC00729387	0
database	3579	ZINC08434910	0
database	3348	ZINC02071979	0
database	2531	ZINC00623719	0
database	1689	ZINC08439355	0
database	958	ZINC08440929	0
database	6802	ZINC00687294	0
database	4359	ZINC00703556	0
database	3447	ZINC00662898	0
database	2246	ZINC06445928	0
database	27	ZINC06219168	0
database	6633	ZINC00687881	0
database	6945	ZINC08400677	0

database	5513	ZINC00272824	0
database	3138	ZINC08435637	0
database	2896	ZINC08903739	0
database	3126	ZINC00787054	0
database	1928	ZINC00627356	0
database	549	ZINC00703106	0
database	7150	ZINC00675662	0
database	6244	ZINC00987605	0
database	2996	ZINC00645562	0
database	3550	ZINC00201631	0
database	6378	ZINC08408021	0
database	5932	ZINC08413446	0
database	2379	ZINC08438514	0
database	21	ZINC05286115	0
database	1191	ZINC08440509	0
database	826	ZINC08897821	0
database	7037	ZINC00999317	0
database	4923	ZINC00855950	0
database	4324	ZINC02179067	0
database	1810	ZINC02169126	0
database	202	ZINC08442069	0
database	2462	ZINC00660886	0
database	4800	ZINC00664617	0
database	5294	ZINC08425981	0
database	2214	ZINC08438705	0
database	6404	ZINC08407877	0
database	4949	ZINC00711748	0

database	3894	ZINC08432903	0
database	2098	ZINC01011741	0
database	425	ZINC00881842	0
database	4446	ZINC08431227	0
database	1420	ZINC00471779	0
database	1778	ZINC08439206	0
database	444	ZINC06143751	0
database	6658	ZINC00687668	0
database	414	ZINC08441880	0
database	4429	ZINC08431314	0
database	7162	ZINC02135498	0
database	1586	ZINC08439611	0
database	6773	ZINC00687377	0
database	6279	ZINC04168845	0
database	1716	ZINC08439310	0
database	1885	ZINC00627483	0
database	6517	ZINC08405156	0
database	3178	ZINC06444286	0
database	3777	ZINC02106667	0
database	3602	ZINC08434845	0
database	5631	ZINC01010573	0
database	2359	ZINC00625740	0
database	254	ZINC08442020	0
database	6345	ZINC00985555	0
database	6052	ZINC08413061	0
database	2410	ZINC00624709	0
database	5027	ZINC00796322	0

database	2623	ZINC01011081	0
database	6962	ZINC01008277	0
database	4910	ZINC08427620	0
database	2382	ZINC08438496	0
database	928	ZINC08441016	0
database	6381	ZINC08408007	0
database	1809	ZINC02169133	0
database	1162	ZINC00844490	0
database	6708	ZINC00687495	0
database	3477	ZINC00655501	0
database	6232	ZINC15985639	0
database	2614	ZINC08437694	0
database	7191	ZINC06142731	0
database	102	ZINC08442112	0
database	6675	ZINC00687615	0
database	4703	ZINC00665233	0
database	3567	ZINC00675849	0
database	34	ZINC18141403	0
database	6587	ZINC05316172	0
database	1264	ZINC08440218	0
database	4615	ZINC00996342	0
database	1343	ZINC08440140	0
database	6137	ZINC08411466	0
database	810	ZINC08914319	0
database	556	ZINC00878058	0
database	4854	ZINC00729008	0
database	642	ZINC15885023	0

database	4135	ZINC08432199	0
database	4502	ZINC00983949	0
database	6979	ZINC00677023	0
database	6151	ZINC08411444	0
database	6059	ZINC08413048	0
database	4628	ZINC08430361	0
database	3171	ZINC00915459	0
database	3265	ZINC00625896	0
database	18	ZINC01240782	0
database	3105	ZINC00883775	0
database	1337	ZINC06194705	0
database	6103	ZINC08411597	0
database	2383	ZINC08690308	0
database	1772	ZINC01013096	0
database	14	ZINC05556455	0
database	753	ZINC08441400	0
database	4885	ZINC08427631	0
database	2836	ZINC01890479	0
database	1249	ZINC08440257	0
database	5443	ZINC00707858	0
database	2864	ZINC00646443	0
database	6210	ZINC04473388	0
database	6277	ZINC04168846	0
database	3336	ZINC08435574	0
database	1926	ZINC00627350	0
database	96	ZINC08442117	0
database	3372	ZINC00872940	0

database	1571	ZINC00624874	0
database	5143	ZINC02281994	0
database	4608	ZINC00983556	0
database	2629	ZINC00622878	0
database	4088	ZINC08432324	0
database	5912	ZINC08413520	0
database	2294	ZINC08438658	0
database	6001	ZINC08413118	0
database	5939	ZINC08413440	0
database	1380	ZINC00702349	0
database	3552	ZINC02069119	0
database	3418	ZINC02071234	0
database	1449	ZINC09041905	0
database	2300	ZINC08438654	0
database	1798	ZINC02169522	0
database	6039	ZINC08413079	0
database	1945	ZINC00627108	0
database	98	ZINC08442117	0
database	4068	ZINC00710201	0
database	412	ZINC01126045	0
database	6879	ZINC00686658	0
database	6553	ZINC02181556	0
database	4296	ZINC00845633	0
database	5936	ZINC08413440	0
database	2018	ZINC01011788	0
database	4225	ZINC06154143	0
database	5008	ZINC08427134	0

database	1512	ZINC13545838	0
database	1842	ZINC02168673	0
database	6287	ZINC08410677	0
database	5514	ZINC00728386	0
database	6618	ZINC19370782	0
database	862	ZINC00852503	0
database	5951	ZINC08413411	0
database	5325	ZINC00647559	0
database	2903	ZINC00693848	0
database	5508	ZINC08424829	0
database	2716	ZINC00646599	0
database	6549	ZINC01022566	0
database	4856	ZINC00729009	0
database	3302	ZINC02967463	0
database	4958	ZINC00711715	0
database	4672	ZINC00479469	0
database	1252	ZINC08440246	0
database	1220	ZINC08440404	0
database	6171	ZINC08411415	0
database	3812	ZINC08433098	0
database	3946	ZINC01896133	0
database	1231	ZINC06159425	0
database	6752	ZINC00687407	0
database	2950	ZINC06144561	0
database	2189	ZINC01413499	0
database	2931	ZINC01795810	0
database	1307	ZINC08440200	0

database	3707	ZINC06444249	0
database	844	ZINC08441234	0
database	6342	ZINC00985562	0
database	337	ZINC08441932	0
database	796	ZINC00853499	0
database	7206	ZINC08400272	0
database	4919	ZINC03079308	0
database	5382	ZINC08425528	0
database	5144	ZINC00709589	0
database	5284	ZINC00661030	0
database	2279	ZINC08438679	0
database	721	ZINC01019938	0
database	5655	ZINC08416870	0
database	3851	ZINC06154170	0
database	4865	ZINC09110318	0
database	1012	ZINC00702730	0
database	6817	ZINC02144132	0
database	2763	ZINC02158924	0
database	808	ZINC13569448	0
database	6178	ZINC08411403	0
database	4409	ZINC02084048	0
database	2822	ZINC08437172	0
database	2364	ZINC09046651	0
database	5423	ZINC08425370	0
database	2930	ZINC01795810	0
database	3148	ZINC08435628	0
database	43	ZINC08442277	0

database	5062	ZINC08426813	0
database	273	ZINC08442004	0
database	1745	ZINC01013156	0
database	5170	ZINC08426413	0
database	7185	ZINC08400311	0
database	235	ZINC08442040	0
database	7211	ZINC08400241	0
database	5405	ZINC00199681	0
database	6736	ZINC00687428	0
database	3045	ZINC00386177	0
database	4125	ZINC00985530	0
database	6585	ZINC00827369	0
database	5424	ZINC08425368	0
database	3755	ZINC08742400	0
database	2175	ZINC01413495	0
database	309	ZINC06446605	0
database	6010	ZINC08413115	0
database	3446	ZINC08435335	0
database	3772	ZINC02679262	0
database	6671	ZINC00687625	0
database	5955	ZINC08413407	0
database	1054	ZINC00702700	0
database	6112	ZINC08411532	0
database	95	ZINC08442118	0
database	610	ZINC15880060	0
database	179	ZINC00664546	0
database	6778	ZINC00687361	0

database	6247	ZINC08410936	0
database	3900	ZINC01895602	0
database	3463	ZINC08435278	0
database	5654	ZINC08416870	0
database	799	ZINC00702990	0
database	418	ZINC01126049	0
database	673	ZINC08914574	0
database	2491	ZINC00660442	0
database	5958	ZINC08413403	0
database	1771	ZINC01013124	0
database	2229	ZINC00626525	0
database	3122	ZINC00862148	0
database	1276	ZINC00702446	0
database	795	ZINC08441311	0
database	6571	ZINC02726205	0
database	4592	ZINC00665995	0
database	5402	ZINC01024808	0
database	905	ZINC08441041	0
database	4205	ZINC02083162	0
database	3661	ZINC00730338	0
database	2578	ZINC08437722	0
database	448	ZINC00881655	0
database	6662	ZINC00687664	0
database	6063	ZINC00688321	0
database	4970	ZINC08427418	0
database	4533	ZINC08430801	0
database	5771	ZINC06215466	0

database	9	ZINC19799526	0
database	4498	ZINC08430964	0
database	1783	ZINC08439187	0
database	2912	ZINC00693771	0
database	5299	ZINC08425969	0
database	5371	ZINC06177524	0
database	5957	ZINC08413407	0
database	3530	ZINC01953697	0
database	6776	ZINC00687363	0
database	489	ZINC00650712	0
database	4755	ZINC00729390	0
database	2883	ZINC08437082	0
database	5536	ZINC03666983	0
database	187	ZINC04167843	0
database	153	ZINC08442083	0
database	739	ZINC00703051	0
database	4071	ZINC00669290	0
database	6056	ZINC08413061	0
database	4792	ZINC00664668	0
database	2489	ZINC02162861	0
database	2210	ZINC00626750	0
database	1141	ZINC08440596	0
database	6268	ZINC00987156	0
database	5630	ZINC01010573	0
database	423	ZINC00881851	0
database	2634	ZINC08437680	0
database	4154	ZINC00707027	0

database	2725	ZINC08437431	0
database	3036	ZINC08436894	0
database	7006	ZINC01008218	0
database	3757	ZINC08742399	0
database	714	ZINC15884978	0
database	6258	ZINC00987205	0
database	6205	ZINC08411305	0
database	3383	ZINC00352940	0
database	2948	ZINC06144561	0
database	1871	ZINC00577331	0
database	4959	ZINC00711715	0
database	429	ZINC08441879	0
database	3560	ZINC08434961	0
database	1565	ZINC08439647	0
database	5623	ZINC08417541	0
database	5803	ZINC08413985	0
database	6315	ZINC00985810	0
database	6160	ZINC08411428	0
database	6134	ZINC08411472	0
database	3938	ZINC08432834	0
database	2958	ZINC00645581	0
database	2738	ZINC08437422	0
database	4738	ZINC02796672	0
database	3740	ZINC01017590	0
database	6382	ZINC08408004	0
database	2000	ZINC02165050	0
database	3378	ZINC08435522	0

database	6665	ZINC08400869	0
database	5414	ZINC00729178	0
database	1559	ZINC00387112	0
database	770	ZINC00703045	0
database	6011	ZINC08413115	0
database	4413	ZINC08431356	0
database	4416	ZINC08431354	0
database	67	ZINC08442186	0
database	2784	ZINC00646484	0
database	4686	ZINC02739071	0
database	2393	ZINC00624976	0
database	2985	ZINC13122227	0
database	1253	ZINC08440243	0
database	465	ZINC00881571	0
database	2437	ZINC00624580	0
database	6863	ZINC00686707	0
database	1581	ZINC08439613	0
database	4853	ZINC02082221	0
database	1938	ZINC08438886	0
database	1290	ZINC08440205	0
database	2605	ZINC00623039	0
database	6410	ZINC08407632	0
database	2803	ZINC08437215	0
database	5145	ZINC00709589	0
database	2068	ZINC01414783	0
database	2538	ZINC00623503	0
database	7183	ZINC08400316	0

database	2065	ZINC01413469	0
database	3063	ZINC00676399	0
database	2152	ZINC02164636	0
database	3838	ZINC02680175	0
database	4946	ZINC01029906	0
database	1686	ZINC08439364	0
database	515	ZINC00881493	0
database	6081	ZINC08411715	0
database	2253	ZINC06445943	0
database	5252	ZINC00780342	0
database	6910	ZINC02830669	0
database	3451	ZINC08435307	0
database	5708	ZINC08415472	0
database	2380	ZINC08438506	0
database	1423	ZINC00702307	0
database	6138	ZINC08411466	0
database	5510	ZINC00304444	0
database	3368	ZINC09044725	0
database	5509	ZINC00155291	0
database	3054	ZINC08436820	0
database	2978	ZINC00645568	0
database	2223	ZINC00626544	0
database	6077	ZINC01009832	0
database	4148	ZINC02281623	0
database	2121	ZINC01413446	0
database	1898	ZINC00627444	0
database	4663	ZINC00996267	0

database	6704	ZINC00687508	0
database	6383	ZINC08407985	0
database	6246	ZINC08410939	0
database	1757	ZINC00715098	0
database	4607	ZINC08430447	0
database	3211	ZINC00867612	0
database	5854	ZINC08413917	0
database	6686	ZINC02842347	0
database	1944	ZINC00627106	0
database	4636	ZINC08430354	0
database	6698	ZINC00687547	0
database	382	ZINC02064397	0
database	6324	ZINC00985620	0
database	4035	ZINC00628478	0
database	6471	ZINC00705788	0
database	6619	ZINC08402790	0
database	634	ZINC00861338	0
database	4390	ZINC02083070	0
database	3984	ZINC02085870	0
database	3308	ZINC08817702	0
database	1679	ZINC08439378	0
database	5	ZINC12378847	0
database	4285	ZINC02742550	0
database	6179	ZINC08411403	0
database	5737	ZINC08414961	0
database	2426	ZINC00669365	0
database	5141	ZINC00969854	0

database	3535	ZINC02069275	0
database	6198	ZINC08411315	0
database	2641	ZINC08437672	0
database	6950	ZINC00919870	0
database	1263	ZINC00970507	0
database	5385	ZINC08425496	0
database	4393	ZINC04482653	0
database	6779	ZINC00687360	0
database	33	ZINC18141403	0
database	6281	ZINC06300277	0
database	6696	ZINC00687556	0
database	6766	ZINC00687394	0
database	4104	ZINC08432294	0
database	4819	ZINC08430041	0
database	5919	ZINC08413517	0
database	6255	ZINC00987222	0
database	4952	ZINC00711748	0
database	2083	ZINC01414786	0
database	302	ZINC08441976	0
database	839	ZINC00851613	0
database	6474	ZINC01022828	0
database	6054	ZINC08413061	0
database	2183	ZINC08438763	0
database	3199	ZINC00877588	0
database	72	ZINC08442174	0
database	1584	ZINC08439611	0
database	2692	ZINC06147720	0

database	7074	ZINC08400481	0
database	5984	ZINC08413236	0
database	4534	ZINC08430796	0
database	734	ZINC00703050	0
database	3801	ZINC00723830	0
database	5642	ZINC08417216	0
database	2827	ZINC05360325	0
database	5374	ZINC00292755	0
database	6952	ZINC00919872	0
database	6894	ZINC00686586	0
database	2842	ZINC08437147	0
database	2714	ZINC00646623	0
database	30	ZINC18141403	0
database	6987	ZINC00677001	0
database	6450	ZINC00705820	0
database	5191	ZINC08426291	0
database	5506	ZINC08424837	0
database	3803	ZINC00723825	0
database	3382	ZINC00975843	0
database	6482	ZINC19791930	0
database	6225	ZINC08411232	0
database	4130	ZINC01004581	0
database	4505	ZINC08430938	0
database	3945	ZINC02085982	0
database	4395	ZINC08431434	0
database	3518	ZINC02069936	0
database	1217	ZINC08440410	0

database	6657	ZINC02146229	0
database	2686	ZINC08437519	0
database	6	ZINC03901268	0
database	4649	ZINC00983246	0
database	3645	ZINC00702272	0
database	3288	ZINC20032159	0
database	6331	ZINC00985593	0
database	4979	ZINC00711393	0
database	976	ZINC00702759	0
database	3753	ZINC09301883	0
database	181	ZINC00664549	0
database	1728	ZINC08439293	0
database	7084	ZINC02252325	0
database	2073	ZINC01414784	0
database	3729	ZINC01830761	0
database	2778	ZINC00646486	0
database	5211	ZINC08426213	0
database	2231	ZINC00626535	0
database	3113	ZINC00891476	0
database	6309	ZINC00985825	0
database	6214	ZINC04473387	0
database	6754	ZINC00687405	0
database	4993	ZINC08427304	0
database	2579	ZINC01011161	0
database	6479	ZINC12042477	0
database	2824	ZINC05360331	0
database	1588	ZINC08439602	0

database	2072	ZINC01011762	0
database	2124	ZINC01413447	0
database	2050	ZINC01414766	0
database	2118	ZINC01414764	0
database	5412	ZINC00729200	0
database	6476	ZINC08405981	0
database	4278	ZINC01003571	0
database	5795	ZINC08413985	0
database	4736	ZINC08430174	0
database	1628	ZINC08439527	0
database	333	ZINC08441933	0
database	166	ZINC00633883	0
database	5925	ZINC08413491	0
database	4667	ZINC00665548	0
database	4463	ZINC08431176	0
database	3416	ZINC00827958	0
database	1331	ZINC08738116	0
database	1087	ZINC00702690	0
database	887	ZINC05520252	0
database	2944	ZINC00645585	0
database	4288	ZINC00845725	0
database	3980	ZINC00710221	0
database	6559	ZINC12411205	0
database	2345	ZINC00625759	0
database	1177	ZINC00702596	0
database	149	ZINC00664709	0
database	3585	ZINC02068744	0

database	6855	ZINC00686751	0
database	6774	ZINC00687376	0
database	1394	ZINC15906697	0
database	10	ZINC00982962	0
database	2134	ZINC08438771	0
database	5373	ZINC00822873	0
database	4250	ZINC00984975	0
database	2941	ZINC00645584	0
database	6417	ZINC08407217	0
database	4670	ZINC08430298	0
database	1370	ZINC08440095	0
database	5011	ZINC18116401	0
database	7226	ZINC02101687	0
database	6075	ZINC08412373	0
database	2519	ZINC00623963	0
database	4412	ZINC00997381	0
database	4122	ZINC00985541	0
database	6506	ZINC19369484	0
database	3877	ZINC08432972	0
database	1887	ZINC02167569	0
database	4420	ZINC00997359	0
database	4677	ZINC00845120	0
database	2866	ZINC00646443	0
database	1567	ZINC08439634	0
database	6022	ZINC08413106	0
database	5061	ZINC08426813	0
database	1510	ZINC04112603	0

database	5032	ZINC08426976	0
database	6532	ZINC19370921	0
database	6316	ZINC00985634	0
database	89	ZINC08442121	0
database	5992	ZINC08413182	0
database	4828	ZINC09110716	0
database	665	ZINC06406305	0
database	5449	ZINC08425136	0
database	4474	ZINC08431087	0
database	6920	ZINC01008438	0
database	4699	ZINC00845206	0
database	1140	ZINC08440596	0
database	4332	ZINC16677185	0
database	4054	ZINC08432462	0
database	4937	ZINC00711814	0
database	3022	ZINC00645510	0
database	5657	ZINC00691789	0
database	5282	ZINC00661027	0
database	3956	ZINC00710232	0
database	970	ZINC08440874	0
database	2894	ZINC00693894	0
database	2816	ZINC00832608	0
database	892	ZINC00850364	0
database	5675	ZINC08415497	0
database	4083	ZINC00985787	0
database	3808	ZINC00723827	0
database	4251	ZINC00984976	0

database	7115	ZINC00676553	0
database	3754	ZINC08742400	0
database	4544	ZINC13893793	0
database	3540	ZINC00677054	0
database	2335	ZINC08438562	0
database	1761	ZINC01013140	0
database	4060	ZINC02085493	0
database	5799	ZINC08413985	0
database	2469	ZINC08438082	0
database	5434	ZINC08425269	0
database	4740	ZINC06287778	0
database	3985	ZINC00986282	0
database	1942	ZINC08438876	0
database	2288	ZINC08438674	0
database	4046	ZINC08432487	0
database	7190	ZINC06142731	0
database	4295	ZINC00845632	0
database	54	ZINC08442218	0
database	5004	ZINC08427165	0
database	1335	ZINC08440165	0
database	4211	ZINC00628390	0
database	7117	ZINC00999218	0
database	3146	ZINC05943324	0
database	3737	ZINC08433174	0
database	3618	ZINC00808997	0
database	7202	ZINC13111995	0
database	5873	ZINC08413885	0

database	6074	ZINC08412453	0
database	2970	ZINC00645575	0
database	1672	ZINC08439383	0
database	1853	ZINC02168309	0
database	847	ZINC02380267	0
database	926	ZINC08441018	0
database	2474	ZINC01011448	0
database	6213	ZINC04473387	0
database	5532	ZINC08424582	0
database	1382	ZINC00702350	0
database	398	ZINC02064381	0
database	3170	ZINC00915470	0
database	2100	ZINC01413448	0
database	460	ZINC00881588	0
database	2852	ZINC06144743	0
database	1124	ZINC08440666	0
database	1526	ZINC00381911	0
database	3154	ZINC00907400	0
database	6420	ZINC08407214	0
database	5131	ZINC00277513	0
database	7065	ZINC02158027	0
database	5964	ZINC08413387	0
database	2090	ZINC01011746	0
database	4421	ZINC00730156	0
database	2240	ZINC06445930	0
database	2946	ZINC06144562	0
database	1940	ZINC08438879	0

database	1924	ZINC00627365	0
database	1802	ZINC08439153	0
database	2888	ZINC00646196	0
database	5810	ZINC08413980	0
database	7188	ZINC00668919	0
database	3024	ZINC00645511	0
database	668	ZINC06406308	0
database	6339	ZINC00985575	0
database	4074	ZINC08432398	0
database	3413	ZINC02071253	0
database	7038	ZINC00999316	0
database	487	ZINC00650986	0
database	6859	ZINC02143514	0
database	4507	ZINC00029091	0
database	5346	ZINC00297341	0
database	1020	ZINC00702727	0
database	2329	ZINC08438565	0
database	4302	ZINC00845573	0
database	4273	ZINC08431728	0
database	3917	ZINC08432883	0
database	4906	ZINC08427628	0
database	6049	ZINC08413066	0
database	2915	ZINC00693759	0
database	1675	ZINC08439380	0
database	6328	ZINC00985592	0
database	1724	ZINC06270551	0
database	1329	ZINC17160090	0

database	1287	ZINC00702437	0
database	5549	ZINC00707249	0
database	2992	ZINC00645565	0
database	1189	ZINC08440509	0
database	4451	ZINC09065134	0
database	5233	ZINC02475452	0
database	3166	ZINC00778417	0
database	2021	ZINC02164882	0
database	3793	ZINC08433113	0
database	3145	ZINC00907580	0
database	7021	ZINC00676722	0
database	6469	ZINC08406079	0
database	2829	ZINC00625693	0
database	6439	ZINC18043169	0
database	5729	ZINC08415087	0
database	1353	ZINC00142373	0
database	342	ZINC08441928	0
database	6666	ZINC00687633	0
database	3797	ZINC06144470	0
database	5561	ZINC08424410	0
database	6661	ZINC00687666	0
database	3648	ZINC08433413	0
database	3421	ZINC01808107	0
database	1355	ZINC01306294	0
database	5048	ZINC08426840	0
database	6975	ZINC06023577	0
database	5978	ZINC08413327	0

database	3228	ZINC06442944	0
database	5908	ZINC08413556	0
database	4799	ZINC02144365	0
database	3829	ZINC00719425	0
database	7126	ZINC00676402	0
database	1859	ZINC02484245	0
database	622	ZINC00237625	0
database	53	ZINC08442219	0
database	5594	ZINC08417758	0
database	5565	ZINC08424402	0
database	5116	ZINC08426701	0
database	3979	ZINC08432700	0
database	6737	ZINC00687427	0
database	5647	ZINC08417195	0
database	3990	ZINC08432677	0
database	1666	ZINC08439386	0
database	1302	ZINC00702428	0
database	6813	ZINC08400811	0
database	4998	ZINC08427239	0
database	2871	ZINC00646431	0
database	374	ZINC02064404	0
database	4812	ZINC00845126	0
database	5819	ZINC08413980	0
database	3873	ZINC08432979	0
database	619	ZINC08441595	0
database	3116	ZINC00862108	0
database	7165	ZINC02135480	0

database	5664	ZINC00691603	0
database	2759	ZINC00646579	0
database	2560	ZINC01011358	0
database	6044	ZINC08413066	0
database	397	ZINC02064381	0
database	6986	ZINC00677004	0
database	119	ZINC08442097	0
database	156	ZINC08442082	0
database	606	ZINC15880062	0
database	3821	ZINC00719595	0
database	5830	ZINC08413954	0
database	3699	ZINC09271700	0
database	5169	ZINC00642619	0
database	4252	ZINC00845114	0
database	3156	ZINC08435624	0
database	571	ZINC08441629	0
database	1091	ZINC00702692	0
database	4685	ZINC02739070	0
database	6248	ZINC00987518	0
database	1988	ZINC02165122	0
database	3913	ZINC00952959	0
database	1144	ZINC08440584	0
database	1968	ZINC02165256	0
database	2019	ZINC09123695	0
database	5369	ZINC06177530	0
database	2698	ZINC01891300	0
database	603	ZINC15880061	0

database	2709	ZINC00368131	0
database	6788	ZINC00687342	0
database	1316	ZINC00702427	0
database	4518	ZINC00036618	0
database	1127	ZINC08440654	0
database	2219	ZINC08438703	0
database	3314	ZINC06136516	0
database	7120	ZINC08400441	0
database	2911	ZINC02156222	0
database	3749	ZINC06144031	0
database	1561	ZINC08439649	0
database	1801	ZINC02169356	0
database	2849	ZINC06144743	0
database	6796	ZINC00687329	0
database	5613	ZINC01010660	0
database	6645	ZINC00687755	0
database	360	ZINC08441915	0
database	1188	ZINC08440510	0
database	2862	ZINC00646442	0
database	4134	ZINC08432200	0
database	4031	ZINC01896171	0
database	989	ZINC08440861	0
database	4530	ZINC08430810	0
database	319	ZINC08441965	0
database	4875	ZINC08427653	0
database	3948	ZINC00670847	0
database	174	ZINC00664571	0

database	6190	ZINC08411358	0
database	2873	ZINC00148710	0
database	3431	ZINC08435360	0
database	5349	ZINC00267454	0
database	7187	ZINC00668926	0
database	1858	ZINC08438965	0
database	4547	ZINC08430705	0
database	3132	ZINC00422351	0
database	2800	ZINC08437218	0
database	4063	ZINC00669353	0
database	1062	ZINC00702695	0
database	6227	ZINC08411187	0
database	3790	ZINC00723896	0
database	108	ZINC08442109	0
database	4936	ZINC00711827	0
database	5069	ZINC08426811	0
database	4127	ZINC08432204	0
database	7182	ZINC08400323	0
database	7099	ZINC08400465	0
database	4809	ZINC08430090	0
database	2307	ZINC01019486	0
database	243	ZINC08442028	0
database	2998	ZINC00645563	0
database	2365	ZINC00625470	0
database	5360	ZINC08425625	0
database	2086	ZINC01414786	0
database	417	ZINC01126049	0

database	3354	ZINC00878185	0
database	180	ZINC08442077	0
database	3910	ZINC02087895	0
database	4002	ZINC01895069	0
database	4763	ZINC00664827	0
database	4400	ZINC00703442	0
database	1737	ZINC01013164	0
database	6453	ZINC18189030	0
database	4099	ZINC08432296	0
database	1562	ZINC08439649	0
database	7181	ZINC08400334	0
database	3046	ZINC08436847	0
database	1863	ZINC02168095	0
database	2548	ZINC08437779	0
database	5158	ZINC02181246	0
database	6330	ZINC00985593	0
database	2422	ZINC00713531	0
database	6654	ZINC02146236	0
database	6604	ZINC08403215	0
database	5973	ZINC08413383	0
database	3516	ZINC08435058	0
database	5151	ZINC08426450	0
database	4794	ZINC00845116	0
database	6301	ZINC00985861	0
database	2751	ZINC02075274	0
database	4759	ZINC00664838	0
database	1454	ZINC08439892	0

database	2591	ZINC02161870	0
database	2339	ZINC00625750	0
database	1579	ZINC08439614	0
database	765	ZINC08441393	0
database	2527	ZINC00623786	0
database	4832	ZINC08429984	0
database	986	ZINC00702762	0
database	1725	ZINC06270551	0
database	4192	ZINC00997933	0
database	3134	ZINC00812971	0
database	1158	ZINC02063028	0
database	4452	ZINC09065134	0
database	2095	ZINC01414752	0
database	2023	ZINC08438777	0
database	5852	ZINC08413922	0
database	3818	ZINC00719628	0
database	6421	ZINC08407210	0
database	6122	ZINC08411510	0
database	1214	ZINC08440415	0
database	4590	ZINC00665992	0
database	5645	ZINC08417203	0
database	6845	ZINC00686821	0
database	4840	ZINC08429973	0
database	2909	ZINC00693796	0
database	1156	ZINC08440584	0
database	5101	ZINC08426768	0
database	4190	ZINC08431890	0

database	3788	ZINC01802677	0
database	2872	ZINC00646371	0
database	492	ZINC00703128	0
database	1192	ZINC08440509	0
database	6501	ZINC01022711	0
database	4895	ZINC08427629	0
database	5289	ZINC08426006	0
database	6564	ZINC19791652	0
database	5503	ZINC00854815	0
database	1706	ZINC08439329	0
database	3955	ZINC00710233	0
database	1742	ZINC02502128	0
database	5013	ZINC08427105	0
database	3243	ZINC05576958	0
database	1281	ZINC08440213	0
database	1564	ZINC08439647	0
database	4881	ZINC08427631	0
database	2870	ZINC00646431	0
database	4055	ZINC08432461	0
database	4552	ZINC08430698	0
database	4249	ZINC00706749	0
database	4760	ZINC00664835	0
database	6887	ZINC00686601	0
database	3730	ZINC00727868	0
database	3080	ZINC00884343	0
database	2480	ZINC08438059	0
database	225	ZINC00803806	0

database	3878	ZINC08432969	0
database	2764	ZINC00693970	0
database	437	ZINC08441875	0
database	6152	ZINC08411440	0
database	3845	ZINC01801779	0
database	392	ZINC08441894	0
database	4796	ZINC00729124	0
database	6864	ZINC00686706	0
database	2421	ZINC00624626	0
database	220	ZINC13161524	0
database	3188	ZINC00897337	0
database	5388	ZINC06446032	0
database	1636	ZINC04113088	0
database	3471	ZINC00655595	0
database	2001	ZINC01011827	0
database	3196	ZINC00976237	0
database	2029	ZINC01011777	0
database	1069	ZINC08440712	0
database	2648	ZINC08437664	0
database	1425	ZINC00702308	0
database	2736	ZINC00694090	0
database	4203	ZINC02744559	0
database	4356	ZINC00703555	0
database	1865	ZINC08438954	0
database	5975	ZINC08413383	0
database	5560	ZINC08424410	0
database	2042	ZINC01414772	0

database	2604	ZINC00623039	0
database	2496	ZINC00624284	0
database	3691	ZINC08433258	0
database	443	ZINC06143751	0
database	1607	ZINC00717217	0
database	6032	ZINC08413100	0
database	5848	ZINC08413924	0
database	4574	ZINC02740302	0
database	3515	ZINC08435059	0
database	1645	ZINC04113086	0
database	1753	ZINC02503421	0
database	376	ZINC08441904	0
database	4523	ZINC08430829	0
database	281	ZINC09122186	0
database	4207	ZINC02083161	0
database	1384	ZINC08440017	0
database	682	ZINC00648858	0
database	7116	ZINC02135812	0
database	3570	ZINC00675754	0
database	159	ZINC05921075	0
database	5743	ZINC08414848	0
database	1327	ZINC17160090	0
database	5231	ZINC02475452	0
database	2107	ZINC01414731	0
database	1445	ZINC09109830	0
database	763	ZINC00703043	0
database	5453	ZINC08425067	0

database	943	ZINC08440987	0
database	2670	ZINC04167655	0
database	5232	ZINC02475452	0
database	1224	ZINC00702507	0
database	1389	ZINC08440003	0
database	2727	ZINC00694093	0
database	6119	ZINC08411515	0
database	5292	ZINC08425984	0
database	6351	ZINC00846344	0
database	1662	ZINC08439396	0
database	5915	ZINC08413520	0
database	5847	ZINC08413924	0
database	4286	ZINC00984876	0
database	3068	ZINC06444403	0
database	5611	ZINC00692629	0
database	6742	ZINC00687422	0
database	6886	ZINC00686604	0
database	5598	ZINC00692949	0
database	4462	ZINC08431180	0
database	1228	ZINC00702500	0
database	1864	ZINC08438955	0
database	5129	ZINC06245044	0
database	5142	ZINC02060992	0
database	2924	ZINC04149943	0
database	592	ZINC08441621	0
database	1441	ZINC00721238	0
database	3100	ZINC00883918	0

database	5739	ZINC08414959	0
database	6489	ZINC00842067	0
database	1471	ZINC08439819	0
database	6745	ZINC00687420	0
database	4975	ZINC03114733	0
database	1655	ZINC00249053	0
database	3921	ZINC08432867	0
database	194	ZINC04167833	0
database	1107	ZINC00702673	0
database	4864	ZINC09110318	0
database	4924	ZINC02186610	0
database	1103	ZINC05918668	0
database	5303	ZINC08425948	0
database	3218	ZINC08435605	0
database	5586	ZINC08417896	0
database	3394	ZINC00975643	0
database	142	ZINC08442088	0
database	4899	ZINC08427629	0
database	6392	ZINC08407927	0
database	450	ZINC02889263	0
database	234	ZINC08442040	0
database	19	ZINC01240782	0
database	7171	ZINC02135395	0
database	6358	ZINC08409992	0
database	4566	ZINC00845252	0
database	6161	ZINC08411427	0
database	3792	ZINC02679404	0

database	2267	ZINC08438686	0
database	4159	ZINC00668317	0
database	3834	ZINC01801776	0
database	5961	ZINC08413403	0
database	3856	ZINC00715684	0
database	3826	ZINC00719525	0
database	3165	ZINC08435622	0
database	3685	ZINC08433275	0
database	3544	ZINC08435020	0
database	6795	ZINC00687330	0
database	5624	ZINC08417536	0
database	113	ZINC08442107	0
database	5018	ZINC08427099	0
database	3854	ZINC00711295	0
database	6560	ZINC12411205	0
database	2411	ZINC00624707	0
database	3287	ZINC19824421	0
database	6834	ZINC08400796	0
database	4565	ZINC00845251	0
database	5649	ZINC02876855	0
database	3589	ZINC08434881	0
database	386	ZINC08441900	0
database	1403	ZINC08439974	0
database	2749	ZINC12311320	0
database	6124	ZINC08411510	0
database	3445	ZINC08435339	0
database	120	ZINC08442097	0

database	3223	ZINC00411354	0
database	6495	ZINC19369491	0
database	4577	ZINC02740243	0
database	2753	ZINC00711910	0
database	4487	ZINC08431009	0
database	6403	ZINC02149646	0
database	6681	ZINC00687602	0
database	431	ZINC08441878	0
database	5633	ZINC01010564	0
database	5394	ZINC02059159	0
database	6971	ZINC02136040	0
database	1849	ZINC08438980	0
database	371	ZINC00882026	0
database	1536	ZINC08439705	0
database	2807	ZINC08437202	0
database	411	ZINC01126045	0
database	4242	ZINC00625534	0
database	2237	ZINC00626518	0
database	4866	ZINC00995805	0
database	4540	ZINC12462013	0
database	5911	ZINC08413551	0
database	4930	ZINC00711860	0
database	4618	ZINC08430397	0
database	4960	ZINC00711715	0
database	2167	ZINC02164600	0
database	548	ZINC00878064	0
database	5701	ZINC08415476	0

database	5348	ZINC08425660	0
database	3195	ZINC00976238	0
database	2907	ZINC02156382	0
database	2126	ZINC01413447	0
database	5309	ZINC02186165	0
database	2344	ZINC08438561	0
database	3723	ZINC01472175	0
database	5871	ZINC08413888	0
database	2245	ZINC06445949	0
database	3461	ZINC02070545	0
database	2030	ZINC01414769	0
database	1258	ZINC00210133	0
database	2550	ZINC03627534	0
database	4475	ZINC08431064	0
database	323	ZINC08441956	0
database	2923	ZINC04149943	0
database	4311	ZINC00997650	0
database	2203	ZINC08438758	0
database	5270	ZINC08426016	0
database	5534	ZINC00707346	0
database	1036	ZINC00702717	0
database	1346	ZINC06194703	0
database	2765	ZINC00693964	0
database	4383	ZINC00703529	0
database	5258	ZINC08426068	0
database	3532	ZINC08435036	0
database	1855	ZINC02168294	0

database	6598	ZINC19781721	0
database	5818	ZINC08413980	0
database	2568	ZINC02162053	0
database	293	ZINC00633686	0
database	7128	ZINC00676395	0
database	5822	ZINC08413973	0
database	4925	ZINC08427532	0
database	588	ZINC00877569	0
database	173	ZINC00664571	0
database	1793	ZINC08439173	0
database	6897	ZINC00686581	0
database	7071	ZINC08400485	0
database	6551	ZINC00705315	0
database	3991	ZINC00628439	0
database	7078	ZINC00999261	0
database	5595	ZINC00692962	0
database	590	ZINC08441622	0
database	2517	ZINC00623970	0
database	5734	ZINC08415058	0
database	6429	ZINC08406626	0
database	5095	ZINC08426775	0
database	5851	ZINC08413922	0
database	1840	ZINC08439051	0
database	4835	ZINC08429974	0
database	5651	ZINC08416939	0
database	785	ZINC00853634	0
database	7220	ZINC08400213	0

database	3763	ZINC01803303	0
database	2936	ZINC00645591	0
database	4382	ZINC00703528	0
database	6105	ZINC08411597	0
database	2132	ZINC01413487	0
database	1888	ZINC00627492	0
database	3346	ZINC01829128	0
database	7157	ZINC02135524	0
database	3070	ZINC02072199	0
database	1286	ZINC00702437	0
database	4047	ZINC08432486	0
database	282	ZINC09122186	0
database	6917	ZINC08400716	0
database	6591	ZINC04126513	0
database	3614	ZINC02067895	0
database	2377	ZINC08438527	0
database	1573	ZINC08439616	0
database	3475	ZINC08435255	0
database	1543	ZINC02170910	0
database	6832	ZINC08400798	0
database	6605	ZINC00704818	0
database	2144	ZINC08438767	0
database	2661	ZINC01113150	0
database	5097	ZINC08426774	0
database	3239	ZINC00976116	0
database	4202	ZINC02744560	0
database	3857	ZINC08432999	0

database	4084	ZINC08432345	0
database	6441	ZINC18043169	0
database	4650	ZINC00983247	0
database	306	ZINC08441973	0
database	5345	ZINC00297341	0
database	4370	ZINC00068345	0
database	2707	ZINC00694173	0
database	3657	ZINC09045647	0
database	5691	ZINC08415486	0
database	3491	ZINC00655246	0
database	2702	ZINC08437490	0
database	6197	ZINC08411315	0
database	5529	ZINC00821013	0
database	3671	ZINC08433301	0
database	6050	ZINC08413061	0
database	2994	ZINC00645562	0
database	591	ZINC08441621	0
database	2689	ZINC08437512	0
database	2756	ZINC00646580	0
database	5152	ZINC02060875	0
database	4791	ZINC00845109	0
database	2518	ZINC00623964	0
database	5241	ZINC00381691	0
database	3819	ZINC00719626	0
database	5132	ZINC03671791	0
database	5014	ZINC08427102	0
database	1806	ZINC08439144	0

database	4874	ZINC08427655	0
database	172	ZINC00664588	0
database	6590	ZINC04126512	0
database	4436	ZINC08431296	0
database	2192	ZINC01413490	0
database	6684	ZINC00687595	0
database	261	ZINC08442010	0
database	6104	ZINC08411597	0
database	3377	ZINC00827822	0
database	3380	ZINC00793758	0
database	3270	ZINC00625942	0
database	5972	ZINC08413383	0
database	3208	ZINC00868901	0
database	520	ZINC00881427	0
database	1525	ZINC00381912	0
database	1650	ZINC00643280	0
database	6073	ZINC08412464	0
database	3506	ZINC08435125	0
database	4028	ZINC00628155	0
database	1558	ZINC08439654	0
database	4108	ZINC00755316	0
database	5193	ZINC00641029	0
database	5949	ZINC08413411	0
database	6483	ZINC08405819	0
database	2755	ZINC00646580	0
database	1966	ZINC08438833	0
database	4917	ZINC00712027	0

database	4611	ZINC08430436	0
database	643	ZINC15885023	0
database	3694	ZINC17163311	0
database	4712	ZINC00665120	0
database	2814	ZINC08437179	0
database	6739	ZINC00687425	0
database	1135	ZINC08440596	0
database	3391	ZINC00975653	0
database	1906	ZINC08438903	0
database	4425	ZINC00730144	0
database	3822	ZINC00719594	0
database	6612	ZINC00704798	0
database	3021	ZINC08436909	0
database	1351	ZINC00298314	0
database	6275	ZINC04167867	0
database	1009	ZINC00702729	0
database	1901	ZINC01019550	0
database	6931	ZINC00686340	0
database	5745	ZINC08414751	0
database	6412	ZINC08407573	0
database	2879	ZINC00646261	0
database	247	ZINC08442026	0
database	558	ZINC00878053	0
database	776	ZINC01019934	0
database	6196	ZINC08411328	0
database	2140	ZINC08438768	0
database	4237	ZINC02084527	0

database	4213	ZINC00997876	0
database	3519	ZINC08435056	0
database	1929	ZINC00627339	0
database	4798	ZINC00664641	0
database	2700	ZINC01891206	0
database	5820	ZINC08413980	0
database	7148	ZINC00358881	0
database	5577	ZINC12413071	0
database	1267	ZINC08440218	0
database	1843	ZINC02168668	0
database	6609	ZINC08402924	0
database	5684	ZINC08415491	0
database	5622	ZINC08417546	0
database	3805	ZINC00723825	0
database	2652	ZINC08437661	0
database	3242	ZINC00976112	0
database	5106	ZINC08426766	0
database	1741	ZINC02502128	0
database	7103	ZINC08400463	0
database	1372	ZINC00659009	0
database	813	ZINC16956755	0
database	3916	ZINC00710381	0
database	3935	ZINC04178525	0
database	5850	ZINC08413922	0
database	5022	ZINC08427049	0
database	5208	ZINC08426278	0
database	1917	ZINC00627359	0

database	4391	ZINC08431438	0
database	3587	ZINC06444635	0
database	2182	ZINC02164573	0
database	5420	ZINC00729162	0
database	3292	ZINC13555376	0
database	5759	ZINC08414660	0
database	2667	ZINC00622664	0
database	4204	ZINC08431843	0
database	1680	ZINC00628601	0
database	7019	ZINC00676738	0
database	4276	ZINC00984942	0
database	2747	ZINC00646591	0
database	6192	ZINC08411337	0
database	3266	ZINC00625896	0
database	6646	ZINC00687713	0
database	4458	ZINC01026535	0
database	5465	ZINC00728674	0
database	4307	ZINC02742402	0
database	2801	ZINC08437218	0
database	4351	ZINC00991572	0
database	369	ZINC08441909	0
database	885	ZINC08441094	0
database	1831	ZINC02497232	0
database	3571	ZINC00675745	0
database	3376	ZINC15018525	0
database	6311	ZINC00985826	0
database	4513	ZINC08430894	0

database	3118	ZINC00862092	0
database	3160	ZINC00976249	0
database	5756	ZINC08414691	0
database	6826	ZINC00687239	0
database	3370	ZINC00872960	0
database	1495	ZINC00645888	0
database	6558	ZINC19791802	0
database	5441	ZINC00728819	0
database	4907	ZINC08427628	0
database	6493	ZINC19369491	0
database	625	ZINC00857211	0
database	2048	ZINC01414765	0
database	1892	ZINC00627471	0
database	5710	ZINC08415472	0
database	491	ZINC00650714	0
database	3973	ZINC08432715	0
database	2588	ZINC02161901	0
database	3899	ZINC02087938	0
database	3796	ZINC06144470	0
database	339	ZINC08441931	0
database	3175	ZINC00888202	0
database	477	ZINC08441855	0
database	7008	ZINC00676770	0
database	4504	ZINC02141464	0
database	3198	ZINC02072170	0
database	4783	ZINC06287761	0
database	4206	ZINC00997885	0

database	6592	ZINC01609672	0
database	809	ZINC13569448	0
database	2070	ZINC01414783	0
database	569	ZINC04089482	0
database	948	ZINC00648070	0
database	260	ZINC08442014	0
database	4912	ZINC08427618	0
database	2792	ZINC08437242	0
database	2402	ZINC00624773	0
database	1108	ZINC00702673	0
database	525	ZINC08441648	0
database	6168	ZINC08411415	0
database	3617	ZINC00808997	0
database	1099	ZINC02063121	0
database	1617	ZINC08439554	0
database	6343	ZINC00985562	0
database	3652	ZINC03000497	0
database	5176	ZINC08426397	0
database	818	ZINC16956754	0
database	6608	ZINC08402924	0
database	50	ZINC01019824	0
database	727	ZINC00703053	0
database	5403	ZINC01024812	0
database	512	ZINC00881505	0
database	1577	ZINC08439614	0
database	161	ZINC00664643	0
database	4604	ZINC00996410	0

database	1936	ZINC00627346	0
database	2269	ZINC08438685	0
database	205	ZINC08442066	0
database	5815	ZINC08413980	0
database	2011	ZINC09088730	0
database	4298	ZINC00845581	0
database	1879	ZINC00627687	0
database	2479	ZINC08438061	0
database	1309	ZINC00659329	0
database	4340	ZINC08431489	0
database	2356	ZINC00625733	0
database	4058	ZINC08432459	0
database	1637	ZINC04113088	0
database	3872	ZINC02259113	0
database	6902	ZINC01008557	0
database	5200	ZINC08426283	0
database	4900	ZINC08427629	0
database	5787	ZINC08414138	0
database	5164	ZINC00651270	0
database	3874	ZINC08432978	0
database	6983	ZINC00677007	0
database	7097	ZINC08400467	0
database	6569	ZINC00181991	0
database	6567	ZINC19791630	0
database	1504	ZINC00630220	0
database	6930	ZINC00686350	0
database	1086	ZINC00702689	0

database	4484	ZINC00984151	0
database	641	ZINC15885021	0
database	4126	ZINC00985531	0
database	3035	ZINC00645501	0
database	5160	ZINC02060869	0
database	691	ZINC00857460	0
database	3050	ZINC00693334	0
database	246	ZINC08442027	0
database	5019	ZINC03100121	0
database	2904	ZINC00693831	0
database	5025	ZINC03109483	0
database	2135	ZINC08438771	0
database	1949	ZINC08438865	0
database	6924	ZINC08400710	0
database	2225	ZINC00626552	0
database	6857	ZINC00686743	0
database	630	ZINC00971717	0
database	3871	ZINC02259113	0
database	1358	ZINC00659045	0
database	3355	ZINC00877358	0
database	4756	ZINC06287764	0
database	1418	ZINC09089086	0
database	6502	ZINC19369482	0
database	4715	ZINC02065569	0
database	4087	ZINC08432335	0
database	3480	ZINC00655497	0
database	3566	ZINC05687815	0

database	396	ZINC02064382	0
database	4512	ZINC00667913	0
database	2945	ZINC06144562	0
database	3501	ZINC08435145	0
database	4189	ZINC00997963	0
database	3731	ZINC00727868	0
database	1750	ZINC02503435	0
database	6069	ZINC00688314	0
database	2468	ZINC00660704	0
database	6890	ZINC00686592	0
database	6447	ZINC19119246	0
database	6973	ZINC01008267	0
database	5124	ZINC08426661	0
database	458	ZINC08441862	0
database	1481	ZINC08439790	0
database	5533	ZINC08424581	0
database	4033	ZINC01895890	0
database	4153	ZINC00707033	0
database	7142	ZINC02135700	0
database	1661	ZINC08439398	0
database	725	ZINC00862589	0
database	4344	ZINC00703578	0
database	3407	ZINC08435456	0
database	3643	ZINC08433436	0
database	2649	ZINC08437664	0
database	5689	ZINC08415486	0
database	4180	ZINC08431914	0

database	63	ZINC00633992	0
database	1991	ZINC02165100	0
database	3309	ZINC06445233	0
database	1554	ZINC08439655	0
database	1513	ZINC01013300	0
database	3901	ZINC08432890	0
database	1291	ZINC08440205	0
database	2261	ZINC01019496	0
database	746	ZINC08441410	0
database	2826	ZINC05360325	0
database	6087	ZINC00688116	0
database	685	ZINC08441472	0
database	381	ZINC08441902	0
database	5550	ZINC00727720	0
database	279	ZINC09122187	0
database	6556	ZINC19791805	0
database	4962	ZINC00711719	0
database	2583	ZINC08437720	0
database	5114	ZINC00354292	0
database	2825	ZINC05360331	0
database	7153	ZINC02135584	0
database	7105	ZINC08400461	0
database	6709	ZINC00687482	0
database	4318	ZINC00845124	0
database	70	ZINC00633955	0
database	3987	ZINC08432680	0
database	4768	ZINC00664805	0

database	6659	ZINC02146224	0
database	7167	ZINC00675561	0
database	3226	ZINC06442984	0
database	5687	ZINC08415486	0
database	5498	ZINC08424870	0
database	4334	ZINC00984834	0
database	1073	ZINC08440711	0
database	5681	ZINC08415491	0
database	457	ZINC08441862	0
database	2051	ZINC01414766	0
database	4381	ZINC08431460	0
database	253	ZINC08442023	0
database	4535	ZINC00483567	0
database	3761	ZINC08742399	0
database	116	ZINC08442106	0
database	3627	ZINC01952505	0
database	5086	ZINC00710529	0
database	6332	ZINC00985578	0
database	552	ZINC08441632	0
database	4973	ZINC00711483	0
database	2656	ZINC00622793	0
database	7176	ZINC00675389	0
database	6624	ZINC08402234	0
database	3598	ZINC08434874	0
database	4417	ZINC08431350	0
database	5680	ZINC08415491	0
database	5524	ZINC00628208	0

database	4030	ZINC02484243	0
database	6653	ZINC08400890	0
database	4492	ZINC08430987	0
database	439	ZINC00387261	0
database	4974	ZINC00711483	0
database	1015	ZINC00702731	0
database	1823	ZINC08439098	0
database	3168	ZINC08435621	0
database	3351	ZINC00975933	0
database	6577	ZINC08404176	0
database	0	ZINC20031600	0
database	7195	ZINC08400287	0
database	6639	ZINC08400936	0
database	1821	ZINC08439107	0
database	3097	ZINC00883984	0
database	2202	ZINC08438758	0
database	4098	ZINC08432297	0
database	883	ZINC00851206	0
database	3007	ZINC00693638	0
database	1807	ZINC08439142	0
database	3906	ZINC08432887	0
database	4465	ZINC00118692	0
database	4317	ZINC00997571	0
database	5614	ZINC08417570	0
database	3907	ZINC01895262	0
database	6957	ZINC01003746	0
database	5171	ZINC08426413	0

database	3414	ZINC08435428	0
database	993	ZINC08440837	0
database	1350	ZINC00702388	0
database	6444	ZINC18043169	0
database	3167	ZINC00778373	0
database	1385	ZINC13108926	0
database	2889	ZINC00646197	0
database	7040	ZINC08400521	0
database	1751	ZINC02170138	0
database	517	ZINC00881484	0
database	7200	ZINC08400279	0
database	5091	ZINC08426779	0
database	6323	ZINC00985623	0
database	1910	ZINC08438901	0
database	4816	ZINC00845115	0
database	4931	ZINC00711860	0
database	5458	ZINC08425062	0
database	3837	ZINC02680176	0
database	6867	ZINC00686697	0
database	5938	ZINC08413440	0
database	5076	ZINC08426799	0
database	3653	ZINC09045651	0
database	3130	ZINC00793623	0
database	352	ZINC00631600	0
database	383	ZINC02064396	0
database	37	ZINC00730699	0
database	228	ZINC00803800	0

database	930	ZINC08441014	0
database	6090	ZINC04457875	0
database	6370	ZINC02184954	0
database	330	ZINC08441943	0
database	3005	ZINC00645521	0
database	3269	ZINC00625942	0
database	3085	ZINC00884270	0
database	2624	ZINC00622907	0
database	7010	ZINC00676759	0
database	3157	ZINC00915443	0
database	6748	ZINC00687411	0
database	4767	ZINC00664813	0
database	2799	ZINC00646453	0
database	5967	ZINC08413387	0
database	5581	ZINC00863104	0
database	5805	ZINC08413985	0
database	1782	ZINC08439187	0
database	5021	ZINC00710996	0
database	2109	ZINC01011740	0
database	1285	ZINC00702437	0
database	3163	ZINC00778432	0
database	7186	ZINC00685546	0
database	1229	ZINC00659595	0
database	1421	ZINC00471779	0
database	4708	ZINC00983040	0
database	2960	ZINC00645576	0
database	6481	ZINC19220332	0

database	6310	ZINC00985826	0
database	3886	ZINC08432929	0
database	4424	ZINC00730146	0
database	3099	ZINC00883987	0
database	6425	ZINC02148749	0
database	4228	ZINC02742861	0
database	1443	ZINC09109830	0
database	5518	ZINC08424663	0
database	3176	ZINC00888157	0
database	1405	ZINC08439973	0
database	251	ZINC08442025	0
database	1803	ZINC02169330	0
database	5207	ZINC08426278	0
database	509	ZINC00881526	0
database	393	ZINC08441893	0
database	1282	ZINC08440213	0
database	3687	ZINC18179134	0
database	1414	ZINC00448009	0
database	5305	ZINC01811435	0
database	632	ZINC00241845	0
database	204	ZINC08442067	0
database	1851	ZINC08438976	0
database	1973	ZINC02165229	0
database	6956	ZINC01008284	0
database	3681	ZINC08433276	0
database	843	ZINC00851197	0
database	5268	ZINC08426022	0

database	1962	ZINC00627001	0
database	2938	ZINC00645591	0
database	1873	ZINC08438938	0
database	2995	ZINC00645562	0
database	2394	ZINC00624850	0
database	3728	ZINC09007850	0
database	6767	ZINC00687393	0
database	5982	ZINC08413327	0
database	4803	ZINC08430109	0
database	328	ZINC08441947	0
database	4423	ZINC08431335	0
database	3972	ZINC02747299	0
database	87	ZINC08442122	0
database	2719	ZINC08437441	0
database	2877	ZINC00646266	0
database	938	ZINC08440999	0
database	4389	ZINC00997469	0
database	2674	ZINC06283023	0
database	4128	ZINC01004580	0
database	2208	ZINC00626750	0
database	5080	ZINC08426798	0
database	402	ZINC02064379	0
database	3298	ZINC08817352	0
database	5814	ZINC08413980	0
database	4653	ZINC00802460	0
database	1196	ZINC00702561	0
database	6770	ZINC00687382	0

database	3133	ZINC00422350	0
database	6083	ZINC08411698	0
database	6899	ZINC00686578	0
database	6880	ZINC00686633	0
database	6353	ZINC08410137	0
database	5663	ZINC00691658	0
database	4223	ZINC00997827	0
database	170	ZINC05918661	0
database	4284	ZINC00984875	0
database	2797	ZINC00646452	0
database	4550	ZINC08430701	0
database	6359	ZINC08409992	0
database	6243	ZINC00987605	0
database	3511	ZINC00677526	0
database	1175	ZINC00844297	0
database	4175	ZINC00998010	0
database	4398	ZINC05359745	0
database	1215	ZINC08440410	0
database	6238	ZINC00987609	0
database	6781	ZINC00687358	0
database	6211	ZINC04473388	0
database	3209	ZINC06280814	0
database	6831	ZINC08400798	0
database	6984	ZINC08400617	0
database	2063	ZINC02164795	0
database	5197	ZINC08426285	0
database	6070	ZINC00688314	0

database	3952	ZINC06659995	0
database	1978	ZINC01011858	0
database	5761	ZINC08414604	0
database	3276	ZINC08435589	0
database	1682	ZINC00628605	0
database	3436	ZINC00663227	0
database	3319	ZINC06177078	0
database	6769	ZINC00687383	0
database	349	ZINC06195024	0
database	5785	ZINC08414158	0
database	3982	ZINC02354450	0
database	5773	ZINC08414444	0
database	1784	ZINC08439180	0
database	5307	ZINC08425899	0
database	424	ZINC00881847	0
database	6668	ZINC00687631	0
database	3631	ZINC08434761	0
database	1059	ZINC00702702	0
database	4092	ZINC08432318	0
database	4731	ZINC00664967	0
database	1702	ZINC08439334	0
database	3487	ZINC00975181	0
database	6402	ZINC08407886	0
database	4174	ZINC02084859	0
database	3124	ZINC00862127	0
database	1999	ZINC02165054	0
database	2415	ZINC08438289	0

database	5997	ZINC08413134	0
database	6911	ZINC00686471	0
database	5430	ZINC08425333	0
database	3624	ZINC02067623	0
database	3574	ZINC00430528	0
database	6128	ZINC08411500	0
database	186	ZINC04167844	0
database	6955	ZINC01008286	0
database	1413	ZINC00984064	0
database	6066	ZINC00688322	0
database	5979	ZINC08413327	0
database	2933	ZINC00645590	0
database	6106	ZINC08411537	0
database	13	ZINC19990070	0
database	6333	ZINC00985578	0
database	4247	ZINC01801817	0
database	3514	ZINC00975061	0
database	1872	ZINC08438939	0
database	6889	ZINC00686593	0
database	3678	ZINC00728051	0
database	2520	ZINC00623961	0
database	7042	ZINC08400518	0
database	1397	ZINC08439994	0
database	7227	ZINC00675033	0
database	3941	ZINC02679234	0
database	6146	ZINC08411449	0
database	5123	ZINC08426677	0

database	1775	ZINC15941858	0
database	5839	ZINC08413952	0
database	1492	ZINC00645909	0
database	5127	ZINC06245045	0
database	4825	ZINC08430006	0
database	5767	ZINC08414467	0
database	3114	ZINC06444464	0
database	4349	ZINC00703576	0
database	675	ZINC08441495	0
database	5531	ZINC08424584	0
database	5165	ZINC08426426	0
database	935	ZINC08441011	0
database	4955	ZINC00711739	0
database	4193	ZINC00997934	0
database	1552	ZINC08439659	0
database	6848	ZINC08400783	0
database	3488	ZINC08435190	0
database	2493	ZINC00624364	0
database	1057	ZINC00702701	0
database	3274	ZINC09280114	0
database	3569	ZINC00757974	0
database	5083	ZINC08426785	0
database	6522	ZINC19370923	0
database	5740	ZINC08414959	0
database	5730	ZINC08415078	0
database	3396	ZINC02071423	0
database	5318	ZINC02186108	0

database	4067	ZINC02085472	0
database	2570	ZINC02162038	0
database	274	ZINC08442004	0
database	6308	ZINC00985825	0
database	4272	ZINC00845123	0
database	1762	ZINC02170096	0
database	7048	ZINC08400514	0
database	5817	ZINC08413980	0
database	4437	ZINC02984680	0
database	6480	ZINC19791939	0
database	5313	ZINC08425801	0
database	2786	ZINC00646485	0
database	5676	ZINC08415497	0
database	6174	ZINC08411404	0
database	4161	ZINC00668301	0
database	4041	ZINC00669456	0
database	6883	ZINC00686607	0
database	7207	ZINC08400272	0
database	896	ZINC09357898	0
database	6626	ZINC08402160	0
database	6362	ZINC00983430	0
database	4331	ZINC08431507	0
database	2860	ZINC00646442	0
database	5237	ZINC02060714	0
database	553	ZINC00703107	0
database	192	ZINC00664411	0
database	6431	ZINC08406623	0

database	4493	ZINC02171275	0
database	1537	ZINC02170981	0
database	3347	ZINC02071981	0
database	2735	ZINC08437427	0
database	6705	ZINC00687505	0
database	4290	ZINC03210061	0
database	3725	ZINC08433182	0
database	6669	ZINC00687630	0
database	1971	ZINC02165240	0
database	7016	ZINC02135917	0
database	1254	ZINC08440243	0
database	3882	ZINC06135909	0
database	1277	ZINC00702446	0
database	5139	ZINC08426584	0
database	2451	ZINC00624534	0
database	4815	ZINC00982885	0
database	3142	ZINC00907636	0
database	1408	ZINC08439973	0
database	6804	ZINC02146044	0
database	3897	ZINC08432899	0
database	2206	ZINC08438758	0
database	388	ZINC08441899	0
database	45	ZINC08442272	0
database	6435	ZINC01022883	0
database	6251	ZINC00987227	0
database	5426	ZINC00708062	0
database	2084	ZINC01414786	0

database	1482	ZINC08439790	0
database	1683	ZINC00628608	0
database	4994	ZINC08427304	0
database	7225	ZINC08400199	0
database	3641	ZINC06904335	0
database	3543	ZINC08435021	0
database	6118	ZINC00688051	0
database	318	ZINC08441965	0
database	6387	ZINC08407962	0
database	1000	ZINC05921386	0
database	5643	ZINC08417213	0
database	6775	ZINC00687364	0
database	2416	ZINC08438289	0
database	3708	ZINC06444249	0
database	4710	ZINC08430231	0
database	5782	ZINC00688962	0
database	6166	ZINC08411415	0
database	4001	ZINC00670399	0
database	1811	ZINC01012792	0
database	787	ZINC08441355	0
database	4709	ZINC00665186	0
database	6109	ZINC00688052	0
database	3084	ZINC00884267	0
database	6254	ZINC00987221	0
database	2741	ZINC08437418	0
database	755	ZINC08441398	0
database	1606	ZINC00717217	0

database	2298	ZINC08438656	0
database	6649	ZINC00687677	0
database	4229	ZINC02742862	0
database	4194	ZINC00997932	0
database	2908	ZINC02156328	0
database	2642	ZINC08437672	0
database	3389	ZINC08435504	0
database	3147	ZINC08435629	0
database	4657	ZINC08430316	0
database	2184	ZINC08438763	0
database	4750	ZINC08430164	0
database	6406	ZINC08407872	0
database	3433	ZINC08435355	0
database	5445	ZINC00728813	0
database	825	ZINC08897840	0
database	5000	ZINC02205192	0
database	3581	ZINC00675540	0
database	2971	ZINC00645575	0
database	1722	ZINC02853302	0
database	2403	ZINC00624762	0
database	5214	ZINC08837018	0
database	6892	ZINC02143203	0
database	232	ZINC00803814	0
database	6228	ZINC08411113	0
database	4701	ZINC00845207	0
database	3427	ZINC00845545	0
database	2957	ZINC00645581	0

database	49	ZINC08442270	0
database	3481	ZINC00655467	0
database	3015	ZINC08436914	0
database	1225	ZINC05944355	0
database	2724	ZINC08437432	0
database	4939	ZINC01029954	0
database	4884	ZINC08427631	0
database	3426	ZINC00845544	0
database	2613	ZINC08437694	0
database	2115	ZINC01414763	0
database	2213	ZINC08438705	0
database	5954	ZINC08413407	0
database	7160	ZINC02135508	0
database	5370	ZINC06177320	0
database	16	ZINC19990034	0
database	6758	ZINC00687402	0
database	4697	ZINC08430248	0
database	995	ZINC08440835	0
database	1704	ZINC08439331	0
database	3305	ZINC08435579	0
database	5321	ZINC08425745	0
database	6903	ZINC00686513	0
database	6204	ZINC08411308	0
database	2611	ZINC00623005	0
database	1899	ZINC00627446	0
database	3072	ZINC00976394	0
database	2834	ZINC08685416	0

database	4898	ZINC08427629	0
database	7163	ZINC02135498	0
database	1799	ZINC15941298	0
database	74	ZINC00633953	0
database	4519	ZINC08430845	0
database	5928	ZINC08413491	0
database	110	ZINC08442108	0
database	4683	ZINC00665299	0
database	4218	ZINC00997865	0
database	2148	ZINC08438766	0
database	2859	ZINC00646442	0
database	2275	ZINC08438681	0
database	478	ZINC00703148	0
database	3937	ZINC08432835	0
database	1734	ZINC01013166	0
database	368	ZINC08441909	0
database	5627	ZINC00692407	0
database	4621	ZINC08430373	0
database	3089	ZINC00884089	0
database	2012	ZINC09331361	0
database	3437	ZINC08435353	0
database	5162	ZINC00969787	0
database	4026	ZINC00669611	0
database	6377	ZINC08408026	0
database	199	ZINC08442070	0
database	1396	ZINC08439994	0
database	1046	ZINC00702709	0

database	4181	ZINC01807350	0
database	4495	ZINC08430974	0
database	39	ZINC08442278	0
database	3849	ZINC06154170	0
database	1609	ZINC08439591	0
database	6302	ZINC00985862	0
database	623	ZINC00857217	0
database	3775	ZINC00723995	0
database	1572	ZINC08439616	0
database	6295	ZINC06406878	0
database	5918	ZINC08413517	0
database	2549	ZINC08437778	0
database	6650	ZINC02146240	0
database	5591	ZINC00693000	0
database	5527	ZINC00627179	0
database	5838	ZINC08413952	0
database	2774	ZINC01805330	0
database	4695	ZINC08430249	0
database	6060	ZINC08413048	0
database	5889	ZINC08413865	0
database	6194	ZINC08411329	0
database	5763	ZINC08414549	0
database	1748	ZINC08439260	0
database	5196	ZINC08426285	0
database	4606	ZINC08430469	0
database	2408	ZINC08438372	0
database	1339	ZINC08440140	0

database	7112	ZINC01008126	0
database	1983	ZINC02165187	0
database	3108	ZINC00915378	0
database	1499	ZINC00630239	0
database	4231	ZINC00984990	0
database	1072	ZINC08440711	0
database	4392	ZINC08431437	0
database	5824	ZINC08413973	0
database	3600	ZINC08434874	0
database	361	ZINC08441913	0
database	4483	ZINC08431030	0
database	375	ZINC08441904	0
database	4871	ZINC02158736	0
database	2281	ZINC08438677	0
database	5180	ZINC08426375	0
database	5050	ZINC08426828	0
database	3031	ZINC00693564	0
database	3151	ZINC00907469	0
database	4757	ZINC00729389	0
database	3422	ZINC08435402	0
database	5941	ZINC08413440	0
database	4301	ZINC00845572	0
database	5457	ZINC08425062	0
database	3583	ZINC01965163	0
database	3241	ZINC00976114	0
database	7082	ZINC00920498	0
database	3111	ZINC08435646	0

database	5724	ZINC08415433	0
database	4969	ZINC08427418	0
database	3811	ZINC08433104	0
database	3802	ZINC00723830	0
database	4706	ZINC02065623	0
database	897	ZINC09357898	0
database	3028	ZINC00645505	0
database	3180	ZINC08435617	0
database	980	ZINC00702760	0
database	6426	ZINC00067201	0
database	3441	ZINC02070798	0
database	6143	ZINC08411455	0
database	2333	ZINC08438563	0
database	6241	ZINC00987604	0
database	4230	ZINC00984989	0
database	3071	ZINC02072198	0
database	1478	ZINC08439792	0
database	7104	ZINC08400463	0
database	4169	ZINC02084908	0
database	3823	ZINC00719590	0
database	2618	ZINC00622925	0
database	4056	ZINC00669387	0
database	215	ZINC08442061	0
database	301	ZINC08441976	0
database	93	ZINC08442118	0
database	1222	ZINC00702509	0
database	5125	ZINC08426660	0

database	6898	ZINC00686579	0
database	4539	ZINC08430749	0
database	965	ZINC06445809	0
database	5406	ZINC00292288	0
database	3013	ZINC00645516	0
database	5133	ZINC08426645	0
database	5126	ZINC08426660	0
database	676	ZINC08441495	0
database	4096	ZINC00707079	0
database	97	ZINC08442117	0
database	1595	ZINC00717214	0
database	1939	ZINC08438884	0
database	3381	ZINC08435518	0
database	2406	ZINC00624750	0
database	6088	ZINC08411690	0
database	5888	ZINC08413865	0
database	6566	ZINC19791625	0
database	4120	ZINC08432213	0
database	560	ZINC00878051	0
database	1395	ZINC08439994	0
database	1835	ZINC02497236	0
database	2251	ZINC06445943	0
database	3020	ZINC00645515	0
database	501	ZINC00881543	0
database	75	ZINC08442171	0
database	452	ZINC08441863	0
database	6985	ZINC08400617	0

database	4698	ZINC08430248	0
database	3630	ZINC00974456	0
database	6699	ZINC00687546	0
database	4690	ZINC02065664	0
database	6777	ZINC00687362	0
database	4777	ZINC08430132	0
database	5315	ZINC03669009	0
database	1365	ZINC08440096	0
database	803	ZINC00853497	0
database	1705	ZINC08439330	0
database	7134	ZINC08400423	0
database	4500	ZINC00983963	0
database	1026	ZINC00702724	0
database	4961	ZINC00711719	0
database	6188	ZINC08411371	0
database	7210	ZINC08400241	0
database	3992	ZINC00986177	0
database	1743	ZINC01013162	0
database	1078	ZINC08440709	0
database	2691	ZINC06147720	0
database	7170	ZINC00675541	0
database	4846	ZINC02093670	0
database	6614	ZINC00704796	0
database	3493	ZINC00655195	0
database	4780	ZINC00664766	0
database	3246	ZINC00868854	0
database	4877	ZINC00712059	0

database	1967	ZINC02165261	0
database	3474	ZINC06444891	0
database	2794	ZINC08437236	0
database	2680	ZINC00646870	0
database	2558	ZINC01011366	0
database	7152	ZINC01008093	0
database	1961	ZINC08438850	0
database	5874	ZINC08413885	0
database	5234	ZINC08426124	0
database	146	ZINC00664719	0
database	3334	ZINC08435575	0
database	1805	ZINC08439146	0
database	6015	ZINC08413110	0
database	7009	ZINC00676763	0
database	3312	ZINC06445233	0
database	3123	ZINC00862128	0
database	4739	ZINC00729394	0
database	2525	ZINC00623804	0
database	7143	ZINC00675690	0
database	1486	ZINC08439788	0
database	3827	ZINC00719444	0
database	1593	ZINC00717214	0
database	6794	ZINC00687334	0
database	1862	ZINC00683536	0
database	5051	ZINC08426826	0
database	3061	ZINC01018826	0
database	837	ZINC08441257	0

database	1289	ZINC08440205	0
database	6838	ZINC08400793	0
database	3144	ZINC00907609	0
database	6266	ZINC04168774	0
database	3182	ZINC08435616	0
database	1667	ZINC08439385	0
database	2522	ZINC00623824	0
database	6409	ZINC01023116	0
database	4987	ZINC00711281	0
database	836	ZINC08441257	0
database	1429	ZINC00843797	0
database	1193	ZINC06445251	0
database	5809	ZINC08413980	0
database	3876	ZINC08432974	0
database	346	ZINC08441924	0
database	1749	ZINC02503435	0
database	4177	ZINC08431933	0
database	2319	ZINC00625874	0
database	1463	ZINC00717758	0
database	1874	ZINC08438937	0
database	6717	ZINC00687461	0
database	4903	ZINC08427628	0
database	2324	ZINC00625888	0
database	3885	ZINC02088020	0
database	5926	ZINC08413491	0
database	3224	ZINC00863677	0
database	1362	ZINC00702370	0

database	890	ZINC06556763	0
database	6321	ZINC00985622	0
database	1098	ZINC08440687	0
database	6282	ZINC06300265	0
database	3759	ZINC08742399	0
database	6634	ZINC00687879	0
database	587	ZINC00877569	0
database	2234	ZINC00626508	0
database	1896	ZINC00627460	0
database	3404	ZINC08435470	0
database	3807	ZINC00723827	0
database	3736	ZINC00724094	0
database	2676	ZINC06283020	0
database	4160	ZINC00628506	0
database	782	ZINC08441362	0
database	4584	ZINC00667216	0
database	3267	ZINC00625908	0
database	3210	ZINC00867614	0
database	705	ZINC08441459	0
database	1393	ZINC15906697	0
database	6047	ZINC08413066	0
database	3478	ZINC00655500	0
database	6597	ZINC19781721	0
database	4839	ZINC08429973	0
database	1359	ZINC00702370	0
database	6286	ZINC08410677	0
database	4348	ZINC00703576	0

database	2965	ZINC00645577	0
database	2616	ZINC02161721	0
database	4693	ZINC08430249	0
database	4166	ZINC08432021	0
database	502	ZINC00881541	0
database	2111	ZINC01414732	0
database	3103	ZINC00883782	0
database	4152	ZINC08432143	0
database	2779	ZINC00646486	0
database	6078	ZINC08411843	0
database	7064	ZINC02158045	0
database	5754	ZINC08414739	0
database	1711	ZINC08439318	0
database	2557	ZINC01011367	0
database	583	ZINC00877571	0
database	5342	ZINC00629649	0
database	524	ZINC07077628	0
database	1487	ZINC00645912	0
database	1830	ZINC02497232	0
database	3440	ZINC00663180	0
database	5035	ZINC08426912	0
database	829	ZINC08897821	0
database	6870	ZINC00686689	0
database	6411	ZINC08407629	0
database	1489	ZINC00645910	0
database	4536	ZINC08430766	0
database	4373	ZINC00703540	0

database	4020	ZINC02085637	0
database	1633	ZINC04113089	0
database	4419	ZINC08431344	0
database	3088	ZINC00884087	0
database	3321	ZINC06177078	0
database	7123	ZINC01008116	0
database	2027	ZINC08438776	0
database	6970	ZINC02136050	0
database	4651	ZINC00996301	0
database	1208	ZINC00837684	0
database	6850	ZINC08400781	0
database	5861	ZINC08413890	0
database	611	ZINC08441602	0
database	3517	ZINC08435058	0
database	724	ZINC15952849	0
database	185	ZINC04167844	0
database	937	ZINC08441000	0
database	4833	ZINC00982728	0
database	101	ZINC08442114	0
database	6221	ZINC08411259	0
database	5037	ZINC01949321	0
database	314	ZINC06446604	0
database	6125	ZINC08411504	0
database	3095	ZINC00676209	0
database	5355	ZINC08425651	0
database	4935	ZINC08427499	0
database	4774	ZINC00996060	0

database	7083	ZINC02252325	0
database	3596	ZINC08434874	0
database	6648	ZINC00687678	0
database	6594	ZINC08403501	0
database	6712	ZINC00687471	0
database	6101	ZINC08411605	0
database	3055	ZINC08436817	0
database	3222	ZINC00862948	0
database	649	ZINC00971704	0
database	5333	ZINC08425680	0
database	4460	ZINC00997247	0
database	6234	ZINC13136834	0
database	3001	ZINC00645535	0
database	6126	ZINC08411504	0
database	278	ZINC01107287	0
database	6932	ZINC02142426	0
database	4737	ZINC08430171	0
database	61	ZINC00633997	0
database	345	ZINC08441926	0
database	3604	ZINC00819722	0
database	6914	ZINC02142784	0
database	4227	ZINC00706803	0
database	6849	ZINC08400782	0
database	4320	ZINC08431534	0
database	761	ZINC08441394	0
database	3526	ZINC00127464	0
database	7044	ZINC08400517	0

database	3011	ZINC08436919	0
database	6565	ZINC19791633	0
database	6084	ZINC08411698	0
database	4929	ZINC00711860	0
database	4682	ZINC00665323	0
database	3714	ZINC00727935	0
database	1060	ZINC00702702	0
database	3781	ZINC08433138	0
database	4052	ZINC08432474	0
database	4440	ZINC08431261	0
database	608	ZINC15880059	0
database	4742	ZINC08430167	0
database	2984	ZINC13122227	0
database	432	ZINC08441878	0
database	2163	ZINC02164609	0
database	6352	ZINC00846344	0
database	3164	ZINC00778431	0
database	6499	ZINC08405426	0
database	2266	ZINC08438686	0
database	653	ZINC13123303	0
database	650	ZINC13123301	0
database	435	ZINC08441877	0
database	4339	ZINC08431489	0
database	2556	ZINC02162273	0
database	6508	ZINC00705668	0
database	284	ZINC08836444	0
database	6803	ZINC02146053	0

database	6524	ZINC19370923	0
database	5096	ZINC08426775	0
database	7	ZINC03901268	0
database	5136	ZINC08856047	0
database	1548	ZINC08439662	0
database	1017	ZINC00702732	0
database	6257	ZINC00987216	0
database	5368	ZINC00647281	0
database	3580	ZINC00675538	0
database	2771	ZINC02158876	0
database	6317	ZINC00985634	0
database	1904	ZINC01019551	0
database	1171	ZINC00844383	0
database	6710	ZINC00687476	0
database	6396	ZINC08407905	0
database	28	ZINC18141403	0
database	1426	ZINC00702308	0
database	6993	ZINC08400612	0
database	453	ZINC08441863	0
database	7199	ZINC08400281	0
database	855	ZINC08441221	0
database	2346	ZINC00625759	0
database	864	ZINC00852498	0
database	7219	ZINC08400213	0
database	6139	ZINC08411466	0
database	473	ZINC00881550	0
database	3359	ZINC08435555	0

database	1773	ZINC02993022	0
database	1674	ZINC08439381	0
database	7070	ZINC08400485	0
database	5312	ZINC08425804	0
database	4867	ZINC09110317	0
database	176	ZINC00664574	0
database	6561	ZINC00347319	0
database	4183	ZINC00174709	0
database	2721	ZINC08437433	0
database	2381	ZINC08438501	0
database	3420	ZINC02071188	0
database	6445	ZINC19119246	0
database	1438	ZINC00721236	0
database	1416	ZINC09089086	0
database	1852	ZINC08438974	0
database	226	ZINC00803806	0
database	165	ZINC00664623	0
database	6953	ZINC01008292	0
database	6621	ZINC08402538	0
database	4869	ZINC02068445	0
database	848	ZINC05313299	0
database	5472	ZINC08424954	0
database	7110	ZINC08400454	0
database	6763	ZINC00687397	0
database	6201	ZINC08411314	0
database	1551	ZINC08439659	0
database	3733	ZINC00727870	0

database	2129	ZINC01413486	0
database	1152	ZINC08440584	0
database	6320	ZINC00985622	0
database	292	ZINC00660158	0
database	4554	ZINC00996574	0
database	2805	ZINC08437204	0
database	4418	ZINC00703402	0
database	3153	ZINC00907402	0
database	1723	ZINC06270551	0
database	6620	ZINC00704786	0
database	3735	ZINC00724091	0
database	3724	ZINC01472176	0
database	6162	ZINC08411424	0
database	454	ZINC08441863	0
database	3065	ZINC06444433	0
database	6373	ZINC08408111	0
database	3233	ZINC08435596	0
database	3842	ZINC00628590	0
database	702	ZINC08441462	0
database	5415	ZINC00799728	0
database	3247	ZINC00864421	0
database	5340	ZINC00647507	0
database	3313	ZINC06136516	0
database	123	ZINC00664784	0
database	6990	ZINC01008244	0
database	5806	ZINC08413985	0
database	3576	ZINC00183602	0

database	3696	ZINC17163315	0
database	5377	ZINC02738827	0
database	932	ZINC00852798	0
database	2987	ZINC13122228	0
database	922	ZINC08441026	0
database	886	ZINC05520252	0
database	5390	ZINC01229187	0
database	1583	ZINC08439613	0
database	607	ZINC15880059	0
database	4297	ZINC00845580	0
database	4219	ZINC08431831	0
database	2094	ZINC02164716	0
database	1270	ZINC08440218	0
database	4616	ZINC00996343	0
database	516	ZINC00881486	0
database	5110	ZINC08426765	0
database	6272	ZINC04167868	0
database	3251	ZINC00864422	0
database	2414	ZINC08438289	0
database	3502	ZINC06474964	0
database	659	ZINC13123055	0
database	4376	ZINC00703541	0
database	2979	ZINC00645568	0
database	6395	ZINC08407911	0
database	3212	ZINC00867611	0
database	78	ZINC08442165	0
database	500	ZINC00881545	0

database	6374	ZINC08408093	0
database	2413	ZINC00624691	0
database	4027	ZINC00669613	0
database	5608	ZINC00692632	0
database	4414	ZINC08431356	0
database	4410	ZINC08431359	0
database	290	ZINC00633738	0
database	2186	ZINC01413498	0
database	6570	ZINC00181991	0
database	3603	ZINC00819726	0
database	752	ZINC08441400	0
database	2459	ZINC00660936	0
database	5816	ZINC08413980	0
database	1112	ZINC08440681	0
database	7109	ZINC08400455	0
database	3712	ZINC08433220	0
database	1905	ZINC08438903	0
database	4965	ZINC08872338	0
database	924	ZINC08441021	0
database	3136	ZINC00976267	0
database	3677	ZINC09301975	0
database	4432	ZINC08431304	0
database	2477	ZINC08438065	0
database	7063	ZINC02158045	0
database	5395	ZINC08425462	0
database	3356	ZINC08435556	0
database	6829	ZINC08400803	0

database	20	ZINC19794473	0
database	4482	ZINC08431033	0
database	4337	ZINC00703585	0
database	716	ZINC15884980	0
database	36	ZINC08442293	0
database	4741	ZINC00729395	0
database	6940	ZINC01008320	0
database	6915	ZINC02142780	0
database	3628	ZINC08434778	0
database	2934	ZINC00645590	0
database	957	ZINC08440929	0
database	4283	ZINC00997699	0
database	687	ZINC08441471	0
database	2445	ZINC08438187	0
database	100	ZINC08442115	0
database	4197	ZINC08431861	0
database	3092	ZINC00676203	0
database	2316	ZINC00625874	0
database	175	ZINC00633881	0
database	1877	ZINC00627695	0
database	6097	ZINC13147206	0
database	4844	ZINC02093668	0
database	3783	ZINC08433137	0
database	5444	ZINC00728812	0
database	4999	ZINC08427239	0
database	256	ZINC08442020	0
database	5963	ZINC08413403	0

database	6928	ZINC08400705	0
database	2127	ZINC01011730	0
database	1876	ZINC02167795	0
database	3773	ZINC01803290	0
database	206	ZINC08442066	0
database	6131	ZINC08411476	0
database	2470	ZINC08438079	0
database	4316	ZINC00997578	0
database	4184	ZINC02083251	0
database	3096	ZINC00883982	0
database	6995	ZINC00676887	0
database	5225	ZINC00678048	0
database	5976	ZINC08413383	0
database	5212	ZINC08837018	0
database	5119	ZINC08426689	0
database	5450	ZINC08425130	0
database	5462	ZINC00728712	0
database	236	ZINC08442036	0
database	3432	ZINC08435356	0
database	6946	ZINC08400677	0
database	2436	ZINC00624587	0
database	5566	ZINC08424401	0
database	2684	ZINC08437536	0
database	766	ZINC00703044	0
database	1880	ZINC00627551	0
database	6360	ZINC12563589	0
database	1021	ZINC00702727	0

database	1279	ZINC08440216	0
database	578	ZINC08441626	0
database	6329	ZINC00985592	0
database	6099	ZINC08411616	0
database	3593	ZINC00194823	0
database	5844	ZINC08413942	0
database	1456	ZINC00720885	0
database	4267	ZINC00984956	0
database	784	ZINC09070026	0
database	1921	ZINC00627363	0
database	3371	ZINC00872941	0
database	7012	ZINC00676756	0
database	1123	ZINC08440677	0
database	5235	ZINC02060715	0
database	2575	ZINC02162000	0
database	4892	ZINC08427630	0
database	1422	ZINC00702307	0
database	7018	ZINC00676740	0
database	949	ZINC00648070	0
database	5858	ZINC08413894	0
database	7045	ZINC01008161	0
database	2005	ZINC01011792	0
database	4443	ZINC08431244	0
database	6230	ZINC00988057	0
database	4366	ZINC00703551	0
database	7091	ZINC02252328	0
database	6226	ZINC08411187	0

database	545	ZINC00878068	0
database	7043	ZINC08400517	0
database	3200	ZINC08435612	0
database	5609	ZINC00692632	0
database	748	ZINC08441403	0
database	2103	ZINC01413449	0
database	5937	ZINC08413440	0
database	7094	ZINC08400468	0
database	379	ZINC08441902	0
database	3534	ZINC08435034	0
database	5413	ZINC00729177	0
database	421	ZINC00631391	0
database	7124	ZINC00358914	0
database	114	ZINC08442106	0
database	2139	ZINC08438769	0
database	5703	ZINC08415476	0
database	38	ZINC00730699	0
database	1360	ZINC00702370	0
database	4469	ZINC08431156	0
database	6586	ZINC00704874	0
database	6430	ZINC08406626	0
database	4788	ZINC00845108	0
database	2988	ZINC00645564	0
database	2439	ZINC00624576	0
database	2993	ZINC00645565	0
database	4744	ZINC08430167	0
database	389	ZINC08441899	0

database	6147	ZINC08411449	0
database	2363	ZINC09046651	0
database	5391	ZINC01229188	0
database	4567	ZINC00667511	0
database	365	ZINC08441911	0
database	6989	ZINC00676998	0
database	1050	ZINC00702699	0
database	3439	ZINC08435352	0
database	2102	ZINC02164705	0
database	4986	ZINC08427310	0
database	6344	ZINC00985555	0
database	4501	ZINC00983948	0
database	1151	ZINC08440584	0
database	5381	ZINC00293338	0
database	5573	ZINC00799069	0
database	5242	ZINC00381691	0
database	1294	ZINC00702433	0
database	5516	ZINC02058121	0
database	303	ZINC08441974	0
database	5916	ZINC08413520	0
database	5016	ZINC08427102	0
database	1566	ZINC08439637	0
database	4561	ZINC02140778	0
database	6922	ZINC08400712	0
database	3520	ZINC08435055	0
database	4441	ZINC08431250	0
database	1332	ZINC08440170	0

database	4246	ZINC00997761	0
database	1261	ZINC00198961	0
database	1174	ZINC00844296	0
database	3450	ZINC08435308	0
database	4039	ZINC08432503	0
database	1631	ZINC04113089	0
database	4934	ZINC08427506	0
database	4957	ZINC00711715	0
database	7039	ZINC00676602	0
database	5667	ZINC00691563	0
database	3594	ZINC08434874	0
database	4652	ZINC00802459	0
database	5800	ZINC08413985	0
database	2837	ZINC08437153	0
database	7151	ZINC00675654	0
database	6992	ZINC08400612	0
database	542	ZINC00703105	0
database	2693	ZINC06147718	0
database	377	ZINC00881990	0
database	1541	ZINC08439703	0
database	6596	ZINC08403429	0
database	4253	ZINC00984972	0
database	1658	ZINC06321516	0
database	2628	ZINC01011075	0
database	6529	ZINC19370919	0
database	2010	ZINC09087398	0
database	1791	ZINC01013027	0

database	6526	ZINC19370925	0
database	6300	ZINC00985861	0
database	5092	ZINC08426779	0
database	5183	ZINC08426341	0
database	2906	ZINC02156399	0
database	4045	ZINC08432499	0
database	2195	ZINC08438760	0
database	3637	ZINC00889265	0
database	3331	ZINC06156995	0
database	62	ZINC08442204	0
database	105	ZINC01102227	0
database	310	ZINC06446605	0
database	5324	ZINC00831561	0
database	3012	ZINC00645516	0
database	3343	ZINC08435564	0
database	5120	ZINC08426679	0
database	6806	ZINC02146032	0
database	2141	ZINC01413488	0
database	4037	ZINC02279945	0
database	2882	ZINC00646245	0
database	2865	ZINC00646443	0
database	2982	ZINC00645569	0
database	6760	ZINC00687400	0
database	5711	ZINC08415472	0
database	3044	ZINC00645295	0
database	2696	ZINC01893075	0
database	4329	ZINC02084295	0

database	4017	ZINC00986101	0
database	4256	ZINC00997738	0
database	6433	ZINC00118200	0
database	5868	ZINC08413888	0
database	3216	ZINC00877794	0
database	6965	ZINC08400654	0
database	6865	ZINC00686705	0
database	2811	ZINC08437198	0
database	6218	ZINC08411267	0
database	4754	ZINC08430159	0
database	250	ZINC08442025	0
database	3039	ZINC08436885	0
database	6550	ZINC00705314	0
database	5749	ZINC08414748	0
database	6580	ZINC00127000	0
database	1034	ZINC00702716	0
database	2373	ZINC08438529	0
database	6304	ZINC00985843	0
database	4259	ZINC00845216	0
database	6064	ZINC00688321	0
database	767	ZINC00703044	0
database	6464	ZINC19312813	0
database	6511	ZINC19369473	0
database	1364	ZINC08440096	0
database	1221	ZINC00702509	0
database	3379	ZINC08435522	0
database	6334	ZINC00985579	0

database	6552	ZINC02181619	0
database	6189	ZINC08411364	0
database	4646	ZINC00983250	0
database	1692	ZINC08439348	0
database	4634	ZINC08430356	0
database	1656	ZINC00643217	0
database	3774	ZINC01803291	0
database	7015	ZINC02135917	0
database	2478	ZINC08438063	0
database	2663	ZINC00622712	0
database	3091	ZINC00884093	0
database	6977	ZINC00677050	0
database	5764	ZINC08414543	0
database	4808	ZINC00996029	0
database	2361	ZINC08438558	0
database	5387	ZINC08425481	0
database	2546	ZINC00623492	0
database	5416	ZINC00799729	0
database	1028	ZINC00702725	0
database	6041	ZINC08413079	0
database	4773	ZINC02061579	0
database	6129	ZINC08411500	0
database	6935	ZINC02142344	0
database	6801	ZINC02146116	0
database	5020	ZINC03067959	0
database	5059	ZINC00710619	0
database	1400	ZINC00651665	0

database	5922	ZINC08413517	0
database	1881	ZINC00627553	0
database	3177	ZINC00869903	0
database	5071	ZINC08426810	0
database	6199	ZINC08411315	0
database	2708	ZINC08437470	0
database	6664	ZINC00687661	0
database	6423	ZINC08407207	0
database	4730	ZINC00996140	0
database	5827	ZINC08413954	0
database	925	ZINC08441019	0
database	6523	ZINC19370923	0
database	433	ZINC08441878	0
database	6800	ZINC00687324	0
database	5042	ZINC08426875	0
database	5417	ZINC08425384	0
database	4734	ZINC00996134	0
database	4560	ZINC08430662	0
database	47	ZINC08442271	0
database	621	ZINC00862927	0
database	2943	ZINC00645585	0
database	5563	ZINC00799256	0
database	5637	ZINC00692264	0
database	4145	ZINC00985223	0
database	68	ZINC08442186	0
database	5053	ZINC00710679	0
database	6793	ZINC00687335	0

database	6786	ZINC00687346	0
database	428	ZINC08441879	0
database	1614	ZINC00717197	0
database	1841	ZINC08439045	0
database	6751	ZINC00687408	0
database	6264	ZINC04168776	0
database	4196	ZINC08431861	0
database	4872	ZINC08427657	0
database	3187	ZINC00897341	0
database	1473	ZINC08439817	0
database	5953	ZINC08413407	0
database	3615	ZINC08434825	0
database	729	ZINC00862489	0
database	5517	ZINC08424686	0
database	5438	ZINC00728826	0
database	4660	ZINC08430310	0
database	2880	ZINC00646262	0
database	434	ZINC08441877	0
database	1211	ZINC00666139	0
database	6974	ZINC01008265	0
database	1995	ZINC02165076	0
database	5227	ZINC08426172	0
database	1931	ZINC00627341	0
database	4143	ZINC00707046	0
database	4851	ZINC02093602	0
database	26	ZINC06182368	0
database	6008	ZINC08413115	0

database	4457	ZINC01026534	0
database	624	ZINC00857215	0
database	4528	ZINC00845104	0
database	1417	ZINC09089086	0
database	2902	ZINC09186823	0
database	566	ZINC08441630	0
database	912	ZINC08441036	0
database	7144	ZINC00675692	0
database	4678	ZINC00845121	0
database	3893	ZINC08432904	0
database	3561	ZINC08434955	0
database	6572	ZINC01953653	0
database	3400	ZINC08435485	0
database	6127	ZINC08411504	0
database	5626	ZINC00692407	0
database	2626	ZINC08437688	0
database	5542	ZINC00228214	0
database	3817	ZINC08433049	0
database	3019	ZINC00645515	0
database	690	ZINC00857459	0
database	1758	ZINC00715098	0
database	3254	ZINC06406259	0
database	3960	ZINC00670792	0
database	4468	ZINC00984332	0
database	4265	ZINC08431730	0
database	7030	ZINC08400536	0
database	2074	ZINC01414784	0

database	2272	ZINC08438683	0
database	3621	ZINC02067662	0
database	543	ZINC00703105	0
database	6036	ZINC08413085	0
database	6296	ZINC00985879	0
database	1121	ZINC08440677	0
database	2846	ZINC08437142	0
database	6878	ZINC00686660	0
database	271	ZINC08442006	0
database	1304	ZINC08440200	0
database	4707	ZINC01868164	0
database	3423	ZINC08435397	0
database	5596	ZINC00692958	0
database	5666	ZINC00691563	0
database	4988	ZINC00711282	0
database	1392	ZINC15906697	0
database	6401	ZINC08407888	0
database	3688	ZINC08433259	0
database	5760	ZINC08414628	0
database	4648	ZINC00983251	0
database	3586	ZINC08434893	0
database	6961	ZINC02136086	0
database	6399	ZINC08407892	0
database	2515	ZINC00623995	0
database	5504	ZINC08424840	0
database	4330	ZINC00294945	0
database	701	ZINC08441462	0

database	1727	ZINC08439297	0
database	3662	ZINC08433344	0
database	4212	ZINC00985026	0
database	7111	ZINC02197315	0
database	6484	ZINC08405819	0
database	4668	ZINC00665450	0
database	6291	ZINC02492153	0
database	2521	ZINC08437866	0
database	1115	ZINC00702674	0
database	2732	ZINC08437428	0
database	2455	ZINC00624458	0
database	3459	ZINC08435285	0
database	3684	ZINC08433275	0
database	7056	ZINC08400507	0
database	6871	ZINC08400770	0
database	263	ZINC08442010	0
database	2622	ZINC00622905	0
database	3361	ZINC00047416	0
database	5040	ZINC03057611	0
database	4625	ZINC08430365	0
database	3750	ZINC09301884	0
database	6186	ZINC08411380	0
database	6029	ZINC08413100	0
database	4526	ZINC02740371	0
database	1908	ZINC01019553	0
database	547	ZINC00878066	0
database	1516	ZINC00630103	0

database	2678	ZINC02159741	0
database	3557	ZINC08434969	0
database	6996	ZINC08400611	0
database	3112	ZINC00890119	0
database	4658	ZINC00665794	0
database	582	ZINC00877571	0
database	2701	ZINC01891233	0
database	3722	ZINC02106975	0
database	4702	ZINC00983052	0
database	6528	ZINC19370919	0
database	4861	ZINC02082144	0
database	4343	ZINC00703578	0
database	59	ZINC08442210	0
database	4579	ZINC08430622	0
database	3768	ZINC01803348	0
database	3074	ZINC08435661	0
database	4059	ZINC00985961	0
database	4371	ZINC08431472	0
database	7058	ZINC00999300	0
database	5877	ZINC08413885	0
database	203	ZINC08442067	0
database	4795	ZINC02144395	0
database	1139	ZINC08440596	0
database	252	ZINC08442023	0
database	3993	ZINC00986178	0
database	7158	ZINC02135518	0
database	6462	ZINC19312800	0

database	6438	ZINC06713282	0
database	2321	ZINC00625888	0
database	4111	ZINC00668742	0
database	5481	ZINC08424944	0
database	584	ZINC08441623	0
database	4647	ZINC00983251	0
database	3656	ZINC09045647	0
database	1218	ZINC08440407	0
database	1922	ZINC00627363	0
database	1485	ZINC08439788	0
database	7028	ZINC00359046	0
database	6578	ZINC00704913	0
database	797	ZINC00702989	0
database	6414	ZINC08407464	0
database	4938	ZINC02186542	0
database	2157	ZINC01413516	0
database	1860	ZINC02484245	0
database	6816	ZINC00687255	0
database	1493	ZINC00645898	0
database	1386	ZINC13108926	0
database	4292	ZINC00845703	0
database	1857	ZINC08438967	0
database	2974	ZINC00645570	0
database	5650	ZINC02876856	0
database	2149	ZINC01413496	0
database	6969	ZINC02136056	0
database	1505	ZINC02502423	0

database	6563	ZINC19791666	0
database	2250	ZINC06445928	0
database	6533	ZINC19370921	0
database	32	ZINC18141403	0
database	317	ZINC08441966	0
database	3975	ZINC01896240	0
database	1142	ZINC08440596	0
database	7093	ZINC08400468	0
database	6733	ZINC00687431	0
database	5876	ZINC08413885	0
database	240	ZINC08442030	0
database	3881	ZINC06135995	0
database	5188	ZINC08426304	0
database	6065	ZINC00688322	0
database	4995	ZINC08427303	0
database	4770	ZINC08430146	0
database	6267	ZINC04168774	0
database	4557	ZINC08430665	0
database	5245	ZINC00708700	0
database	12	ZINC19990070	0
database	1515	ZINC13545840	0
database	2788	ZINC00646482	0
database	1875	ZINC02167834	0
database	5620	ZINC00692502	0
database	7020	ZINC00676724	0
database	3559	ZINC00187217	0
database	3706	ZINC02107103	0

database	3551	ZINC00201649	0
database	7137	ZINC08400418	0
database	163	ZINC00664621	0
database	3162	ZINC00778436	0
database	2047	ZINC01414765	0
database	2665	ZINC00622718	0
database	4255	ZINC02084506	0
database	3638	ZINC06904335	0
database	5568	ZINC12462349	0
database	5662	ZINC00691658	0
database	863	ZINC08441176	0
database	3017	ZINC08436913	0
database	2867	ZINC00646443	0
database	3659	ZINC02303173	0
database	216	ZINC00633832	0
database	1043	ZINC00702714	0
database	239	ZINC08442030	0
database	2241	ZINC06445930	0
database	7085	ZINC08400473	0
database	4624	ZINC00983483	0
database	6252	ZINC00987228	0
database	3000	ZINC02155878	0
database	5783	ZINC00688964	0
database	2322	ZINC00625888	0
database	7218	ZINC00668795	0
database	3237	ZINC00976118	0
database	4277	ZINC00984942	0

database	85	ZINC08442127	0
database	672	ZINC08997319	0
database	222	ZINC08442053	0
database	2164	ZINC01413500	0
database	4114	ZINC02979330	0
database	1986	ZINC02165142	0
database	3002	ZINC00645531	0
database	6555	ZINC00705137	0
database	4848	ZINC00845171	0
database	1472	ZINC08439818	0
database	3703	ZINC02311700	0
database	5974	ZINC08413383	0
database	4820	ZINC00995988	0
database	3738	ZINC08433173	0
database	518	ZINC00328721	0
database	438	ZINC00631361	0
database	5557	ZINC08424451	0
database	6547	ZINC01000505	0
database	4983	ZINC08427345	0
database	2176	ZINC01413495	0
database	3101	ZINC00883796	0
database	6142	ZINC08411460	0
database	3870	ZINC02350747	0
database	5491	ZINC08424912	0
database	1110	ZINC00702673	0
database	3669	ZINC01876399	0
database	4610	ZINC03670844	0

database	4821	ZINC00995985	0
database	6825	ZINC00687238	0
database	5497	ZINC02058382	0
database	2999	ZINC00645563	0
database	3197	ZINC06599869	0
database	1695	ZINC08439343	0
database	5530	ZINC00820398	0
database	1524	ZINC02171026	0
database	193	ZINC04167833	0
database	3841	ZINC01801677	0
database	6805	ZINC02146042	0
database	6960	ZINC02136088	0
database	4146	ZINC02744991	0
database	2655	ZINC08437659	0
database	7087	ZINC02252326	0
database	4578	ZINC00667464	0
database	4665	ZINC08430305	0
database	6876	ZINC00686670	0
database	1094	ZINC08440699	0
database	3679	ZINC00728048	0
database	1902	ZINC01019550	0
database	2440	ZINC08438206	0
database	6240	ZINC00987606	0
database	5796	ZINC08413985	0
database	2844	ZINC08437144	0
database	197	ZINC05919492	0
database	4659	ZINC08430310	0

database	4271	ZINC00845122	0
database	5959	ZINC08413403	0
database	4073	ZINC08432399	0
database	152	ZINC08442084	0
database	296	ZINC12416741	0
database	1903	ZINC01019551	0
database	5009	ZINC00058391	0
database	2540	ZINC00623497	0
database	3500	ZINC02070136	0
database	723	ZINC15952849	0
database	3260	ZINC05359859	0
database	364	ZINC06446299	0
database	3719	ZINC08433187	0
database	1032	ZINC00702715	0
database	3741	ZINC06996433	0
database	5353	ZINC08425653	0
database	109	ZINC08442109	0
database	2458	ZINC00624433	0
database	3174	ZINC00888222	0
database	3546	ZINC00677022	0
database	6557	ZINC19791803	0
database	6335	ZINC00985579	0
database	1914	ZINC00627368	0
database	2171	ZINC02164594	0
database	6503	ZINC19369482	0
database	198	ZINC05919492	0
database	6764	ZINC00687396	0

database	856	ZINC00850367	0
database	2683	ZINC00694356	0
database	387	ZINC02064394	0
database	469	ZINC00881562	0
database	1415	ZINC00480133	0
database	2485	ZINC08438007	0
database	6239	ZINC00987606	0
database	3650	ZINC08433397	0
database	2593	ZINC02161865	0
database	4918	ZINC08427601	0
database	4831	ZINC00982779	0
database	2190	ZINC01413499	0
database	5600	ZINC08417732	0
database	1668	ZINC08439384	0
database	4573	ZINC08430638	0
database	514	ZINC00881501	0
database	2397	ZINC00624832	0
database	5081	ZINC08426791	0
database	1989	ZINC01011837	0
database	6021	ZINC08413106	0
database	5154	ZINC08817408	0
database	531	ZINC15952844	0
database	6242	ZINC00987604	0
database	1322	ZINC17160093	0
database	6463	ZINC19312813	0
database	5098	ZINC08426774	0
database	1336	ZINC08440143	0

database	2673	ZINC06283023	0
database	4050	ZINC08432475	0
database	4862	ZINC02082145	0
database	2854	ZINC06144743	0
database	5688	ZINC08415486	0
database	4478	ZINC08431049	0
database	5677	ZINC08415497	0
database	2156	ZINC02164631	0
database	4921	ZINC08427579	0
database	218	ZINC13161526	0
database	3997	ZINC08432661	0
database	5135	ZINC08856047	0
database	4305	ZINC00997655	0
database	4572	ZINC08430638	0
database	3568	ZINC00675853	0
database	5455	ZINC08425064	0
database	3542	ZINC08435023	0
database	1406	ZINC08439973	0
database	6215	ZINC04473385	0
database	959	ZINC06445812	0
database	4643	ZINC08430349	0
database	1209	ZINC00666145	0
database	2813	ZINC08437187	0
database	7198	ZINC08400284	0
database	6398	ZINC08407898	0
database	4162	ZINC00668303	0
database	629	ZINC00857149	0

database	3903	ZINC08432888	0
database	6155	ZINC08411433	0
database	2668	ZINC00622664	0
database	2008	ZINC08438781	0
database	359	ZINC08441915	0
database	816	ZINC16956754	0
database	3009	ZINC08436921	0
database	1040	ZINC00702713	0
database	891	ZINC06556763	0
database	4619	ZINC00996333	0
database	1342	ZINC08440140	0
database	255	ZINC08442020	0
database	6721	ZINC00687455	0
database	1869	ZINC02167933	0
database	2645	ZINC08437668	0
database	2606	ZINC01011108	0
database	4622	ZINC08430371	0
database	1747	ZINC08439260	0
database	2564	ZINC01011191	0
database	609	ZINC15880060	0
database	4627	ZINC08430361	0
database	2731	ZINC08437429	0
database	1602	ZINC00717216	0
database	3969	ZINC02085913	0
database	7133	ZINC00676384	0
database	4585	ZINC00667218	0
database	4308	ZINC08828689	0

database	711	ZINC15884982	0
database	2187	ZINC01413498	0
database	888	ZINC05520252	0
database	6518	ZINC03103213	0
database	456	ZINC00881612	0
database	5679	ZINC08415491	0
database	490	ZINC00650714	0
database	2352	ZINC00625729	0
database	2386	ZINC08438460	0
database	6096	ZINC13147205	0
database	2553	ZINC00623324	0
database	6260	ZINC00987206	0
database	1074	ZINC00702698	0
database	2461	ZINC00624418	0
database	1760	ZINC03635668	0
database	2991	ZINC00645565	0
database	2287	ZINC08438674	0
database	3529	ZINC01953697	0
database	4234	ZINC08431777	0
database	744	ZINC00703052	0
database	1712	ZINC06270540	0
database	5137	ZINC08426628	0
database	3003	ZINC00645527	0
database	4069	ZINC00669305	0
database	3601	ZINC08434852	0
database	4723	ZINC08430218	0
database	683	ZINC00648858	0

database	1671	ZINC08439383	0
database	4066	ZINC02085471	0
database	4444	ZINC00984458	0
database	4941	ZINC01029930	0
database	1056	ZINC00702701	0
database	6269	ZINC00987156	0
database	6888	ZINC00686600	0
database	1627	ZINC08439529	0
database	946	ZINC08440967	0
database	373	ZINC02064405	0
database	3824	ZINC01016329	0
database	4011	ZINC08432577	0
database	326	ZINC08441953	0
database	827	ZINC08897821	0
database	4713	ZINC00983021	0
database	1089	ZINC00702690	0
database	6821	ZINC08400808	0
database	6942	ZINC01008315	0
database	3575	ZINC00675732	0
database	2002	ZINC00401042	0
database	5635	ZINC00713366	0
database	4102	ZINC08432295	0
database	664	ZINC06406305	0
database	6568	ZINC01022480	0
database	3647	ZINC00702232	0
database	3660	ZINC00730337	0
database	4671	ZINC08430295	0

database	4270	ZINC08431729	0
database	1589	ZINC08439601	0
database	4215	ZINC00632097	0
database	1796	ZINC02169708	0
database	3933	ZINC00249652	0
database	128	ZINC00664768	0
database	3193	ZINC06177172	0
database	484	ZINC00650985	0
database	604	ZINC15880061	0
database	6009	ZINC08413115	0
database	1847	ZINC08438990	0
database	5358	ZINC08425628	0
database	133	ZINC00664757	0
database	1401	ZINC08439979	0
database	4312	ZINC00997645	0
database	3976	ZINC01810304	0
database	6035	ZINC08413085	0
database	695	ZINC08441467	0
database	1305	ZINC08440200	0
database	6135	ZINC08411472	0
database	6799	ZINC00687325	0
database	6833	ZINC08400796	0
database	4785	ZINC08430124	0
database	366	ZINC08441911	0
database	2510	ZINC00624104	0
database	1011	ZINC00702730	0
database	4980	ZINC02901810	0

database	6475	ZINC02702312	0
database	4654	ZINC08430325	0
database	164	ZINC00664623	0
database	762	ZINC00703043	0
database	851	ZINC05313297	0
database	4427	ZINC00984558	0
database	4811	ZINC00845125	0
database	6954	ZINC08400662	0
database	3141	ZINC00907644	0
database	6706	ZINC00687504	0
database	6202	ZINC08411308	0
database	6120	ZINC08411515	0
database	2201	ZINC01011673	0
database	1824	ZINC02497221	0
database	6967	ZINC08400654	0
database	4857	ZINC02082198	0
database	1296	ZINC00702434	0
database	5277	ZINC00836256	0
database	3042	ZINC02155256	0
database	6182	ZINC08411403	0
database	2922	ZINC04149943	0
database	5601	ZINC08417714	0
database	605	ZINC15880062	0
database	2482	ZINC05985899	0
database	2554	ZINC01011373	0
database	5366	ZINC00708253	0
database	6222	ZINC08411251	0

database	2173	ZINC01413494	0
database	6521	ZINC00060451	0
database	248	ZINC08442026	0
database	830	ZINC08897821	0
database	2351	ZINC00625729	0
database	2076	ZINC01414784	0
database	2607	ZINC02161783	0
database	2900	ZINC09186824	0
database	2338	ZINC00625750	0
database	6271	ZINC00987152	0
database	506	ZINC00881536	0
database	7223	ZINC00755240	0
database	447	ZINC00881656	0
database	1080	ZINC08440708	0
database	3277	ZINC08435586	0
database	305	ZINC08441973	0
database	3939	ZINC08432831	0
database	4549	ZINC08430703	0
database	1407	ZINC08439973	0
database	2290	ZINC08438660	0
database	2750	ZINC12311320	0
database	2547	ZINC01854995	0
database	4524	ZINC00441956	0
database	1259	ZINC00970540	0
database	5440	ZINC00728827	0
database	1149	ZINC08440584	0
database	6600	ZINC19781721	0

database	29	ZINC18141403	0
database	1894	ZINC00627455	0
database	5891	ZINC08413865	0
database	5834	ZINC08413952	0
database	4956	ZINC00711743	0
database	3194	ZINC00976239	0
database	7216	ZINC00668824	0
database	4562	ZINC08430659	0
database	1070	ZINC08440712	0
database	3682	ZINC08433276	0
database	6655	ZINC00687670	0
database	6313	ZINC00985809	0
database	6005	ZINC08413115	0
database	2022	ZINC08438777	0
database	4914	ZINC08427614	0
database	1640	ZINC04113087	0
database	4281	ZINC08431719	0
database	3201	ZINC00976201	0
database	178	ZINC00664546	0
database	1663	ZINC08439395	0
database	5638	ZINC00692266	0
database	1117	ZINC00702674	0
database	2407	ZINC00624738	0
database	6092	ZINC04457875	0
database	2305	ZINC01019485	0
database	2395	ZINC00624847	0
database	3273	ZINC09280118	0

database	5094	ZINC08426778	0
database	4761	ZINC00729391	0
database	4313	ZINC02084413	0
database	2448	ZINC02204515	0
database	1165	ZINC00666443	0
database	3280	ZINC08435585	0
database	6877	ZINC00686666	0
database	6740	ZINC00687424	0
database	3117	ZINC00862097	0
database	5589	ZINC02154432	0
database	2200	ZINC01011676	0
database	2180	ZINC08438764	0
database	5741	ZINC08414848	0
database	1814	ZINC00683837	0
database	6771	ZINC00687381	0
database	4430	ZINC08431306	0
database	208	ZINC09210009	0
database	2040	ZINC01414772	0
database	1789	ZINC08439179	0
database	6173	ZINC06296580	0
database	4824	ZINC08430011	0
database	416	ZINC01126049	0
database	2465	ZINC01011467	0
database	4538	ZINC08430753	0
database	151	ZINC08442084	0
database	835	ZINC08441259	0
database	4525	ZINC02740371	0

database	2033	ZINC01414770	0
database	2532	ZINC08437851	0
database	3032	ZINC00645500	0
database	5779	ZINC02152227	0
database	485	ZINC00650985	0
database	5769	ZINC06215464	0
database	5276	ZINC00836256	0
database	6095	ZINC13147205	0
database	6822	ZINC06177556	0
database	1468	ZINC08439872	0
database	6980	ZINC00677021	0
database	1911	ZINC08438900	0
database	2487	ZINC08438003	0
database	77	ZINC00633944	0
database	3836	ZINC01895996	0
database	3944	ZINC08432795	0
database	3536	ZINC02069274	0
database	2	ZINC09365179	0
database	4345	ZINC00703575	0
database	4222	ZINC06154142	0
database	4342	ZINC00703577	0
database	7089	ZINC02252327	0
database	6882	ZINC00686615	0
database	4157	ZINC08432097	0
database	2752	ZINC02075274	0
database	1812	ZINC08439136	0
database	2672	ZINC04167654	0

database	3509	ZINC00678269	0
database	1539	ZINC08439704	0
database	140	ZINC08442089	0
database	2537	ZINC00623556	0
database	536	ZINC15952845	0
database	2812	ZINC08437187	0
database	1145	ZINC08440584	0
database	4563	ZINC00996550	0
database	2026	ZINC08438776	0
database	5274	ZINC00836245	0
database	1980	ZINC01011857	0
database	4361	ZINC00703556	0
database	1691	ZINC08439348	0
database	6921	ZINC01008436	0
database	645	ZINC00863329	0
database	1160	ZINC00702625	0
database	3286	ZINC19790623	0
database	726	ZINC00862590	0
database	2955	ZINC06144543	0
database	6866	ZINC00686698	0
database	1159	ZINC08440583	0
database	1969	ZINC02165251	0
database	5189	ZINC01016496	0
database	4810	ZINC08430088	0
database	4263	ZINC02742556	0
database	265	ZINC08442009	0
database	1520	ZINC02171084	0

database	3385	ZINC08435512	0
database	4558	ZINC08430663	0
database	4435	ZINC08431296	0
database	76	ZINC00633946	0
database	1652	ZINC08439418	0
database	370	ZINC00882033	0
database	6113	ZINC08411532	0
database	626	ZINC00971723	0
database	3018	ZINC08436913	0
database	3964	ZINC08432759	0
database	73	ZINC00633962	0
database	4008	ZINC00710212	0
database	6394	ZINC00706431	0
database	5930	ZINC08413446	0
database	2292	ZINC08438660	0
database	4508	ZINC00029092	0
database	3069	ZINC06195126	0
database	6836	ZINC08400794	0
database	5583	ZINC13549398	0
database	4510	ZINC00420692	0
database	3625	ZINC00974478	0
database	3026	ZINC00645507	0
database	2990	ZINC00645564	0
database	6525	ZINC19370925	0
database	2790	ZINC00646483	0
database	6093	ZINC08411675	0
database	4394	ZINC08431434	0

database	6724	ZINC00687446	0
database	4368	ZINC00703552	0
database	4040	ZINC08432501	0
database	285	ZINC08836443	0
database	1763	ZINC02503433	0
database	6250	ZINC00987227	0
database	6673	ZINC00687618	0
database	7146	ZINC00675685	0
database	5117	ZINC08426696	0
database	1553	ZINC08439659	0
database	692	ZINC08441470	0
database	356	ZINC08441917	0
database	6875	ZINC00686673	0
database	846	ZINC02380267	0
database	1604	ZINC08439593	0
database	5281	ZINC02060611	0
database	1168	ZINC00666393	0
database	708	ZINC08441455	0
database	80	ZINC00977563	0
database	5272	ZINC00836245	0
database	5728	ZINC08415115	0
database	4977	ZINC08427361	0
database	3765	ZINC02106710	0
database	4684	ZINC00665302	0
database	4681	ZINC00665321	0
database	6943	ZINC08400680	0
database	5089	ZINC08426780	0

database	4473	ZINC00997067	0
database	498	ZINC00703121	0
database	3455	ZINC00662855	0
database	3649	ZINC08433412	0
database	5044	ZINC08426855	0
database	2179	ZINC08438764	0
database	5880	ZINC08413878	0
database	82	ZINC08442128	0
database	1278	ZINC08440216	0
database	4304	ZINC00997660	0
database	8	ZINC19799526	0
database	983	ZINC00702761	0
database	276	ZINC01107286	0
database	4107	ZINC00985727	0
database	2137	ZINC08438769	0
database	6893	ZINC02143200	0
database	5757	ZINC08414672	0
database	6738	ZINC00687426	0
database	3639	ZINC06904335	0
database	3756	ZINC08742400	0
database	6926	ZINC08400708	0
database	3850	ZINC06154170	0
database	1077	ZINC08440709	0
database	2959	ZINC00645581	0
database	283	ZINC08836444	0
database	4823	ZINC08430011	0
database	3770	ZINC01017575	0

database	7068	ZINC02158022	0
database	3094	ZINC00676207	0
database	1207	ZINC08440458	0
database	4095	ZINC01794771	0
database	7053	ZINC08400509	0
database	6843	ZINC08400784	0
database	4996	ZINC08427301	0
database	3279	ZINC08435585	0
database	6579	ZINC00126997	0
database	4124	ZINC00872120	0
database	6689	ZINC00687575	0
database	6677	ZINC00687613	0
database	6674	ZINC00687616	0
database	2720	ZINC08437441	0
database	1884	ZINC00627481	0
database	6195	ZINC08411329	0
database	779	ZINC00853651	0
database	6869	ZINC00686690	0
database	507	ZINC00881534	0
database	2966	ZINC00645574	0
database	5525	ZINC08424635	0
database	574	ZINC01019940	0
database	5001	ZINC03193943	0
database	1448	ZINC09109829	0
database	2986	ZINC13122228	0
database	426	ZINC00881833	0
database	4725	ZINC00983013	0

database	666	ZINC06406732	0
database	35	ZINC08442293	0
database	6823	ZINC08400807	0
database	6603	ZINC00704834	0
database	275	ZINC01107286	0
database	2484	ZINC08395141	0
database	2658	ZINC00622796	0
database	4319	ZINC08431535	0
database	6477	ZINC08405929	0
database	5451	ZINC08425101	0
database	4771	ZINC08430143	0
database	3014	ZINC08436914	0
database	4079	ZINC00669027	0
database	4732	ZINC06287758	0
database	5923	ZINC08413517	0
database	5010	ZINC00711065	0
database	742	ZINC00703052	0
database	4445	ZINC08431228	0
database	3458	ZINC00975338	0
database	5801	ZINC08413985	0
database	5548	ZINC00707248	0
database	7208	ZINC08400266	0
database	436	ZINC08441875	0
database	4485	ZINC02742177	0
database	6891	ZINC00686591	0
database	7164	ZINC02135480	0
database	4358	ZINC00703555	0

database	677	ZINC08441495	0
database	3784	ZINC02106595	0
database	5015	ZINC08427102	0
database	2037	ZINC01414771	0
database	4836	ZINC08429974	0
database	3931	ZINC08432846	0
database	6719	ZINC00687457	0
database	7174	ZINC08400389	0
database	6716	ZINC00687464	0
database	4210	ZINC00997875	0
database	3751	ZINC09301884	0
database	3767	ZINC02106709	0
database	3573	ZINC00430532	0
database	4112	ZINC02979330	0
database	3839	ZINC00719360	0
database	5005	ZINC00711111	0
database	3697	ZINC09271701	0
database	7178	ZINC00675309	0
database	3936	ZINC08432836	0
database	1836	ZINC02497236	0
database	2857	ZINC00646445	0
database	3510	ZINC08435118	0
database	4209	ZINC02183409	0
database	1959	ZINC08438856	0
database	5733	ZINC08415062	0
database	5943	ZINC08413415	0
database	4466	ZINC00442230	0

database	3537	ZINC08435027	0
database	3966	ZINC08432754	0
database	7159	ZINC02135518	0
database	1950	ZINC08438864	0
database	3033	ZINC00645500	0
database	3395	ZINC02071423	0
database	3285	ZINC19790623	0
database	5541	ZINC00855482	0
database	5179	ZINC08426375	0
database	6997	ZINC08400611	0
database	2793	ZINC08437242	0
database	3323	ZINC06177076	0
database	2463	ZINC08438101	0
database	4018	ZINC00986094	0
database	44	ZINC08442277	0
database	3425	ZINC08435392	0
database	4868	ZINC09110317	0
database	7095	ZINC08400467	0
database	2178	ZINC02164575	0
database	4187	ZINC08431895	0
database	5128	ZINC06245045	0
database	1889	ZINC00627464	0
database	7212	ZINC08400237	0
database	4553	ZINC00996573	0
database	1646	ZINC00643337	0
database	3977	ZINC08432706	0
database	4826	ZINC16975676	0

database	3861	ZINC08432989	0
database	4726	ZINC02082972	0
database	2850	ZINC06144743	0
database	6936	ZINC02142324	0
database	2304	ZINC01019485	0
database	1715	ZINC08439310	0
database	188	ZINC04167843	0
database	2796	ZINC00646452	0
database	3950	ZINC08432772	0
database	6164	ZINC08411415	0
database	5108	ZINC08426766	0
database	83	ZINC08442128	0
database	3078	ZINC00883748	0
database	3456	ZINC00975370	0
database	5804	ZINC08413985	0
database	1846	ZINC08438990	0
database	3428	ZINC00845542	0
database	1398	ZINC08439994	0
database	1996	ZINC02165071	0
database	1707	ZINC02987093	0
database	6948	ZINC08400672	0
database	3109	ZINC00915380	0
database	6003	ZINC08413118	0
database	1829	ZINC08439087	0
database	6531	ZINC19370921	0
database	1300	ZINC00702428	0
database	1575	ZINC08439616	0

database	1882	ZINC00627555	0
database	3158	ZINC00915437	0
database	5697	ZINC08415481	0
database	2358	ZINC00625740	0
database	4752	ZINC08430164	0
database	4479	ZINC00126462	0
database	4170	ZINC00706993	0
database	4217	ZINC08431833	0
database	272	ZINC08442006	0
database	3076	ZINC00882914	0
database	3300	ZINC08818093	0
database	4367	ZINC00703552	0
database	2276	ZINC08438681	0
database	5841	ZINC08413942	0
database	2385	ZINC00625325	0
database	5537	ZINC03666980	0
database	3115	ZINC06444464	0
database	4950	ZINC00711748	0
database	6844	ZINC00686847	0
database	5017	ZINC08427099	0
database	1817	ZINC00683820	0
database	5304	ZINC00969616	0
database	3548	ZINC08435017	0
database	6757	ZINC00687403	0
database	671	ZINC06406737	0
database	1101	ZINC05918675	0
database	2740	ZINC08437421	0

database	4801	ZINC00729125	0
database	3457	ZINC00662744	0
database	7067	ZINC02158022	0
database	2160	ZINC01413517	0
database	3865	ZINC08432981	0
database	936	ZINC08441010	0
database	6860	ZINC00686731	0
database	3250	ZINC00864422	0
database	2060	ZINC01413468	0
database	5574	ZINC00827399	0
database	947	ZINC08440965	0
database	4680	ZINC02065670	0
database	136	ZINC00664736	0
database	6424	ZINC08407207	0
database	570	ZINC08441629	0
database	6165	ZINC08411415	0
database	2348	ZINC00625722	0
database	2657	ZINC00622793	0
database	5853	ZINC08413917	0
database	5181	ZINC01016673	0
database	3582	ZINC00675498	0
database	6465	ZINC00705792	0
database	2563	ZINC02999763	0
database	4972	ZINC02312181	0
database	4303	ZINC00997670	0
database	4431	ZINC08431305	0
database	1310	ZINC00702426	0

database	332	ZINC08441941	0
database	92	ZINC08442119	0
database	585	ZINC08441623	0
database	644	ZINC00863328	0
database	5704	ZINC08415476	0
database	1379	ZINC08440078	0
database	5319	ZINC00340343	0
database	5323	ZINC00647608	0
database	4178	ZINC08431931	0
database	4470	ZINC08431155	0
database	6840	ZINC01008658	0
database	1371	ZINC08440094	0
database	6098	ZINC13147206	0
database	999	ZINC05921386	0
database	2428	ZINC08438244	0
database	1120	ZINC08440677	0
database	3499	ZINC02900087	0
database	3486	ZINC08435211	0
database	6185	ZINC08411388	0
database	3410	ZINC08435455	0
database	1511	ZINC13545842	0
database	2815	ZINC08437179	0
database	5138	ZINC00709680	0
database	6312	ZINC00985809	0
database	2559	ZINC02162241	0
database	5500	ZINC00707571	0
database	3131	ZINC00793598	0

database	5987	ZINC08413236	0
database	5380	ZINC04196466	0
database	6457	ZINC00705797	0
database	5559	ZINC01018035	0
database	6314	ZINC00985810	0
database	5768	ZINC08414463	0
database	189	ZINC04166616	0
database	749	ZINC08441403	0
database	413	ZINC01126045	0
database	2419	ZINC08438288	0
database	6814	ZINC08400811	0
database	1844	ZINC02168617	0
database	2669	ZINC04167655	0
database	7204	ZINC01001942	0
database	5576	ZINC01251163	0
database	5326	ZINC00708348	0
database	4896	ZINC08427629	0
database	1479	ZINC08439792	0
database	3184	ZINC00907219	0
database	3734	ZINC00724096	0
database	4226	ZINC08431798	0
database	2777	ZINC00646486	0
database	64	ZINC00633984	0
database	1735	ZINC08439284	0
database	3362	ZINC00047418	0
database	3081	ZINC00884347	0
database	288	ZINC00660155	0

database	6504	ZINC19369482	0
database	3430	ZINC06177847	0
database	5261	ZINC08426067	0
database	4813	ZINC02082432	0
database	5917	ZINC08413520	0
database	4240	ZINC06232543	0
database	4191	ZINC00706907	0
database	2524	ZINC00623808	0
database	2869	ZINC00646443	0
database	6584	ZINC08403889	0
database	7138	ZINC08400418	0
database	1933	ZINC00627343	0
database	1726	ZINC00622047	0
database	2526	ZINC00623797	0
database	3442	ZINC02070797	0
database	4070	ZINC00669287	0
database	5024	ZINC00235816	0
database	1690	ZINC08439353	0
database	2627	ZINC08437688	0
database	2851	ZINC06144743	0
database	2921	ZINC08436957	0
database	3449	ZINC08435312	0
database	6274	ZINC04167867	0
database	5447	ZINC00728810	0
database	1900	ZINC00627449	0
database	845	ZINC05013440	0
database	5085	ZINC00710527	0

database	390	ZINC02064389	0
database	2172	ZINC01413494	0
database	3558	ZINC00974814	0
database	1519	ZINC00630105	0
database	6013	ZINC08413110	0
database	6951	ZINC02142082	0
database	6245	ZINC00987595	0
database	631	ZINC00857147	0
database	2685	ZINC08437534	0
database	4147	ZINC02281622	0
database	564	ZINC00878047	0
database	6208	ZINC08411284	0
database	5553	ZINC08424473	0
database	2106	ZINC01414731	0
database	4374	ZINC00703540	0
database	2767	ZINC00646561	0
database	5535	ZINC00861845	0
database	7192	ZINC00685524	0
database	6527	ZINC19370925	0
database	3387	ZINC08435507	0
database	4838	ZINC08429974	0
database	1387	ZINC00276456	0
database	6061	ZINC08413048	0
database	651	ZINC13123301	0
database	3539	ZINC00194938	0
database	4132	ZINC08432201	0
database	777	ZINC08441378	0

database	5218	ZINC09271507	0
database	1813	ZINC01012760	0
database	2056	ZINC08438775	0
database	6048	ZINC08413066	0
database	4144	ZINC08432167	0
database	1533	ZINC02170984	0
database	6180	ZINC08411403	0
database	6746	ZINC00687414	0
database	6150	ZINC08411444	0
database	3485	ZINC02070324	0
database	3490	ZINC00655244	0
database	1030	ZINC00702726	0
database	7061	ZINC00920466	0
database	4542	ZINC12462012	0
database	5593	ZINC00692998	0
database	3611	ZINC02067899	0
database	2712	ZINC08437463	0
database	7106	ZINC08400458	0
database	4065	ZINC02299334	0
database	7205	ZINC00668882	0
database	840	ZINC00851289	0
database	4274	ZINC00984944	0
database	3531	ZINC08435037	0
database	5661	ZINC00691663	0
database	812	ZINC08441295	0
database	2918	ZINC08436961	0
database	3192	ZINC06177403	0

database	7215	ZINC08400228	0
database	1890	ZINC00627467	0
database	1412	ZINC05728903	0
database	774	ZINC08441386	0
database	3030	ZINC08436903	0
database	7050	ZINC08400512	0
database	6017	ZINC08413110	0
database	921	ZINC08441027	0
database	3572	ZINC00675748	0
database	6663	ZINC00687662	0
database	4459	ZINC08431189	0
database	955	ZINC06300225	0
database	775	ZINC08441383	0
database	7175	ZINC00675408	0
database	5502	ZINC01024407	0
database	3958	ZINC00986405	0
database	1641	ZINC04113087	0
database	1958	ZINC08438858	0
database	4254	ZINC00870363	0
database	2617	ZINC00622925	0
database	5505	ZINC00854811	0
database	4258	ZINC00845216	0
database	1587	ZINC08439611	0
database	2831	ZINC00625689	0
database	7136	ZINC00675723	0
database	5464	ZINC00728672	0
database	2128	ZINC01413486	0

database	1580	ZINC08439613	0
database	2690	ZINC01010983	0
database	5156	ZINC08817407	0
database	2795	ZINC08437236	0
database	4399	ZINC08431418	0
database	2770	ZINC00693953	0
database	2937	ZINC00645591	0
database	715	ZINC15884978	0
database	3454	ZINC00662865	0
database	7209	ZINC02135266	0
database	3172	ZINC08435619	0
database	5410	ZINC00729199	0
database	4632	ZINC08430357	0
database	2562	ZINC00623269	0
database	5835	ZINC08413952	0
database	4679	ZINC02065669	0
database	1952	ZINC00627094	0
database	3232	ZINC06442929	0
database	3584	ZINC05685816	0
database	2399	ZINC00624810	0
database	6702	ZINC00687525	0
database	620	ZINC08441595	0
database	4291	ZINC00845702	0
database	223	ZINC00633806	0
database	1660	ZINC06321511	0
database	2947	ZINC06144562	0
database	55	ZINC00634115	0

database	615	ZINC08441600	0
database	482	ZINC00703147	0
database	6288	ZINC08410672	0
database	5115	ZINC08426701	0
database	2396	ZINC00624842	0
database	3629	ZINC00974457	0
database	3804	ZINC00723825	0
database	1982	ZINC02165195	0
database	2861	ZINC00646442	0
database	6393	ZINC02149686	0
database	647	ZINC13123302	0
database	3137	ZINC00838424	0
database	6154	ZINC08411438	0
database	2551	ZINC08437770	0
database	3608	ZINC08434839	0
database	1410	ZINC04002657	0
database	1183	ZINC00702582	0
database	5228	ZINC08426172	0
database	4064	ZINC00669356	0
database	6636	ZINC00687845	0
database	3006	ZINC00645521	0
database	893	ZINC08441076	0
database	2014	ZINC02164913	0
database	3704	ZINC08433224	0
database	5507	ZINC02309977	0
database	657	ZINC13123054	0
database	3326	ZINC06177076	0

database	3843	ZINC00719357	0
database	5480	ZINC08424944	0
database	4449	ZINC09065134	0
database	5584	ZINC08417899	0
database	5220	ZINC09271506	0
database	4807	ZINC08430101	0
database	667	ZINC06406732	0
database	1114	ZINC00702674	0
database	2114	ZINC01414763	0
database	3965	ZINC01896150	0
database	430	ZINC00881828	0
database	4509	ZINC00667918	0
database	3470	ZINC00655611	0
database	3443	ZINC00627140	0
database	3484	ZINC08435234	0
database	3959	ZINC00986404	0
database	4082	ZINC00668933	0
database	6909	ZINC01008535	0
database	5361	ZINC00708257	0
database	2431	ZINC00669302	0
database	1935	ZINC00627346	0
database	4926	ZINC08427531	0
database	6613	ZINC00704798	0
database	6622	ZINC08402538	0
database	7149	ZINC08400405	0
database	5521	ZINC00627292	0
database	4945	ZINC00711771	0

database	3974	ZINC00670657	0
database	904	ZINC08441044	0
database	5486	ZINC08424915	0
database	923	ZINC08441023	0
database	1104	ZINC05918668	0
database	2142	ZINC01413488	0
database	1998	ZINC02165060	0
database	1244	ZINC08440332	0
database	6988	ZINC01008246	0
database	3887	ZINC02088003	0
database	5205	ZINC08426280	0
database	4480	ZINC08431045	0
database	7155	ZINC08400397	0
database	1701	ZINC08439334	0
database	2717	ZINC08437442	0
database	3057	ZINC08436815	0
database	789	ZINC00853619	0
database	3986	ZINC08432687	0
database	404	ZINC00881982	0
database	5210	ZINC08426215	0
database	6787	ZINC00687345	0
database	7022	ZINC00676717	0
database	5111	ZINC08426765	0
database	941	ZINC08440990	0
database	4360	ZINC00703556	0
database	5442	ZINC00728821	0
database	4963	ZINC00711719	0

database	1273	ZINC00702446	0
database	2336	ZINC08438562	0
database	4827	ZINC09110716	0
database	6792	ZINC00687338	0
database	6643	ZINC00687792	0
database	1391	ZINC15906697	0
database	2366	ZINC00625470	0
database	7011	ZINC06682076	0
database	461	ZINC00630488	0
database	3905	ZINC08432887	0
database	1948	ZINC08438867	0
database	910	ZINC00850105	0
database	3951	ZINC00986439	0
database	1816	ZINC08439124	0
database	3888	ZINC01009696	0
database	6306	ZINC00985844	0
database	1271	ZINC00702446	0
database	4078	ZINC00669049	0
database	331	ZINC08441941	0
database	3776	ZINC00723991	0
database	2004	ZINC02184550	0
database	6318	ZINC00985635	0
database	2781	ZINC00646487	0
database	4433	ZINC08431303	0
database	1315	ZINC00702427	0
database	7001	ZINC02135963	0
database	3523	ZINC00195057	0

database	60	ZINC00633999	0
database	4789	ZINC00664670	0
database	5105	ZINC08426766	0
database	967	ZINC08440877	0
database	340	ZINC08441930	0
database	6938	ZINC02142298	0
database	3358	ZINC08435556	0
database	7049	ZINC08400514	0
database	6212	ZINC04473387	0
database	1212	ZINC08440415	0
database	6200	ZINC08411315	0
database	6670	ZINC00687626	0
database	4115	ZINC02083487	0
database	3920	ZINC00223273	0
database	2113	ZINC02164692	0
database	4293	ZINC00845638	0
database	5723	ZINC08415436	0
database	6263	ZINC00987198	0
database	1687	ZINC08439362	0
database	5298	ZINC00507688	0
database	4029	ZINC02484243	0
database	2874	ZINC00148162	0
database	6765	ZINC00687395	0
database	6918	ZINC01008442	0
database	3040	ZINC02155281	0
database	3472	ZINC00655595	0
database	5052	ZINC08426825	0

database	3949	ZINC01801665	0
database	5122	ZINC08426677	0
database	3869	ZINC02350747	0
database	4805	ZINC00664557	0
database	6337	ZINC00985574	0
database	6470	ZINC08406075	0
database	5607	ZINC00713388	0
database	3271	ZINC00625938	0
database	6589	ZINC08403635	0
database	1247	ZINC00659536	0
database	4971	ZINC02312180	0
database	357	ZINC08441917	0
database	2895	ZINC08437022	0
database	4338	ZINC00703585	0
database	1190	ZINC08440509	0
database	3866	ZINC01011316	0
database	6388	ZINC08407957	0
database	5832	ZINC08413954	0
database	834	ZINC08441259	0
database	5175	ZINC08426397	0
database	1201	ZINC02062354	0
database	1187	ZINC08440510	0
database	3077	ZINC00883750	0
database	419	ZINC00881866	0
database	7000	ZINC02141803	0
database	6340	ZINC00985561	0
database	6852	ZINC08400777	0

database	6159	ZINC08411428	0
database	5045	ZINC08426849	0
database	5588	ZINC00184011	0
database	4786	ZINC00664705	0
database	6530	ZINC19370919	0
database	2586	ZINC08437719	0
database	1557	ZINC08439654	0
database	132	ZINC00664754	0
database	1163	ZINC08440552	0
database	2789	ZINC00646482	0
database	2927	ZINC02497212	0
database	6680	ZINC00687603	0
database	6478	ZINC08405929	0
database	2561	ZINC00623272	0
database	3047	ZINC08436845	0
database	3051	ZINC00616546	0
database	86	ZINC00633899	0
database	1047	ZINC00702709	0
database	1956	ZINC00627056	0
database	2802	ZINC08437215	0
database	6111	ZINC08411532	0
database	895	ZINC06445734	0
database	1230	ZINC08440364	0
database	2398	ZINC00624813	0
database	5314	ZINC08425788	0
database	2775	ZINC01805329	0
database	4675	ZINC00665333	0

database	2473	ZINC08438076	0
database	2635	ZINC00622868	0
database	5238	ZINC02060712	0
database	576	ZINC08441627	0
database	3087	ZINC00884274	0
database	3613	ZINC02067899	0
database	5990	ZINC04718185	0
database	5184	ZINC00839005	0
database	950	ZINC00648073	0
database	1436	ZINC13286748	0
database	2442	ZINC08438200	0
database	1352	ZINC00854709	0
database	2612	ZINC00623005	0
database	5665	ZINC00691603	0
database	3858	ZINC00715656	0
database	6086	ZINC08411694	0
database	1049	ZINC00702710	0
database	5515	ZINC00192819	0
database	2302	ZINC08438644	0
database	4967	ZINC08427462	0
database	790	ZINC08441316	0
database	322	ZINC08441956	0
database	3528	ZINC01881650	0
database	4711	ZINC02065607	0
database	2838	ZINC08437153	0
database	2349	ZINC00625722	0
database	2891	ZINC00646195	0

database	914	ZINC08441036	0
database	640	ZINC15885021	0
database	6432	ZINC08406623	0
database	158	ZINC08442081	0
database	2015	ZINC09374642	0
database	6045	ZINC08413066	0
database	6158	ZINC08411428	0
database	1440	ZINC00721238	0
database	3732	ZINC00727870	0
database	4790	ZINC08430119	0
database	160	ZINC05921073	0
database	4325	ZINC00292433	0
database	6350	ZINC00751985	0
database	5883	ZINC08413868	0
database	600	ZINC08441614	0
database	4720	ZINC06027641	0
database	6798	ZINC00687326	0
database	2354	ZINC00669594	0
database	91	ZINC08442120	0
database	84	ZINC08442127	0
database	2257	ZINC08438693	0
database	329	ZINC08441943	0
database	5705	ZINC08415476	0
database	2761	ZINC02158929	0
database	4664	ZINC08430307	0
database	2552	ZINC00623328	0
database	4004	ZINC01895861	0

database	1146	ZINC08440584	0
database	1629	ZINC00643346	0
database	2853	ZINC06144743	0
database	5084	ZINC08426784	0
database	2003	ZINC02184550	0
database	2769	ZINC01805305	0
database	4514	ZINC00997003	0
database	3048	ZINC08436844	0
database	6347	ZINC00985556	0
database	1399	ZINC02409214	0
database	5615	ZINC00713377	0
database	7141	ZINC00358883	0
database	6322	ZINC00985623	0
database	270	ZINC08442006	0
database	1616	ZINC08439556	0
database	3709	ZINC08433222	0
database	291	ZINC00660158	0
database	7173	ZINC02135392	0
database	6397	ZINC08407902	0
database	4275	ZINC00984944	0
database	486	ZINC00650986	0
database	5558	ZINC08424430	0
database	6839	ZINC06288215	0
database	221	ZINC08442053	0
database	1856	ZINC08438967	0
database	2567	ZINC01011181	0
database	6884	ZINC00686606	0

database	7005	ZINC00676807	0
database	4499	ZINC08430958	0
database	4155	ZINC02745107	0
database	6130	ZINC08411500	0
database	2535	ZINC08437837	0
database	5182	ZINC01016673	0
database	962	ZINC06445684	0
database	2638	ZINC00622873	0
database	5758	ZINC08414662	0
database	3806	ZINC00723825	0
database	3411	ZINC08435455	0
database	2972	ZINC00645570	0
database	1897	ZINC00627441	0
database	792	ZINC08441316	0
database	3713	ZINC08433219	0
database	2371	ZINC08438531	0
database	7088	ZINC02252327	0
database	3	ZINC19166762	0
database	4545	ZINC13893793	0
database	6607	ZINC00013213	0
database	5466	ZINC08424961	0
database	3417	ZINC08435421	0
database	5404	ZINC00199681	0
database	7135	ZINC00675729	0
database	6440	ZINC18043169	0
database	5695	ZINC08415481	0
database	5029	ZINC08426999	0

database	7179	ZINC02135302	0
database	3173	ZINC00318435	0
database	2131	ZINC01413487	0
database	3202	ZINC08435611	0
database	1186	ZINC08440510	0
database	7189	ZINC06142731	0
database	669	ZINC06406308	0
database	513	ZINC00881503	0
database	1325	ZINC17160093	0
database	5209	ZINC08426278	0
database	177	ZINC00664574	0
database	6346	ZINC00985556	0
database	1700	ZINC00622382	0
database	2092	ZINC01414751	0
database	5286	ZINC08426008	0
database	385	ZINC08441900	0
database	6368	ZINC08409920	0
database	5653	ZINC08416873	0
database	1167	ZINC00666412	0
database	2566	ZINC01011185	0
database	2404	ZINC08438401	0
database	3041	ZINC02155266	0
database	961	ZINC06445778	0
database	6743	ZINC00687421	0
database	1681	ZINC00628603	0
database	5646	ZINC08417198	0
database	4327	ZINC08431519	0

database	5857	ZINC08413894	0
database	2819	ZINC08437174	0
database	5065	ZINC08426812	0
database	2780	ZINC00646487	0
database	3402	ZINC08435476	0
database	1913	ZINC00627368	0
database	3263	ZINC00862459	0
database	1411	ZINC05728874	0
database	3238	ZINC00976117	0
database	4387	ZINC00845145	0
database	945	ZINC00970849	0
database	1710	ZINC08439318	0
database	2754	ZINC00711910	0
database	6759	ZINC00687401	0
database	7060	ZINC08400495	0
database	2975	ZINC00645571	0
database	5354	ZINC08425651	0
database	6999	ZINC02135976	0
database	6175	ZINC08411403	0
database	6170	ZINC08411415	0
database	1202	ZINC08440462	0
database	2216	ZINC08438704	0
database	1603	ZINC00717216	0
database	6400	ZINC08407890	0
database	5203	ZINC08426280	0
database	1639	ZINC04113087	0
database	2910	ZINC00693785	0

database	3183	ZINC08435615	0
database	3565	ZINC05687815	0
database	6294	ZINC06406878	0
database	4464	ZINC08431174	0
database	5007	ZINC00711101	0
database	3398	ZINC06473864	0
database	5249	ZINC00780341	0
database	2313	ZINC08438568	0
database	3967	ZINC08432748	0
database	1612	ZINC00717196	0
database	934	ZINC00970877	0
database	731	ZINC00703050	0
database	4860	ZINC08429897	0
database	4551	ZINC08430698	0
database	5811	ZINC08413980	0
database	4185	ZINC08431906	0
database	441	ZINC08441870	0
database	6687	ZINC00687585	0
database	1947	ZINC00627114	0
database	6115	ZINC08411529	0
database	2602	ZINC01011117	0
database	3860	ZINC08432996	0
database	1064	ZINC00702696	0
database	7213	ZINC08400235	0
database	2508	ZINC00624109	0
database	6516	ZINC00705658	0
database	5914	ZINC08413520	0

database	546	ZINC02064361	0
database	2053	ZINC01414773	0
database	3494	ZINC00285987	0
database	2954	ZINC06144543	0
database	1039	ZINC00702718	0
database	3129	ZINC06658424	0
database	6913	ZINC02142789	0
database	6132	ZINC08411476	0
database	3215	ZINC00867607	0
database	4858	ZINC08429915	0
database	2341	ZINC00625755	0
database	4694	ZINC08430249	0
database	403	ZINC02064378	0
database	4784	ZINC08430125	0
database	5774	ZINC08414436	0
database	3257	ZINC00625459	0
database	6713	ZINC00687470	0
database	325	ZINC08441953	0
database	2762	ZINC00646567	0
database	5875	ZINC08413885	0
database	7066	ZINC02158027	0
database	7062	ZINC00920466	0
database	2262	ZINC01019496	0
database	4049	ZINC02085505	0
database	3152	ZINC00886816	0
database	1848	ZINC08438987	0
database	6361	ZINC12563589	0

database	2089	ZINC01011747	0
database	1251	ZINC08440246	0
database	5217	ZINC09271507	0
database	5362	ZINC08425617	0
database	5067	ZINC08426812	0
database	4221	ZINC00632045	0
database	719	ZINC15952848	0
database	1870	ZINC02502143	0
database	6107	ZINC08411537	0
database	1684	ZINC00381730	0
database	394	ZINC02064384	0
database	3364	ZINC02071920	0
database	5063	ZINC08426813	0
database	4321	ZINC00822717	0
database	442	ZINC08441870	0
database	5058	ZINC00710619	0
database	6408	ZINC01023130	0
database	7092	ZINC08400468	0
database	4061	ZINC00669375	0
database	1106	ZINC00632059	0
database	2603	ZINC01011111	0
database	4674	ZINC00665340	0
database	7003	ZINC01008226	0
database	4401	ZINC08431414	0
database	307	ZINC08441972	0
database	2828	ZINC00625693	0
database	648	ZINC13123302	0

database	3795	ZINC06144470	0
database	1250	ZINC08440257	0
database	4396	ZINC06142255	0
database	238	ZINC08442036	0
database	6711	ZINC00687472	0
database	6436	ZINC01022876	0
database	6184	ZINC08411395	0
database	6588	ZINC08403641	0
database	3344	ZINC02072006	0
database	6749	ZINC00687410	0
database	5356	ZINC00708286	0
database	3695	ZINC17163315	0
database	7096	ZINC08400467	0
database	6574	ZINC00051436	0
database	4765	ZINC00664819	0
database	3680	ZINC00728050	0
database	6697	ZINC00687551	0
database	5199	ZINC08426283	0
database	2630	ZINC00622878	0
database	4944	ZINC08427484	0
database	2425	ZINC02163892	0
database	4488	ZINC08431007	0
database	5201	ZINC08426283	0
database	4491	ZINC08430987	0
database	7139	ZINC08400417	0
database	769	ZINC00703045	0
database	3919	ZINC08432870	0

database	2890	ZINC00646194	0
database	6958	ZINC08400659	0
database	4113	ZINC02979330	0
database	5347	ZINC08425660	0
database	730	ZINC00862490	0
database	3467	ZINC02070503	0
database	5351	ZINC00783483	0
database	903	ZINC08441046	0
database	2248	ZINC06445928	0
database	3110	ZINC00890083	0
database	2222	ZINC00626544	0
database	589	ZINC02064354	0
database	6944	ZINC01008306	0
database	4762	ZINC08430153	0
database	6631	ZINC19014718	0
database	3008	ZINC08436921	0
database	6253	ZINC00987228	0
database	6289	ZINC08410672	0
database	6642	ZINC08400929	0
database	5478	ZINC08424949	0
database	2830	ZINC00625689	0
database	638	ZINC00181552	0
database	2020	ZINC09123695	0
database	217	ZINC08442060	0
database	4597	ZINC08430546	0
database	4814	ZINC00982884	0
database	229	ZINC00662497	0

database	3592	ZINC00194823	0
database	6299	ZINC00985880	0
database	7057	ZINC08400507	0
database	4476	ZINC08431064	0
database	5401	ZINC01024810	0
database	1437	ZINC00721236	0
database	6153	ZINC08411438	0
database	4568	ZINC00667497	0
database	1044	ZINC00702714	0
database	5660	ZINC00691663	0
database	931	ZINC08441013	0
database	7154	ZINC02135584	0
database	2818	ZINC08437174	0
database	6259	ZINC00987205	0
database	269	ZINC08442007	0
database	580	ZINC08441625	0
database	1792	ZINC02169761	0
database	4347	ZINC00703575	0
database	125	ZINC00664787	0
database	6625	ZINC08402226	0
database	3962	ZINC00670790	0
database	530	ZINC15952844	0
database	3787	ZINC01802675	0
database	5359	ZINC08425627	0
database	3853	ZINC00628237	0
database	422	ZINC00881856	0
database	4208	ZINC08431837	0

database	4076	ZINC08432386	0
database	3595	ZINC08434874	0
database	6900	ZINC08400748	0
database	31	ZINC18141403	0
database	5285	ZINC00661030	0
database	2278	ZINC08438679	0
database	3106	ZINC00887442	0
database	4442	ZINC00984491	0
database	405	ZINC00881982	0
database	3815	ZINC08433051	0
database	2730	ZINC08437429	0
database	4966	ZINC08872338	0
database	1752	ZINC02503421	0
database	341	ZINC08441930	0
database	3799	ZINC00723830	0
database	5659	ZINC00691735	0
database	5030	ZINC08426998	0
database	4953	ZINC00291192	0
database	1378	ZINC08440084	0
database	2951	ZINC06144544	0
database	6262	ZINC00987198	0
database	4802	ZINC02144341	0
database	6415	ZINC01023018	0
database	3060	ZINC01018842	0
database	3855	ZINC00142814	0
database	6231	ZINC00988057	0
database	6494	ZINC19369491	0

database	6784	ZINC00687352	0
database	3978	ZINC08432703	0
database	2887	ZINC08437076	0
database	5448	ZINC08425141	0
database	5603	ZINC00692735	0
database	40	ZINC08442278	0
database	5842	ZINC08413942	0
database	6780	ZINC00687359	0
database	6722	ZINC00687449	0
database	7024	ZINC08400548	0
database	3303	ZINC08435580	0
database	2977	ZINC00645571	0
database	4043	ZINC08432500	0
database	2758	ZINC00646579	0
database	2809	ZINC08437199	0
database	3179	ZINC00897144	0
database	5571	ZINC02057087	0
database	1002	ZINC00702733	0
database	1925	ZINC00627348	0
database	5482	ZINC08424944	0
database	4354	ZINC00991573	0
database	6707	ZINC00687496	0
database	4200	ZINC00985041	0
database	5856	ZINC08413894	0
database	561	ZINC00703108	0
database	3670	ZINC08433310	0
database	5104	ZINC08426767	0

database	5511	ZINC08424822	0
database	4571	ZINC00845249	0
database	6496	ZINC19369493	0
database	3780	ZINC08433139	0
database	4380	ZINC00703533	0
database	1506	ZINC00630196	0
database	316	ZINC08441966	0
database	4044	ZINC08432499	0
database	927	ZINC08441017	0
database	2706	ZINC01010959	0
database	3056	ZINC08436816	0
database	6379	ZINC08408020	0
database	1266	ZINC08440218	0
database	391	ZINC02064386	0
database	4520	ZINC02083621	0
database	6672	ZINC00687619	0
database	1976	ZINC02165216	0
database	6593	ZINC00210307	0
database	2120	ZINC01413446	0
database	2412	ZINC08438345	0
database	5652	ZINC08416873	0
database	1954	ZINC00627097	0
database	6219	ZINC08411267	0
database	6629	ZINC01021485	0
database	1530	ZINC00717417	0
database	280	ZINC09122187	0
database	5969	ZINC08413387	0

database	1483	ZINC00189848	0
database	833	ZINC08441266	0
database	4	ZINC01700294	0
database	5341	ZINC00629649	0
database	264	ZINC08442009	0
database	6828	ZINC08400805	0
database	5946	ZINC08413415	0
database	1313	ZINC00702426	0
database	5352	ZINC08425653	0
database	1517	ZINC08439747	0
database	557	ZINC00878056	0
database	233	ZINC08442040	0
database	3016	ZINC02155726	0
database	5459	ZINC00833678	0
database	1994	ZINC02165080	0
database	5173	ZINC08426403	0
database	2856	ZINC00646445	0
database	6904	ZINC00686500	0
database	6991	ZINC01008241	0
database	627	ZINC00857173	0
database	3503	ZINC08435141	0
database	5456	ZINC08425064	0
database	4515	ZINC02568448	0
database	6925	ZINC08400709	0
database	1718	ZINC08439307	0
database	1912	ZINC08438900	0
database	3525	ZINC08435046	0

database	508	ZINC00881528	0
database	4003	ZINC01896082	0
database	6100	ZINC08411605	0
database	1100	ZINC05918675	0
database	6937	ZINC02142314	0
database	1714	ZINC00969280	0
database	2723	ZINC08437432	0
database	1794	ZINC08439173	0
database	6929	ZINC02142488	0
database	5554	ZINC08424471	0
database	1610	ZINC00717199	0
database	3495	ZINC00678505	0
database	3127	ZINC08435641	0
database	5226	ZINC02179837	0
database	4541	ZINC12462013	0
database	5075	ZINC08426799	0
database	1678	ZINC08439378	0
database	4016	ZINC00998410	0
database	3833	ZINC03241184	0
database	2447	ZINC08438182	0
database	2587	ZINC01011153	0
database	2654	ZINC08437659	0
database	1585	ZINC08439611	0
database	3616	ZINC00809007	0
database	1788	ZINC01013036	0
database	1	ZINC09365179	0
database	4546	ZINC08430705	0

database	2973	ZINC00645570	0
database	5328	ZINC00708344	0
database	3161	ZINC00907651	0
database	5621	ZINC00692502	0
database	1915	ZINC08438899	0
database	4933	ZINC00711857	0
database	504	ZINC00971827	0
database	783	ZINC00853635	0
database	4532	ZINC08430805	0
database	2492	ZINC00624365	0
database	519	ZINC00881458	0
database	230	ZINC00662500	0
database	249	ZINC08442026	0
database	6595	ZINC02565049	0
database	1918	ZINC00627359	0
database	4322	ZINC08431533	0
database	854	ZINC08441223	0
database	2584	ZINC01011157	0
database	5597	ZINC00692951	0
database	1895	ZINC00627458	0
database	1654	ZINC08439415	0
database	5836	ZINC08413952	0
database	5121	ZINC08426679	0
database	4531	ZINC08430809	0
database	5293	ZINC02060574	0
database	6156	ZINC08411433	0
database	1987	ZINC02165138	0

database	939	ZINC08440994	0
database	6720	ZINC02146196	0
database	1563	ZINC08439649	0
database	4363	ZINC00997529	0
database	1693	ZINC08439344	0
database	6818	ZINC08400808	0
database	4428	ZINC00730142	0
database	4243	ZINC02183169	0
database	7086	ZINC02252326	0
database	6515	ZINC00705660	0
database	4369	ZINC00703552	0
database	6611	ZINC00704802	0
database	6637	ZINC08400941	0
database	1685	ZINC06147567	0
database	1818	ZINC00683819	0
database	5159	ZINC02181246	0
database	3663	ZINC03462211	0
database	5879	ZINC08413878	0
database	2748	ZINC00646591	0
database	1227	ZINC00702501	0
database	2536	ZINC08437830	0
database	6386	ZINC08407966	0
database	5168	ZINC00642619	0
database	4964	ZINC00711719	0
database	6283	ZINC06300265	0
database	1509	ZINC00147704	0
database	6389	ZINC08407953	0

database	2739	ZINC08437421	0
database	1574	ZINC08439616	0
database	1170	ZINC00844382	0
database	3329	ZINC06156995	0
database	6718	ZINC00687459	0
database	5523	ZINC00628208	0
database	907	ZINC00849797	0
database	1048	ZINC00702710	0
database	6229	ZINC08411113	0
database	5994	ZINC08413182	0
database	2509	ZINC00624107	0
database	6336	ZINC00985574	0
database	2471	ZINC08438078	0
database	6031	ZINC08413100	0
database	5470	ZINC08424956	0
database	3831	ZINC08433015	0
database	5752	ZINC08414739	0
database	5379	ZINC04685512	0
database	2704	ZINC00694230	0
database	1514	ZINC13545844	0
database	5869	ZINC08413888	0
database	4968	ZINC08427418	0
database	3317	ZINC06135987	0
database	4782	ZINC00664739	0
database	4522	ZINC08430830	0
database	1409	ZINC00074217	0
database	4306	ZINC00997656	0

database	409	ZINC00971829	0
database	415	ZINC00881890	0
database	6365	ZINC15989997	0
database	6206	ZINC08411305	0
database	5629	ZINC00089147	0
database	4644	ZINC08430347	0
database	4388	ZINC00984707	0
database	3034	ZINC00645501	0
database	297	ZINC12416741	0
database	884	ZINC00851196	0
database	6284	ZINC00792716	0
database	6912	ZINC02142792	0
database	237	ZINC08442036	0
database	4239	ZINC09012355	0
database	503	ZINC00881538	0
database	3632	ZINC02067541	0
database	1500	ZINC00630241	0
database	4118	ZINC08432214	0
database	5519	ZINC08424661	0
database	4198	ZINC02084706	0
database	3527	ZINC00127467	0
database	6630	ZINC19014718	0
database	526	ZINC15952846	0
database	299	ZINC08441979	0
database	23	ZINC05286115	0
database	2572	ZINC02162019	0
database	4605	ZINC02140649	0

database	3717	ZINC01822612	0
database	2697	ZINC01010971	0
database	3452	ZINC00662865	0
database	1090	ZINC00702692	0
database	1529	ZINC00381903	0
database	4486	ZINC00480247	0
database	3181	ZINC00888510	0
database	998	ZINC00848995	0
database	773	ZINC08441387	0
database	842	ZINC08441239	0
database	1808	ZINC02169139	0
database	6319	ZINC00985635	0
database	850	ZINC05313297	0
database	2369	ZINC00625480	0
database	3785	ZINC01802704	0
database	7113	ZINC08400451	0
database	5945	ZINC08413415	0
database	5947	ZINC08413411	0
database	4911	ZINC00712042	0
database	3642	ZINC06904335	0
database	5221	ZINC09271506	0
database	4940	ZINC01029954	0
database	2798	ZINC00646453	0
database	6772	ZINC08400814	0
database	2355	ZINC00625733	0
database	3082	ZINC00884345	0
database	2375	ZINC08438528	0

database	849	ZINC05313299	0
database	5610	ZINC00692629	0
database	6233	ZINC15985639	0
database	1169	ZINC00666396	0
database	4422	ZINC08431335	0
database	1790	ZINC02169772	0
database	4403	ZINC08431405	0
database	656	ZINC13123054	0
database	2168	ZINC01413501	0
database	6907	ZINC00686486	0
database	5484	ZINC08424917	0
database	2981	ZINC00645569	0
database	348	ZINC08441924	0
database	1219	ZINC08440404	0
database	7069	ZINC01008154	0
database	5172	ZINC08426407	0
database	3828	ZINC00719446	0
database	7201	ZINC13111995	0
database	1357	ZINC00441990	0
database	5216	ZINC08899112	0
database	6376	ZINC08408062	0
database	1460	ZINC00717893	0
database	1143	ZINC08440596	0
database	6367	ZINC08409920	0
database	2401	ZINC08438403	0
database	2650	ZINC08437661	0
database	6827	ZINC00687240	0

database	6280	ZINC06300277	0
database	5204	ZINC08426280	0
database	1632	ZINC04113089	0
database	1920	ZINC00627361	0
database	2332	ZINC08438563	0
database	124	ZINC00664784	0
database	1594	ZINC00717214	0
database	1522	ZINC00643559	0
database	741	ZINC00703052	0
database	3245	ZINC05576958	0
database	6028	ZINC08413100	0
database	6141	ZINC08411460	0
database	5612	ZINC01010657	0
database	806	ZINC06135667	0
database	6507	ZINC19369484	0
database	4575	ZINC00667466	0
database	5770	ZINC08414455	0
database	3339	ZINC08435573	0
database	6418	ZINC08407217	0
database	7107	ZINC02157833	0
database	6034	ZINC08413085	0
database	245	ZINC08442027	0
database	6959	ZINC08400659	0
database	1024	ZINC08440772	0
database	2530	ZINC08437858	0
database	3066	ZINC06444433	0
database	5363	ZINC00708250	0

database	2446	ZINC00669161	0
database	780	ZINC08441364	0
database	6582	ZINC00290008	0
database	71	ZINC00633958	0
database	2158	ZINC01413516	0
database	6762	ZINC00687398	0
database	1453	ZINC09072303	0
database	308	ZINC08441972	0
database	1465	ZINC00717759	0
database	5109	ZINC08426765	0
database	6437	ZINC01022876	0
database	22	ZINC05286115	0
database	1033	ZINC00702715	0
database	6102	ZINC08411605	0
database	5632	ZINC00692332	0
database	6623	ZINC08402425	0
database	974	ZINC00851794	0
database	5308	ZINC08425897	0
database	3912	ZINC00710395	0
database	6841	ZINC01008644	0
database	462	ZINC00630488	0
database	821	ZINC08897840	0
database	258	ZINC08442014	0
database	4943	ZINC08427484	0
database	2036	ZINC02164852	0
database	1815	ZINC08439126	0
database	2196	ZINC08438760	0

database	1494	ZINC00645899	0
database	4880	ZINC08427631	0
database	573	ZINC01019940	0
database	1262	ZINC00702450	0
database	6581	ZINC00290007	0
database	1488	ZINC00645913	0
database	3283	ZINC08435583	0
database	1568	ZINC00629392	0
database	3549	ZINC08435017	0
database	2963	ZINC00645577	0
database	4920	ZINC08427580	0
database	4110	ZINC00668744	0
database	636	ZINC00181717	0
database	4075	ZINC08432392	0
database	190	ZINC04166616	0
database	1206	ZINC08440459	0
database	182	ZINC00664549	0
database	4080	ZINC00668988	0
database	408	ZINC00881937	0
database	3983	ZINC02085869	0
database	6303	ZINC00985862	0
database	5090	ZINC08426780	0
database	3261	ZINC00862461	0
database	1907	ZINC01019553	0
database	1779	ZINC08439206	0
database	5494	ZINC00296010	0
database	5271	ZINC00836245	0

database	4453	ZINC09065134	0
database	154	ZINC08442083	0
database	378	ZINC02064399	0
database	5437	ZINC00728824	0
database	646	ZINC00971705	0
database	5384	ZINC08425499	0
database	521	ZINC08441650	0
database	3143	ZINC06280098	0
database	2516	ZINC00623993	0
database	3664	ZINC00730283	0
database	241	ZINC08442030	0
database	4438	ZINC00997286	0
database	2429	ZINC00669320	0
database	5087	ZINC08426781	0
database	380	ZINC08441902	0
database	2384	ZINC00625323	0
database	2417	ZINC08438288	0
database	5968	ZINC08413387	0
database	3909	ZINC01801657	0
database	5419	ZINC08425382	0
database	6273	ZINC04167868	0
database	4085	ZINC00985788	0
database	3473	ZINC06444891	0
database	5344	ZINC00298231	0
database	4951	ZINC00711748	0
database	4151	ZINC00668429	0
database	2687	ZINC08437516	0

database	913	ZINC08441036	0
database	3244	ZINC05576958	0
database	832	ZINC08441267	0
database	5884	ZINC08413868	0
database	2619	ZINC13282878	0
database	4364	ZINC00703551	0
database	4976	ZINC08427361	0
database	804	ZINC00702988	0
database	728	ZINC00703053	0
database	3412	ZINC02071322	0
database	801	ZINC00702987	0
database	6998	ZINC08400611	0
database	6854	ZINC00686755	0
database	2255	ZINC06445943	0
database	6947	ZINC08400674	0
database	902	ZINC00856250	0
database	1643	ZINC04113086	0
database	898	ZINC09357898	0
database	4779	ZINC08430132	0
database	6761	ZINC00687399	0
database	5215	ZINC08899112	0
database	367	ZINC08441911	0
database	5863	ZINC08413890	0
database	6217	ZINC04407449	0
database	1549	ZINC08439662	0
database	6617	ZINC19370782	0
database	2926	ZINC02156084	0

database	6144	ZINC08411455	0
database	4904	ZINC08427628	0
database	2226	ZINC00626552	0
database	191	ZINC00664409	0
database	6602	ZINC00704834	0
database	2472	ZINC08438077	0
database	267	ZINC08442007	0
database	918	ZINC08441032	0
database	4537	ZINC08430760	0
database	3125	ZINC06658422	0
database	6209	ZINC08411277	0
database	933	ZINC08441012	0
database	4116	ZINC00668731	0
database	1184	ZINC00702582	0
database	1341	ZINC08440140	0
database	5082	ZINC08426790	0
database	5487	ZINC08424915	0
database	3690	ZINC08433258	0
database	4014	ZINC02087800	0
database	5149	ZINC08426516	0
database	639	ZINC00181552	0
database	2832	ZINC00625713	0
database	3059	ZINC08436813	0
database	6851	ZINC08400778	0
database	3771	ZINC00724006	0
database	6443	ZINC18043169	0
database	4876	ZINC08427652	0

database	838	ZINC00851613	0
database	3752	ZINC09301883	0
database	90	ZINC08442121	0
database	5477	ZINC08424949	0
database	7193	ZINC00668898	0
database	4405	ZINC08431394	0
database	6753	ZINC00687406	0
database	1618	ZINC00350684	0
database	751	ZINC00617102	0
database	1937	ZINC08438888	0
database	3513	ZINC00826507	0
database	144	ZINC00664717	0
database	2651	ZINC08437661	0
database	6366	ZINC08409920	0
database	4481	ZINC06663980	0
database	889	ZINC06556763	0
database	4997	ZINC08427301	0
database	4922	ZINC08427578	0
database	3832	ZINC00948789	0
database	1248	ZINC08440285	0
database	3840	ZINC01801676	0
database	3349	ZINC00881818	0
database	5350	ZINC00647427	0
database	3159	ZINC00915426	0
database	5732	ZINC08415066	0
database	3743	ZINC08433168	0
database	559	ZINC02064359	0

database	2449	ZINC00624555	0
database	2976	ZINC00645571	0
database	1125	ZINC08440661	0
database	2620	ZINC01011083	0
database	3744	ZINC00633668	0
database	3911	ZINC00710394	0
database	5026	ZINC03109482	0
database	652	ZINC13123303	0
database	971	ZINC08440874	0
database	554	ZINC00703107	0
database	1084	ZINC00702689	0
database	1804	ZINC01012866	0
database	5885	ZINC08413868	0
database	5399	ZINC08425458	0
database	6606	ZINC08403043	0
database	4559	ZINC02140793	0
database	3386	ZINC08435509	0
database	5039	ZINC08426909	0
database	1194	ZINC06445251	0
database	4350	ZINC00703576	0
database	1148	ZINC08440584	0
database	488	ZINC00650712	0
database	6847	ZINC02143713	0
database	6901	ZINC00686529	0
database	5579	ZINC12413071	0
database	1003	ZINC00702733	0
database	6923	ZINC08400711	0

database	2868	ZINC00646443	0
database	287	ZINC00633740	0
database	6934	ZINC02142356	0
database	5279	ZINC00836256	0
database	4529	ZINC00712119	0
database	5003	ZINC03079865	0
database	4172	ZINC00998037	0
database	3968	ZINC08432745	0
database	756	ZINC08441398	0
database	2193	ZINC01413490	0
database	5492	ZINC02058434	0
database	298	ZINC08441979	0
database	4870	ZINC00728968	0
database	4845	ZINC08429957	0
database	122	ZINC08442096	0
database	7075	ZINC08400479	0
database	3555	ZINC00123806	0
database	2207	ZINC00626750	0
database	4883	ZINC08427631	0
database	311	ZINC08441970	0
database	4287	ZINC00845724	0
database	3769	ZINC01017574	0
database	200	ZINC08442070	0
database	964	ZINC06445775	0
database	4947	ZINC01029906	0
database	3405	ZINC02959734	0
database	3786	ZINC00947657	0

database	127	ZINC00664768	0
database	1916	ZINC08438899	0
database	137	ZINC00664738	0
database	1377	ZINC08440085	0
database	277	ZINC01107287	0
database	5006	ZINC00711107	0
database	3727	ZINC09007850	0
database	6020	ZINC08413106	0
database	1599	ZINC00717215	0
database	4081	ZINC00668990	0
database	4142	ZINC00707048	0
database	5046	ZINC08426845	0
database	6117	ZINC00688050	0
database	3943	ZINC00710252	0
database	6725	ZINC00687445	0
database	1819	ZINC00683816	0
database	7194	ZINC02140159	0
database	3496	ZINC00678504	0
database	7102	ZINC08400463	0
database	147	ZINC00664707	0
database	617	ZINC08441597	0
database	2848	ZINC08437141	0
database	2621	ZINC00622905	0
database	5146	ZINC01026688	0
database	6554	ZINC00062357	0
database	1528	ZINC00643493	0
database	4133	ZINC01895227	0

database	977	ZINC00702759	0
database	2212	ZINC00669966	0
database	1274	ZINC00702446	0
database	3665	ZINC01019683	0
database	2989	ZINC00645564	0
database	4797	ZINC02144399	0
database	1324	ZINC17160093	0
database	1608	ZINC00717217	0
database	1777	ZINC02169920	0
database	7077	ZINC08400478	0
database	4769	ZINC08430147	0
database	471	ZINC00881558	0
database	5798	ZINC08413985	0
database	5585	ZINC08417899	0
database	2929	ZINC00693690	0
database	480	ZINC00703149	0
database	399	ZINC02064380	0
database	4072	ZINC00483878	0
database	5617	ZINC00692525	0
database	4623	ZINC00479791	0
database	1467	ZINC08439872	0
database	5602	ZINC00367778	0
database	2507	ZINC00624112	0
database	3324	ZINC06177076	0
database	6012	ZINC08413110	0
database	1435	ZINC13286748	0
database	1754	ZINC00715097	0

database	4051	ZINC00669405	0
database	2968	ZINC00645574	0
database	4404	ZINC00984651	0
database	6007	ZINC08413115	0
database	138	ZINC00664738	0
database	5709	ZINC08415472	0
database	4631	ZINC08430357	0
database	3248	ZINC00864421	0
database	6963	ZINC02136079	0
database	6548	ZINC00299345	0
database	2899	ZINC09186824	0
database	2952	ZINC06144544	0
database	6136	ZINC08411472	0
database	5555	ZINC08424471	0
database	2438	ZINC00624580	0
database	3282	ZINC08435584	0
database	5469	ZINC08424956	0
database	2198	ZINC01413491	0
database	2920	ZINC08436959	0
database	7127	ZINC00676396	0
database	778	ZINC00703038	0
database	2745	ZINC00646592	0
database	4280	ZINC08431726	0
database	5429	ZINC00708065	0
database	4793	ZINC00664652	0
database	4408	ZINC00984603	0
database	3207	ZINC00976164	0

database	214	ZINC09281351	0
database	384	ZINC02064395	0
database	3940	ZINC08432818	0
database	2688	ZINC08437514	0
database	3139	ZINC00838409	0
database	6207	ZINC08411289	0
database	5808	ZINC08413980	0
database	1131	ZINC08440596	0
database	1023	ZINC08440772	0
database	6500	ZINC00705672	0
database	5310	ZINC08425806	0
database	3053	ZINC08436826	0
database	6193	ZINC08411329	0
database	4942	ZINC01029930	0
database	3272	ZINC00625938	0
database	2636	ZINC00622868	0
database	2925	ZINC02156084	0
database	1955	ZINC00627098	0
database	3497	ZINC00678489	0
database	4179	ZINC08431925	0
database	6615	ZINC19370782	0
database	6025	ZINC08413106	0
database	1828	ZINC08439088	0
database	6976	ZINC06023577	0
database	990	ZINC08440856	0
database	3342	ZINC08435565	0
database	2633	ZINC00622883	0

database	6446	ZINC19119246	0
database	3633	ZINC00974121	0
database	5499	ZINC00707570	0
database	633	ZINC00241845	0
database	2043	ZINC01413485	0
database	2949	ZINC06144561	0
database	5551	ZINC00727720	0
database	5072	ZINC00710579	0
database	3718	ZINC08433188	0
database	6372	ZINC02150032	0
database	3403	ZINC08435476	0
database	6747	ZINC00687412	0
database	942	ZINC08440987	0
database	1556	ZINC08439654	0
database	2917	ZINC02156129	0
database	919	ZINC08441032	0
database	2876	ZINC00646270	0
database	3597	ZINC08434874	0
database	1318	ZINC00702427	0
database	1053	ZINC00702700	0
database	2847	ZINC08437141	0
database	5468	ZINC08424956	0
database	6327	ZINC00985621	0
database	4817	ZINC00729083	0
database	3716	ZINC08433211	0
database	1014	ZINC00702731	0
database	2154	ZINC01413497	0

database	6509	ZINC00705668	0
database	5436	ZINC00146770	0
database	6449	ZINC19798079	0
database	6510	ZINC19369473	0
database	6149	ZINC08411444	0
database	1150	ZINC08440584	0
database	6783	ZINC00687353	0
database	4101	ZINC08432295	0
database	1172	ZINC00844300	0
database	5422	ZINC08425371	0
database	6187	ZINC08411375	0
database	5283	ZINC00661027	0
database	315	ZINC08441966	0
database	5320	ZINC08425748	0
database	268	ZINC08442007	0
database	2506	ZINC00624115	0
database	7177	ZINC00866580	0
database	347	ZINC08441924	0
database	2483	ZINC08395141	0
database	2088	ZINC02164732	0
database	2782	ZINC00646487	0
database	5552	ZINC08424473	0
database	350	ZINC06195024	0
database	920	ZINC08441032	0
database	3345	ZINC02951289	0
database	3541	ZINC00677049	0
database	3401	ZINC08435483	0

database	3369	ZINC00872961	0
database	6390	ZINC08407951	0
database	1038	ZINC00702718	0
database	6027	ZINC08413100	0
database	3004	ZINC02155841	0
database	3880	ZINC06135910	0
database	5698	ZINC08415481	0
database	6062	ZINC08413048	0
database	658	ZINC08441538	0
database	6881	ZINC00686616	0
database	4746	ZINC00729396	0
database	3533	ZINC08435035	0
database	3644	ZINC08433435	0
database	7046	ZINC08400515	0
database	4637	ZINC08430354	0
database	5490	ZINC08424914	0
database	1774	ZINC15941858	0
database	4700	ZINC02065648	0
database	579	ZINC08441625	0
database	1153	ZINC08440584	0
database	523	ZINC07077628	0
database	24	ZINC00984053	0
database	5153	ZINC02060874	0
database	7203	ZINC13111995	0
database	4062	ZINC00669376	0
database	5147	ZINC08426531	0
database	1984	ZINC02165183	0

database	3299	ZINC08818093	0
database	5616	ZINC08417563	0
database	908	ZINC06144687	0
database	4849	ZINC00982641	0
database	4594	ZINC00996457	0
database	2145	ZINC01413489	0
database	4013	ZINC01896127	0
database	6810	ZINC00687284	0
database	4415	ZINC08431354	0
database	304	ZINC08441974	0
database	6610	ZINC08402916	0
database	1979	ZINC02165211	0
database	3848	ZINC08433010	0
database	6454	ZINC00705796	0
database	5079	ZINC08426798	0
database	1455	ZINC08439891	0
database	4662	ZINC02065756	0
database	1698	ZINC08439340	0
database	704	ZINC08441459	0
database	6006	ZINC08413115	0
database	7027	ZINC00676702	0
database	48	ZINC08442271	0
database	6177	ZINC08411403	0
database	5454	ZINC08425065	0
database	5295	ZINC08425972	0
database	351	ZINC06195024	0
database	4407	ZINC08431387	0

database	1953	ZINC00627096	0
database	717	ZINC15884980	0
database	6809	ZINC00687286	0
database	6635	ZINC00687880	0
database	567	ZINC08441630	0
database	7224	ZINC00675037	0
database	1501	ZINC00645842	0
database	338	ZINC08441931	0
database	3353	ZINC00975931	0
database	2529	ZINC08437859	0
database	6167	ZINC08411415	0
database	1213	ZINC08440415	0
database	2443	ZINC08438198	0
database	4341	ZINC00703577	0
database	2581	ZINC08437721	0
database	5174	ZINC08426400	0
database	4581	ZINC00667457	0
database	1977	ZINC08438828	0
database	17	ZINC01240782	0
database	2671	ZINC04167654	0
database	1985	ZINC02165177	0
database	1178	ZINC00702587	0
database	3715	ZINC00727934	0
database	2013	ZINC09331361	0
database	1232	ZINC06159425	0
database	5580	ZINC00793736	0
database	4216	ZINC00706851	0

database	2418	ZINC08438288	0
database	1770	ZINC08439234	0
database	6972	ZINC02141989	0
database	6040	ZINC08413079	0
database	5870	ZINC08413888	0
database	4576	ZINC00667466	0
database	968	ZINC08440877	0
database	3204	ZINC00976193	0
database	2481	ZINC05985899	0
database	4617	ZINC08430404	0
database	1827	ZINC08439093	0
database	2409	ZINC00624710	0
database	1620	ZINC08439536	0
database	3365	ZINC02071920	0
database	6714	ZINC02146199	0
database	6363	ZINC15989997	0
database	3098	ZINC00883986	0
database	5397	ZINC17028642	0
database	6685	ZINC00687594	0
database	3205	ZINC08435608	0
database	6905	ZINC08400745	0
database	4497	ZINC16916881	0
database	2486	ZINC08438005	0
database	6520	ZINC00064448	0
database	131	ZINC00664754	0
database	5386	ZINC08425486	0
database	219	ZINC13161525	0

database	5578	ZINC12413071	0
database	3745	ZINC06144032	0
database	7168	ZINC00675546	0
database	227	ZINC00803800	0
database	94	ZINC08442118	0
database	5747	ZINC08414751	0
database	1375	ZINC08440085	0
database	1311	ZINC00702426	0
database	5933	ZINC08413446	0
database	336	ZINC08441932	0
database	2064	ZINC01413469	0
database	3700	ZINC09271700	0
database	7035	ZINC00999322	0
database	4167	ZINC00998059	0
database	6405	ZINC08407873	0
database	2843	ZINC08437144	0
database	5672	ZINC08415497	0
database	5929	ZINC08413491	0
database	3186	ZINC08435614	0
database	6413	ZINC08407470	0
database	1531	ZINC00643478	0
database	1129	ZINC00845776	0
database	6678	ZINC00687612	0
database	6808	ZINC02146018	0
database	5474	ZINC08424954	0
database	1963	ZINC08438837	0
database	3492	ZINC00655195	0

database	3800	ZINC00723830	0
database	4852	ZINC02738498	0
database	3483	ZINC00655457	0
database	5317	ZINC00647646	0
database	6116	ZINC08411529	0
database	1993	ZINC02165086	0
database	4355	ZINC00991573	0
database	1461	ZINC00717893	0
database	6835	ZINC08400795	0
database	2703	ZINC01891126	0
database	3726	ZINC09007850	0
database	6448	ZINC06844896	0
database	6916	ZINC08400717	0
database	4843	ZINC00995929	0
database	1233	ZINC06159424	0
database	3489	ZINC02070213	0
database	4583	ZINC00667343	0
database	786	ZINC09070021	0
database	2666	ZINC00622718	0
database	2733	ZINC08437428	0
database	2497	ZINC00624174	0
database	5547	ZINC00707250	0
database	4978	ZINC00711393	0
database	3332	ZINC08435576	0
database	6293	ZINC00789915	0
database	5751	ZINC08414743	0
database	115	ZINC08442106	0

database	2677	ZINC02159745	0
database	6236	ZINC13132811	0
database	3388	ZINC08435505	0
database	3640	ZINC06904335	0
database	7145	ZINC00675688	0
database	5287	ZINC08426008	0
database	681	ZINC00648858	0
database	722	ZINC01019938	0
database	2643	ZINC08437670	0
database	5461	ZINC00728711	0
database	121	ZINC08442096	0
database	2494	ZINC00624347	0
database	2044	ZINC01413485	0
database	6842	ZINC02143787	0
database	6466	ZINC00705793	0
database	4596	ZINC08430547	0
database	3721	ZINC02106974	0
database	815	ZINC16956755	0
database	2913	ZINC00693771	0
database	4503	ZINC01799356	0
database	2067	ZINC01414783	0
database	455	ZINC08441863	0
database	2695	ZINC00646767	0
database	7217	ZINC00668798	0
database	2646	ZINC08437668	0
database	5748	ZINC08414751	0
database	134	ZINC00664757	0

database	1960	ZINC08438852	0
database	6874	ZINC00686677	0
database	5935	ZINC08413446	0
database	5640	ZINC00692257	0
database	3253	ZINC06406259	0
database	4165	ZINC00668173	0
database	2576	ZINC08437723	0
database	4397	ZINC00984667	0
database	3655	ZINC09045651	0
database	6782	ZINC00687355	0
database	4570	ZINC00996523	0
database	2146	ZINC01413489	0
database	3221	ZINC00862950	0
database	5539	ZINC08424527	0
database	2464	ZINC08438088	0
database	1688	ZINC08439360	0
database	2718	ZINC08437442	0
database	4090	ZINC13155029	0
database	6326	ZINC00985621	0
database	1256	ZINC02062113	0
database	3863	ZINC08432985	0
database	6732	ZINC00687435	0
database	449	ZINC00881652	0
database	3862	ZINC02941096	0
database	1527	ZINC08439724	0
database	794	ZINC00853504	0
database	7147	ZINC02805924	0

database	295	ZINC08441981	0
database	5618	ZINC00188882	0
database	831	ZINC08441276	0
database	954	ZINC06300225	0
database	2644	ZINC08437670	0
database	6927	ZINC08400706	0
database	1677	ZINC08439378	0
database	2153	ZINC01413497	0
database	3814	ZINC08433052	0
database	6660	ZINC00687667	0
database	150	ZINC00664709	0
database	6616	ZINC19370782	0
database	1051	ZINC00702699	0
database	195	ZINC04167831	0
database	6123	ZINC08411510	0
database	6023	ZINC08413106	0
database	6853	ZINC08400776	0
database	3083	ZINC00884350	0
database	5327	ZINC00708343	0
database	1673	ZINC08439382	0
database	2454	ZINC00624470	0
database	7172	ZINC02135395	0
database	5446	ZINC00728809	0
database	781	ZINC08441363	0
database	4384	ZINC08431457	0
database	1820	ZINC00683816	0
database	4091	ZINC13155029	0

database	7222	ZINC00668775	0
database	145	ZINC00664719	0
database	6941	ZINC01008317	0
database	565	ZINC06474972	0
database	5980	ZINC08413327	0
database	894	ZINC06445734	0
database	5043	ZINC00710816	0
database	2961	ZINC00645576	0
database	2038	ZINC01414771	0
database	1297	ZINC00702434	0
database	1850	ZINC02168334	0
database	1185	ZINC08440510	0
database	5604	ZINC00692735	0
database	5538	ZINC01018116	0
database	129	ZINC08670819	0
database	3588	ZINC08434881	0
database	1941	ZINC08438878	0
database	535	ZINC15952845	0
database	3646	ZINC00702231	0
database	5619	ZINC01010635	0
database	3052	ZINC08436829	0
database	6278	ZINC04168845	0
database	4137	ZINC00214465	0
database	5866	ZINC08413888	0
database	2596	ZINC08437703	0
database	2833	ZINC00625713	0
database	3307	ZINC08817703	0

database	1234	ZINC06159424	0
database	1861	ZINC08438963	0
database	1166	ZINC00666409	0
database	1470	ZINC08439871	0
database	7166	ZINC02135472	0
database	4527	ZINC00845103	0
database	6014	ZINC08413110	0
database	5556	ZINC08424452	0
database	2082	ZINC01414785	0
database	7081	ZINC00920498	0
database	811	ZINC08914319	0
database	1066	ZINC00702697	0
database	6583	ZINC19335442	0
database	3399	ZINC06473864	0
database	4000	ZINC00670398	0
database	2058	ZINC01414774	0
database	3988	ZINC08432679	0
database	1458	ZINC00717892	0
database	1388	ZINC08440005	0
database	2675	ZINC06283020	0
database	3438	ZINC00663219	0
database	6756	ZINC02146166	0
database	4913	ZINC08427618	0
database	2317	ZINC00625874	0
database	2238	ZINC00626518	0
database	3444	ZINC00627140	0
database	3522	ZINC00195057	0

database	3169	ZINC08435620	0
database	4891	ZINC08427630	0
database	343	ZINC08441928	0
database	5078	ZINC08426798	0
database	1923	ZINC00627365	0
database	4195	ZINC00628515	0
database	3049	ZINC08436842	0
database	2285	ZINC08438676	0
database	157	ZINC08442081	0
database	2441	ZINC08438204	0
database	3360	ZINC08435555	0
database	2632	ZINC00622883	0
database	4150	ZINC00668427	0
database	1320	ZINC17160093	0
database	335	ZINC08441933	0
database	3460	ZINC02070559	0
database	5520	ZINC01024296	0
database	1005	ZINC00702734	0
database	4089	ZINC13155029	0
database	3284	ZINC19790623	0
database	4982	ZINC01051179	0
database	1136	ZINC08440596	0
database	3448	ZINC00662900	0
database	5628	ZINC08417514	0
database	4776	ZINC00996059	0
database	5463	ZINC01028537	0
database	2270	ZINC08438685	0

database	528	ZINC15952847	0
database	1008	ZINC00702729	0
database	2387	ZINC08438458	0
database	4593	ZINC00996457	0
database	2096	ZINC01414752	0
database	3311	ZINC06445233	0
database	2659	ZINC00622796	0
database	6679	ZINC00687604	0
database	4245	ZINC01801816	0
database	2488	ZINC02162881	0
database	4916	ZINC00712027	0
database	4266	ZINC00984956	0
database	678	ZINC08441495	0
database	4309	ZINC01895139	0
database	732	ZINC00703050	0
database	1155	ZINC08440584	0
database	3896	ZINC08432900	0
database	5473	ZINC08424954	0
database	1147	ZINC08440584	0
database	363	ZINC08441913	0
database	3316	ZINC06136516	0
database	2962	ZINC00645576	0
database	4676	ZINC00665334	0
database	3058	ZINC02184322	0
database	3620	ZINC02067674	0
database	2104	ZINC01413449	0
database	4375	ZINC00703541	0

database	4121	ZINC00985540	0
database	1347	ZINC08440137	0
database	3563	ZINC00675873	0
database	5648	ZINC00691907	0
database	594	ZINC08441618	0
database	3390	ZINC00793824	0
database	3666	ZINC00730199	0
database	3562	ZINC00675873	0
database	4490	ZINC08768631	0
database	2169	ZINC01413501	0
database	1216	ZINC08440410	0
database	5694	ZINC08415481	0
database	5161	ZINC02060868	0
database	6422	ZINC08407210	0
database	3479	ZINC00655499	0
database	6676	ZINC00687614	0
database	3255	ZINC06406689	0
database	1893	ZINC00627452	0
database	1446	ZINC09109829	0
database	6380	ZINC08408011	0
database	2541	ZINC00623493	0
database	5736	ZINC08414961	0
database	3128	ZINC00797523	0
database	2610	ZINC01011097	0
database	353	ZINC08441920	0
database	6249	ZINC00987519	0
database	696	ZINC08441467	0

database	3606	ZINC08434841	0
database	807	ZINC08997625	0
database	6519	ZINC08405096	0
database	300	ZINC08441979	0
database	4778	ZINC08430132	0
database	1839	ZINC08439064	0
database	3607	ZINC08434840	0
database	981	ZINC00702760	0
database	2757	ZINC00694035	0
database	6140	ZINC08411460	0
database	4372	ZINC08431472	0
database	466	ZINC00881569	0
database	2711	ZINC01019341	0
database	1744	ZINC01013161	0
database	3469	ZINC08435267	0
database	3720	ZINC08433185	0
database	3711	ZINC08433221	0
database	3577	ZINC08434915	0
database	2368	ZINC00625480	0
database	5543	ZINC02057328	0
database	5339	ZINC00647507	0
database	699	ZINC08441465	0
database	1182	ZINC00702583	0
database	3189	ZINC00897321	0
database	3190	ZINC00886465	0
database	4235	ZINC09012357	0
database	2726	ZINC08437431	0

database	5291	ZINC00462861	0
database	6667	ZINC08400865	0
database	355	ZINC08441920	0
database	2080	ZINC01414785	0
database	3476	ZINC00655537	0
database	1037	ZINC00702717	0
database	1210	ZINC02062340	0
database	4264	ZINC00997736	0
database	5802	ZINC08413985	0
database	3219	ZINC00862965	0
database	5113	ZINC00709985	0
database	6468	ZINC08406079	0
database	899	ZINC08996455	0
database	5599	ZINC08417740	0
database	4641	ZINC08430353	0
database	6734	ZINC00687430	0
database	5357	ZINC08425631	0
database	118	ZINC08442099	0
database	5118	ZINC08426690	0
database	5190	ZINC08426291	0
database	4257	ZINC00997739	0
database	6371	ZINC02150036	0
database	3093	ZINC00676205	0
database	4806	ZINC00664547	0
database	6085	ZINC08411694	0
database	7121	ZINC08400439	0
database	4511	ZINC01002905	0

database	224	ZINC00633803	0
database	2928	ZINC02497212	0
database	65	ZINC00633984	0
database	6276	ZINC04168846	0
database	7140	ZINC00675718	0
database	7017	ZINC00676744	0
database	3904	ZINC08432888	0
database	1776	ZINC01013092	0
database	459	ZINC00881590	0
database	7041	ZINC08400518	0
database	3268	ZINC00625908	0
database	1919	ZINC00627361	0
database	2046	ZINC02164817	0
database	4377	ZINC08431465	0
database	5528	ZINC00821013	0
database	1601	ZINC00717216	0
database	2737	ZINC08437422	0
database	2099	ZINC01413448	0
database	3547	ZINC02069178	0
database	5049	ZINC08426829	0
database	3590	ZINC08434877	0
database	4201	ZINC02483645	0
database	3373	ZINC00832944	0
database	6908	ZINC00686485	0
database	3676	ZINC09301975	0
database	5753	ZINC08414739	0
database	5540	ZINC00727806	0

database	3419	ZINC02071233	0
database	7080	ZINC08400475	0
database	522	ZINC06194338	0
database	5639	ZINC00367559	0
database	4506	ZINC01002972	0
database	3468	ZINC08435268	0
database	2534	ZINC08437838	0
database	2919	ZINC08436960	0
database	6051	ZINC08413061	0
database	2400	ZINC00624789	0
database	5163	ZINC12625376	0
database	4238	ZINC09012355	0
database	4176	ZINC08431951	0
database	5093	ZINC08426778	0
database	1081	ZINC08440708	0
database	1972	ZINC01011866	0
database	468	ZINC00881566	0
database	3847	ZINC08433010	0
database	7214	ZINC00668833	0
database	3764	ZINC02106715	0
database	3957	ZINC00710232	0
database	440	ZINC00631218	0
database	3104	ZINC00883777	0
database	139	ZINC08442089	0
database	613	ZINC08441601	0
database	2326	ZINC08438566	0
database	5066	ZINC08426812	0

database	4038	ZINC02279945	0
database	1419	ZINC09089086	0
database	2728	ZINC08437430	0
database	7184	ZINC08400315	0
database	599	ZINC02973109	0
database	7047	ZINC08400515	0
database	2078	ZINC01414785	0
database	372	ZINC02064406	0
database	2389	ZINC08438454	0
database	3464	ZINC00975294	0
database	1333	ZINC08440167	0
database	445	ZINC08441864	0
database	2311	ZINC00625900	0
database	3434	ZINC08435354	0
database	7031	ZINC00676673	0
database	5910	ZINC08413551	0
database	4586	ZINC00845174	0
database	148	ZINC00664707	0
database	2528	ZINC00623780	0
database	6094	ZINC00688112	0
database	4745	ZINC08430167	0
database	5772	ZINC08414449	0
database	7114	ZINC08400450	0
database	2817	ZINC00832608	0
database	130	ZINC08670818	0
database	2514	ZINC00624002	0
database	7026	ZINC01008177	0

database	7032	ZINC01008167	0
database	3291	ZINC01009442	0
database	5028	ZINC00887985	0
database	6700	ZINC00687534	0
database	6968	ZINC02136059	0
database	5393	ZINC00729270	0
database	4362	ZINC00997530	0
database	824	ZINC08897840	0
database	4847	ZINC08429954	0
database	11	ZINC08575396	0
database	4830	ZINC08430002	0
database	510	ZINC00881512	0
database	713	ZINC15884984	0
database	5766	ZINC08414480	0
database	6790	ZINC00687340	0
database	6512	ZINC19369473	0
database	3578	ZINC08434915	0
database	1176	ZINC00702595	0
database	4378	ZINC08431465	0
database	3947	ZINC00670845	0
database	4010	ZINC01896141	0
database	4472	ZINC08431130	0
database	2791	ZINC00646483	0
database	2533	ZINC08437838	0
database	7054	ZINC08400508	0
database	321	ZINC00061509	0
database	6298	ZINC00985880	0

database	5107	ZINC08426766	0
database	3120	ZINC00976276	0
database	6270	ZINC00987152	0
database	106	ZINC08442110	0
database	1644	ZINC04113086	0
database	4357	ZINC00703555	0
database	2772	ZINC02158869	0
database	2983	ZINC00645569	0
database	3374	ZINC00832943	0
database	3864	ZINC00715552	0
database	6183	ZINC08411399	0
database	698	ZINC08441465	0
database	6939	ZINC01008330	0
database	1569	ZINC00629389	0
database	3010	ZINC08436919	0
database	3075	ZINC00676223	0
database	6108	ZINC08411537	0
database	2589	ZINC01011135	0
database	828	ZINC08897821	0
database	953	ZINC06300227	0
database	2031	ZINC01414769	0
database	2006	ZINC08438782	0
database	6964	ZINC08400654	0
database	4005	ZINC00986134	0
database	2165	ZINC01413500	0
database	5439	ZINC00728825	0
database	7079	ZINC08400476	0

database	5781	ZINC08414187	0
database	3079	ZINC00884357	0
database	259	ZINC08442014	0
database	906	ZINC08441039	0
database	4335	ZINC00703588	0
database	3867	ZINC00078802	0
database	4053	ZINC00985983	0
database	6651	ZINC00687675	0
database	5483	ZINC00844685	0
database	2513	ZINC00624057	0
database	167	ZINC05918663	0
database	6601	ZINC02176539	0
database	1717	ZINC08439310	0
database	661	ZINC00858890	0
database	207	ZINC09210779	0
database	3789	ZINC01802668	0
database	5950	ZINC08413411	0
database	3230	ZINC06442961	0
database	4402	ZINC02178901	0
database	1975	ZINC01011863	0
database	171	ZINC00664588	0
database	1518	ZINC00630102	0
database	4365	ZINC00703551	0
database	979	ZINC00970788	0
database	1367	ZINC08440096	0
database	4314	ZINC00997616	0
database	788	ZINC08441354	0

database	6741	ZINC00687423	0
database	4220	ZINC06659935	0
database	1154	ZINC08440584	0
database	940	ZINC08440990	0
database	4669	ZINC00996257	0
database	601	ZINC08441614	0
database	4859	ZINC08429898	0
database	1597	ZINC00717215	0
database	720	ZINC00857358	0
database	4749	ZINC08430165	0
database	2681	ZINC00646871	0
database	3462	ZINC02070541	0
database	3564	ZINC00675873	0
database	3612	ZINC02067899	0
database	5696	ZINC08415481	0
database	2197	ZINC01413491	0
database	7059	ZINC08400499	0
database	2405	ZINC00624754	0
database	6982	ZINC00677009	0
database	5702	ZINC08415476	0
database	5375	ZINC00292672	0
database	7055	ZINC08400508	0
database	1647	ZINC00643339	0
database	5658	ZINC00691744	0
database	1133	ZINC08440596	0
database	3264	ZINC00862460	0
database	5031	ZINC08426994	0

database	2660	ZINC08437656	0
database	4326	ZINC08431530	0
database	1338	ZINC08440141	0
database	103	ZINC08442111	0
database	2682	ZINC08437580	0
database	4173	ZINC00706982	0
database	1113	ZINC08440679	0
database	6981	ZINC08400627	0
database	4282	ZINC08431718	0
database	7196	ZINC00668893	0
database	5425	ZINC08425367	0
database	6216	ZINC04473385	0
database	1974	ZINC00386658	0
database	5427	ZINC00708063	0
database	5493	ZINC08424904	0
database	6053	ZINC08413061	0
database	2715	ZINC00646599	0
database	4834	ZINC00982729	0
database	1866	ZINC02168082	0
database	1930	ZINC00627339	0
database	1173	ZINC00844301	0
database	3599	ZINC08434874	0
